# *WWOX/HIF1A* Balance Delineates Context-Dependent Molecular States in Breast Cancer Subtypes and Ovarian Carcinoma

**DOI:** 10.3390/ijms27156740

**Published:** 2026-07-28

**Authors:** Raneem Y. Hammouz, Kinga Maciejek, Andrzej K. Bednarek

**Affiliations:** Department of Molecular Carcinogenesis, Medical University of Lodz, Żeligowskiego 7/9, 90-752 Lodz, Poland; kinga.maciejek@umed.lodz.pl (K.M.); andrzej.bednarek@umed.lodz.pl (A.K.B.)

**Keywords:** *WWOX*, *HIF1A*, breast, ovarian, Notch, immune, hormone

## Abstract

*WWOX* and *HIF1A* are linked to hypoxia-driven metabolic and immune modulation in cancer. We examined whether the *WWOX/HIF1A* expression ratio acts as a context-dependent molecular integrator across breast cancer (BRCA) subtypes and ovarian carcinoma (OV). We analyzsed TCGA RNA-seq and clinical data from BRCA (n = 390) and OV (n = 228), using neoplasm cancer status as a proxy for disease-free survival (DFS). Patients were stratified by subtype-specific *WWOX/HIF1A* ratio cutpoints for exploratory Kaplan–-Meier analyses, and the ratio was also modelled as a standardizsed continuous covariate in Cox regression. Transcriptomic, pathway, immune-cell and hormone-related profiles were examined in relation to ratio status, with all multivariable and cutpoint-based findings treated as hypothesis-generating. The *WWOX/HIF1A* ratio does not act as a uniform or strongly predictive prognostic marker but instead delineates distinct biological states whose association with DFS is modest, context-dependent and statistically fragile in TCGA. Higher ratios are linked to more favourable DFS only in basal-like and HER2-enriched BRCA, together with oxidative phosphorylation, ribosomal and reduced *ADAM10*-linked Notch and monocyte/macrophage signatures. In luminal A BRCA and OV, lower ratios are linked to relatively favourable DFS and coincide with cytokine/JAK–STAT signalling, cytotoxic immune signatures and coordinated metabolic–endocrine programmes, whereas luminal B shows mixed immune and metabolic patterns. In this TCGA-based analysis, the *WWOX/HIF1A* ratio functions as a context-dependent molecular index of hypoxia, immune and hormone-related states rather than a strong, generalisable DFS predictor. The observed subtype-specific survival trends (high-ratio favourability in basal/HER2, low-ratio favourability in luminal A,B and OV) are non-significant and exploratory, and all ratio-based survival patterns require confirmation in independent cohorts and functional studies.

## 1. Introduction

Breast (BRCA) and ovarian (OV) carcinomas remain among the most lethal malignancies worldwide, in part because of pronounced tumour heterogeneity and therapeutic resistance [1,2]. Hypoxia is a central feature of cancer progression and aligns with stabilization of hypoxia-inducible factor 1 alpha (*HIF1A*), which supports metabolic reprogramming from oxidative phosphorylation towards glycolysis; the Warburg effect, even under normoxic conditions [3]. This metabolic flexibility associates with cancer cell survival and proliferation within hypoxic tumour microenvironments [4]. *WWOX*, a tumour suppressor frequently downregulated in BRCA and OV carcinomas, has been linked to an inverse relationship with *HIF1A*-associated glycolytic programmes, such that higher *WWOX* expression corresponds to attenuated pro-glycolytic gene expression and less aggressive phenotypes [5]. These observations suggest that the balance between *WWOX* and *HIF1A* may capture key aspects of hypoxia-related metabolic rewiring, tumour progression and therapy response.

Beyond metabolism, *HIF1A* expression is associated with cellular differentiation state and transcriptional changes linked to stemness and plasticity [6]. In hypoxic tumours, elevated *HIF1A* often coincides with cancer stem cell maintenance programmes and activation of signalling pathways such as Notch, which influence cell-fate decisions and lineage specification [7]. This crosstalk aligns with tumour heterogeneity and tracks with therapy resistance. Notch signalling has been implicated as a major axis connecting hypoxic responses to differentiation and immune modulation; hypoxia-driven *HIF1A* upregulation has been reported alongside increased Notch activity and *ADAM10* expression, correlating with undifferentiated, stem-like states and reduced immune surveillance [8]. The immune microenvironment is closely linked to Notch activity; tumours with prominent Notch signalling signatures often exhibit immunosuppressive features, including macrophage polarization patterns that track with immune evasion [9,10].

Within this landscape, the *WWOX/HIF1A* axis can be viewed as a molecular index reflecting the balance between metabolic reprogramming, stemness and differentiation. Attenuation of *HIF1A*-associated glycolytic shifts co-occurs with higher oxidative metabolism and differentiation-linked programmes that contrast with aggressive tumour states [11]. *WWOX* expression has been associated with higher levels of Notch-pathway inhibitors such as *DTX3* and with transcriptional signatures indicative of reduced oncogenic Notch activity [12]. Taken together, these relationships suggest that variation in the *WWOX/HIF1A* ratio may align with transitions between more plastic, hypoxia-adapted phenotypes and more differentiated states across tumour lineages.

Hormonal pathways, including oestrogen and androgen receptor signalling, further intersect with the *WWOX/HIF1A*-Notch–metabolic network [13]. Hormone receptor target genes display subtype-specific expression patterns that correlate with the *WWOX/HIF1A* ratio, particularly in luminal BRCAs where hormonal variation relates to tumour metabolism, immune microenvironment features and differentiation states [14]. These interfaces imply that hormone signalling contributes an additional regulatory layer that modulates the context in which hypoxia, Notch and *WWOX/HIF1A* operate, with potential relevance for personalised therapeutic strategies.

Despite substantial insight into these individual components, an integrated view of how *WWOX/HIF1A*-linked metabolic, immune, differentiation and hormone-related networks align with clinical outcomes remains incomplete. In particular, the prognostic relevance of the *WWOX/HIF1A* expression ratio for disease-free survival (DFS), and its associations with immune dynamics, Notch-related programmes and hormone pathways across molecular subtypes, have not been systematically examined.

Here, we investigate the *WWOX/HIF1A* ratio as an integrative molecular index within the regulatory circuitry of BRCA and OV carcinomas. By contextualising this ratio within metabolic, immune and differentiation programmes, we aim to clarify how hypoxia-related signalling interfaces with lineage-specific networks and to generate biologically grounded hypotheses that may inform future strategies targeting tumour metabolism and immune modulation.

Crucially, while our previous work established the baseline prognostic association of the *WWOX/HIF1A* ratio with overall survival (OS) [15], across TCGA breast and ovarian macro-cohorts, the present study represents a distinct extension. We shift the clinical focus to DFS, thereby emphasising early recurrence and progression dynamics, and we overlay an expanded bioinformatic framework that integrates downstream Notch-related differentiation networks, immune cell deconvolution and hormone-receptor transcriptomics with the *WWOX/HIF1A* axis. As in our OS-focused analysis, we use the same TCGA BRCA and OV carcinoma datasets, but here we reanalyse them with neoplasm cancer status as a DFS-proxy endpoint and incorporate new layers of Notch, immune and hormone-related profiling. Given the observational and bulk-sequencing nature of these data, all findings are presented as regularised, hypothesis-generating patterns that require validation in independent cohorts (e.g., METABRIC [16], GEO [17]) and in prospective functional experimental systems.

## 2. Results

### 2.1. Patient Stratification and Subtype Composition

A total of 618 patients were included and categorized into distinct molecular groups (Basal-like, Luminal A, Luminal B, HER2-enriched, and OV), then stratified into high and low *WWOX/HIF1A* ratio groups using subtype-specific optimal cutpoints (Supplementary Figure S1, Supplementary Table S7). This approach provided ratio-defined groups that capture biologically meaningful variation while maintaining comparability for downstream analyses.

Survival analysis using neoplasm cancer status as a proxy for disease-free survival (DFS) revealed context-dependent associations between *WWOX/HIF1A* ratio status and clinical outcomes. Patients with Basal-like and HER2-enriched BRCA tended to show more favourable DFS in the high-ratio group, whereas those with Luminal A, Luminal B, and OV tumours showed more favourable DFS in the low-ratio group (Supplementary Figure S1, Supplementary Table S7). Given the modest number of DFS events per subtype and the exploratory nature of the subgroup cutpoints, these effects are descriptive and hypothesis-generating rather than formally validated prognostic groupings.

To assess whether the *WWOX/HIF1A* ratio provided prognostic information beyond the individual genes, we fitted Cox models in the BRCA and OV cohorts using the ratio as a continuous covariate. In BRCA, the ratio was strongly associated with DFS (HR 0.25, 95% CI 0.15–0.44, *p* = 5.7 × 10^−7^; concordance 0.66) and remained significant in the combined model with *WWOX* and *HIF1A* (HR 0.31, 95% CI 0.14–0.70, *p* = 4.8 × 10^−3^), whereas the individual genes lost significance. In OV, the ratio was not significantly associated with DFS in the single-predictor model (HR 1.11, 95% CI 0.92–1.35, *p* = 0.27; concordance 0.49) and showed only a borderline association in the combined model (HR 1.44, 95% CI 0.99–2.09, *p* = 0.055; concordance 0.55), indicating weaker prognostic value in that cohort (Supplementary Table S6).

Taken together, these continuous-ratio Cox models suggest that the balance between *WWOX* and *HIF1A* expression carries robust prognostic value in BRCA, while the subtype-specific Kaplan–Meier analyses provide hypothesis-generating illustrations of contextual survival trends that frame our downstream transcriptional, pathway, and microenvironmental profiling.

### 2.2. Differential Expression of Notch Pathway and Hypoxia-Related Genes in Breast Carcinoma Subtypes and Ovarian Carcinoma 

The *WWOX/HIF1A* ratio showed subtype-specific associations with DFS across BRCA and OV cohorts. Differential expression analysis of curated Notch-related genes revealed distinct patterns between high and low ratio groups in different subtypes: in Luminal B BRCA: *DTX1*, *NUMBL*, *PTCRA*, and *HES6* were differentially expressed between high and low ratio groups, whereas in basal-like BRCA: *ADAM10*, *DTX3*, *EP300*, and *KAT2B* were altered, and in OV: *CTBP1*, *DLK1*, *RBPJL*, and *RFNG* were altered.

**Figure 1:**
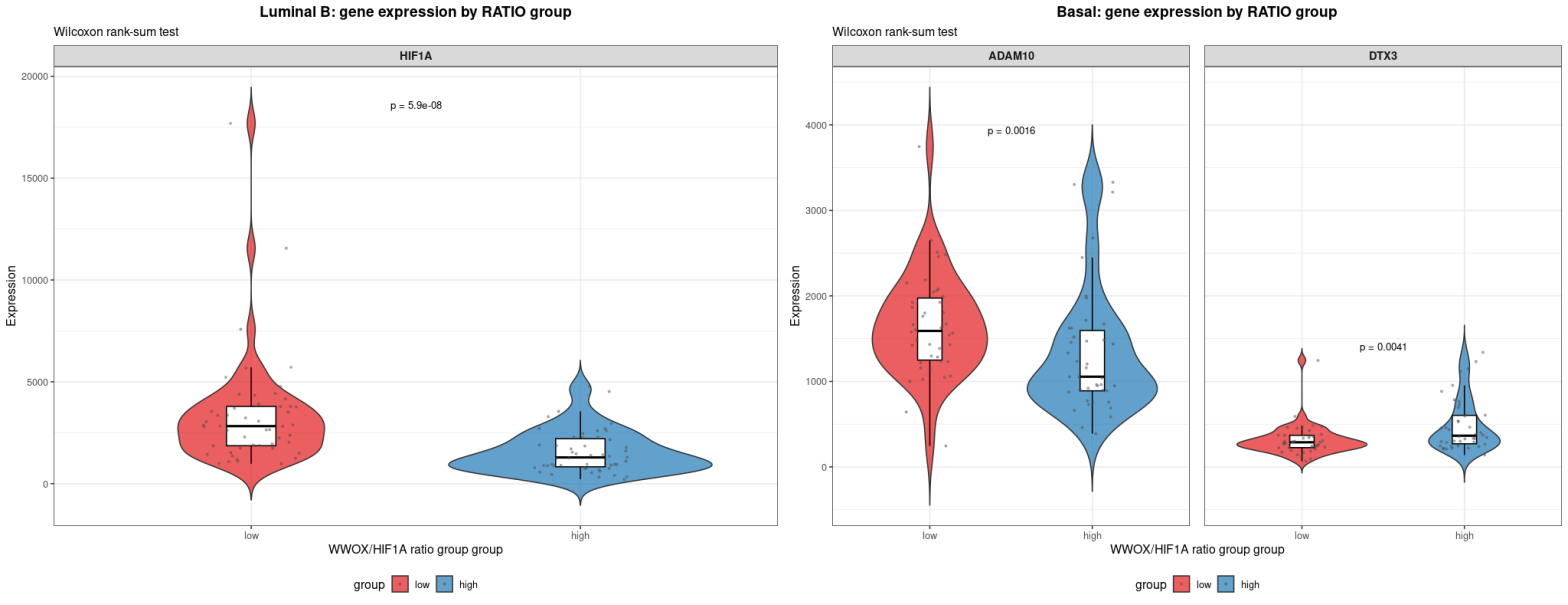
Notch pathway–associated genes *ADAM10, DTX3* and *HIF1A*. *ADAM10* was significantly higher in the low *WWOX/HIF1A* ratio group (poor DFS), whereas *DTX3* was significantly higher in the high-ratio group (better DFS prognosis) in basal-like BRCA. In Luminal B BRCA, *HIF1A* was significantly higher in the low-ratio group, which in this subtype was associated with better DFS; given that *HIF1A* contributes to the ratio definition, this difference is interpreted as a consistency check of the ratio-based stratification rather than as an independent discovery.

In Luminal B BRCA tumours, *HIF1A* expression differed markedly between *WWOX/HIF1A* ratio groups (Wilcoxon *p* = 5.9 × 10^−8^). In basal-like BRCA, the Notch pathway genes *ADAM10* and *DTX3* exhibited significant expression differences between *WWOX/HIF1A* stratified groups. *ADAM10* expression was notably higher in the low-ratio (poor DFS) group, whereas *DTX3* expression was elevated in the high-ratio group with a better prognosis (Figure 1). All reported expression differences reached statistical significance by the Wilcoxon rank-sum test (*p* ≤ 0.05). These findings underscore the divergent expression of Notch pathway members by molecular subtype and *WWOX/HIF1A* delineated disease risk, supporting subtype-specific Notch signalling associations with BRCA prognosis. Because *HIF1A* contributes directly to the ratio definition, its differential expression is expected and serves as an internal consistency check rather than an independent finding.

### 2.3. Functional Profiling of Differential Expression According to WWOX/HIF1A DFS Ratio

To synthesise the subtype-specific differential expression patterns, we examined enrichment of KEGG pathways and Hallmark gene sets according to *WWOX/HIF1A* ratio status. In hypoxia-associated, highly glycolytic settings (basal-like and HER2-enriched BRCA), higher ratios were generally associated with reduced *HIF1A*-linked glycolytic programmes, enhanced oxidative phosphorylation and ribosomal biogenesis, and more active immune-surveillance microenvironments, aligning with improved DFS. In contrast, in hormone-receptor-driven luminal A/B BRCA tumours and OV, lower ratios interfaced with distinct lineage-specific transcriptional circuitry, including cytokine and JAK–STAT signalling and coordinated metabolic–endocrine programmes, and aligned with favourable DFS. Thus, the *WWOX/HIF1A* axis does not show a uniform “high is better” or “low is worse” effect, but integrates hypoxia/metabolic, immune and differentiation pathways within each subtype’s baseline context.

#### 2.3.1. Integrated Pathway Analysis (KEGG ORA) Reveals Subtype-Specific Signatures

**Figure 2:**
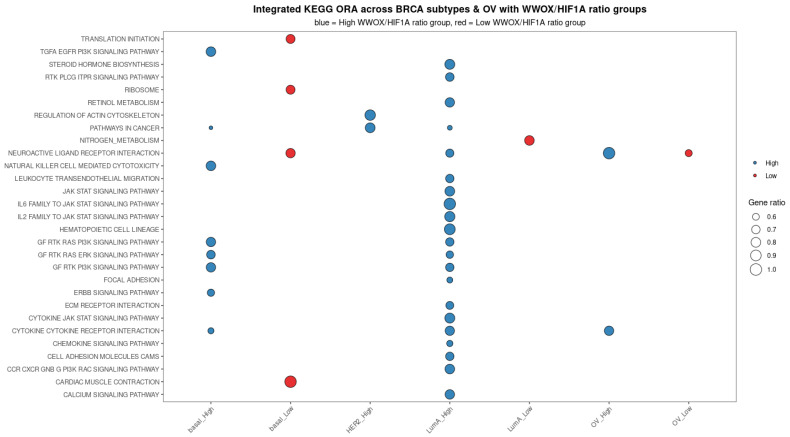
Integrated KEGG pathway over-representation analysis (ORA) across tumour subtypes and *WWOX/HIF1A* ratio groups. Integrated dot plot shows selected pathways enriched in the High (blue) or Low (red) *WWOX/HIF1A* ratio groups across BRCA subtypes and OV. Dot size represents the gene ratio (the fraction of pathway genes overlapping the differentially expressed gene set), and dot colour encodes the respective ratio group. Identical gene ratio scales are applied across all analysed groups.

To systematically evaluate functional pathways associated with the *WWOX/HIF1A* ratio, we performed KEGG over-representation analysis across BRCA subtypes and OV, restricting display to pathways enriched at FDR-adjusted *p* < 0.25 (Figure 2). 

In luminal A BRCA, high-ratio tumours were marked by broad enrichment of immune and signalling pathways, including cytokine–cytokine receptor interaction, JAK–STAT signalling, chemokine signalling, cell adhesion molecules and PI3K–Akt/Ras-related networks, whereas low-ratio tumours showed limited enrichment of nitrogen metabolism. Basal-like BRCA showed the reverse pattern, with high-ratio tumours enriched for oxidative and immune-surveillance programmes, including RTK/PI3K/Ras/ERK signalling and NK cell-mediated cytotoxicity, while low-ratio tumours were enriched for translation, ribosome and neuroactive ligand–receptor interaction pathways. 

In HER2-enriched tumours, the high-ratio group was associated with metabolic and signalling programmes, including steroid hormone biosynthesis, retinol metabolism, actin cytoskeleton regulation and general pathways in cancer. OV showed a more focused pattern, with high-ratio tumours enriched for neuroactive ligand–receptor and cytokine–cytokine receptor interaction, whereas low-ratio tumours were enriched mainly for neuroactive ligand–receptor interaction. Overall, KEGG ORA indicates that *WWOX/HIF1A* stratification captures lineage-specific shifts between immune, metabolic and signalling states.

#### 2.3.2. Hallmark GSEA Reveals a Coordinate Phenotypic Switch in Microenvironmental Programmes

**Figure 3:**
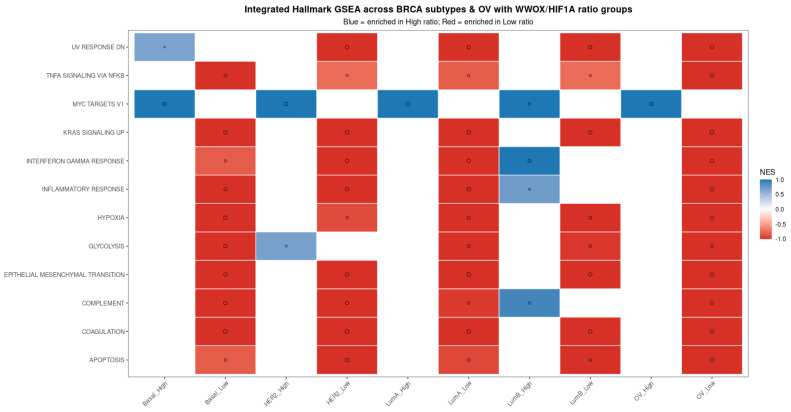
Integrated Hallmark gene-set enrichment across tumour subtypes and *WWOX/HIF1A* ratio groups. The heatmap shows Hallmark pathways with significant enrichment in at least one subtype/ratio group. The colour scale represents the normalized enrichment score (NES), with positive NES values (blue) indicating enrichment in the High *WWOX/HIF1A* ratio group and negative NES values (red) indicating enrichment in the Low-ratio group.

To summarise broader biological states, we conducted Hallmark gene-set enrichment analysis (GSEA) across *WWOX/HIF1A* ratio groups within each subtype (Figure 3).

Across subtypes, high *WWOX/HIF1A* ratios tended to align with metabolic and lineage-linked programmes, including MYC-related targets, oxidative metabolism and oestrogen-response signatures in luminal tumours, consistent with relatively organised biosynthetic and hormonal states. Conversely, low-ratio tumours more often showed enrichment of hypoxia, epithelial–mesenchymal transition (EMT), inflammatory and interferon-response pathways, together with KRAS and TNFA/NFKB-associated signalling and coagulation/complement cascades, particularly in basal-like BRCA and luminal A BRCAs. These Hallmark-level patterns are concordant with the KEGG ORA results, reinforcing the view that the *WWOX/HIF1A* ratio indexes coordinated shifts between metabolically structured and microenvironmentally stressed states in a manner that is strongly dependent on tumour lineage.

#### 2.3.3. Basal-like BRCA: Divergent Metabolic and Oncogenic Programmes

Basal-like BRCA tumours associated with better DFS and higher *WWOX/HIF1A* ratios exhibited a molecular profile characterised by enhanced metabolic and biosynthetic activity (Figure 2; Supplementary Figures S2 and S3). Pathway analysis revealed enrichment of mitochondrial bioenergetics, oxidative phosphorylation and cellular metabolism, driven by mitochondrial components such as *NDUFA11*, *NDUFA1*, *NDUFA2*, *NDUFB2* and *COX6A1*, together with metabolic regulators including *CPS1* and *DEGS2*. In parallel, ribosomal and translational pathways (*RPL/RPS/MRPL* families, *FAU*, *UBA52*) and signalling routes such as cAMP (*ADCY1*, *CAMK2B*, *GIPR*, *VIPR2*) and calcium signalling (*CACNA1F*) were enriched, along with the large Neuroactive ligand–receptor interaction gene set, consistent with elevated metabolic demand and coordinated receptor–ligand signalling.

In contrast, basal-like BRCA tumours associated with worse DFS and lower *WWOX/HIF1A* ratios displayed enrichment of pathways linked to proliferation, migration and oncogenic signalling (Figure 2; Supplementary Figure S3). Prominent genes included *EGFR*, *HGF*, *BIRC2*, *ITGAV*, *LAMA4*, *PIK3CB*, *ROCK1* and *PIP5K1B*, supporting activation of PI3K–Akt, ERBB, MAPK, mTOR, TGF-beta and other cancer-associated cascades, as well as pathways in cancer and proteoglycans in cancer. Additional enrichment of glycan, amino-acid and lipid metabolism, xenobiotic metabolism, cellular senescence and oxidative phosphorylation suggested adaptation to metabolic and oxidative stress. Hallmark analyses (Figure 3) further reinforced this pattern, with hypoxia, glycolysis, EMT, inflammatory, IL2/STAT5, KRAS, complement and coagulation signatures relatively enriched in the low-ratio group, while MYC targets were more prominent in high-ratio tumours.

#### 2.3.4. Luminal Subtypes: Hormonal Signalling and Proliferative Balance

Luminal BRCA subtypes displayed distinct but related molecular patterns reflecting their clinical behaviour. Luminal A tumours associated with better DFS and lower *WWOX/HIF1A* ratios demonstrated enrichment of hormone and immune-related pathways, whereas luminal B tumours exhibited a mixed profile combining proliferative and hormone-linked processes, consistent with an intermediate phenotype between luminal A and more aggressive subtypes.

##### Luminal A Breast Carcinoma

In luminal A BRCA with better DFS and lower *WWOX/HIF1A* ratios, enrichment analyses revealed a prominent immune phenotype, including T cell activation, immune response regulation, mononuclear cell differentiation and leukocyte cell–cell adhesion. These changes were accompanied by plasma-membrane, receptor-complex, integrin and extracellular-matrix components and by molecular functions such as immune receptor activity, cytokine and chemokine binding and MHC complex assembly (Supplementary Figure S4). KEGG analysis highlighted cytokine–cytokine receptor interaction, chemokine and JAK–STAT signalling, PI3K–Akt signalling, focal adhesion and proteoglycans in cancer, defining an immune-engaged and microenvironmentally active state (Figure 3). Collectively, these findings indicate that luminal A tumours with improved DFS and lower ratios exhibit robust immunological engagement, metabolic reprogramming and microenvironmental interactions.

Conversely, luminal A tumours with worse DFS and higher *WWOX/HIF1A* ratios were dominated by metabolic and translational programmes. KEGG analysis revealed up-regulation of core metabolic pathways (energy metabolism and biosynthesis genes such as *ADCY1*, *SLC27A5*, *NDUFA11*, *COX6B1*, *ALDH3A2*, *GLS2*, *GSTA3*, *UQCR10*, *CA2*), nitrogen metabolism (*CA13*, *CA2*, *CA7*), ribosomal genes (*RPL/RPS*), nucleotide metabolism (*TYMS*, *DTYMK*), glycosylation (*B4GALT7*, *ALG12*) and mitochondrial respiratory components (*UQCRH*, *UQCRHL*, *NDUFS6*), defining a biosynthetically active, metabolically reprogrammed luminal A subtype associated with poor outcomes (Supplementary Figure S4). 

Hallmark summaries were concordant, with MYC targets and oestrogen-response signatures enriched in the high-ratio group and hypoxia, EMT, inflammatory and interferon responses relatively enriched at lower ratios (Figure 3).

##### Luminal B Breast Carcinoma

Luminal B tumours with better DFS and lower *WWOX/HIF1A* ratios showed broad enrichment across immune, metabolic and signalling pathways. KEGG analysis identified primary immunodeficiency, T cell receptor signalling, haematopoietic cell lineage, B cell receptor signalling and cytokine–cytokine receptor interaction, with key genes including *AICDA*, *TNFRSF13C*, *CD79A*, *CD40LG*, *CD8B*, *ZAP70*, *JAK3*, *BTK* and *GRB2*, alongside IgA networks, NK-cell cytotoxicity and chemokine signalling (Supplementary Figure S6). Metabolic and detoxification pathways such as cytochrome P450 metabolism, retinol metabolism, carbohydrate digestion and pentose/glucuronate interconversions were also enriched, together with adhesion, extracellular-matrix interactions and transcriptional misregulation (e.g., *COL4A3*, *LAMC3*, *RELN*, *CNTN2*, *L1CAM*, Ras/ERBB/HIF-1 signalling), indicating preserved adaptability to proliferative and hypoxic stimuli (Figure 3 and Supplementary Figure S5).

In contrast, luminal B tumours with worse DFS and higher WWOX/HIF1A ratios were primarily associated with metabolic reprogramming. KEGG analysis showed enrichment of amino-acid metabolism (*GAD1*, *GLUL*, *PAH*), carbohydrate and lipid metabolism (*CKM*, *PTGS2*, *BLVRB*), detoxification processes (*GSTA3*, *AKR1D1*), mitochondrial function (*NDUFC2*, *NDUFA13*, *ATP12A*), methylation-associated *MAT1A* and glycosylation-related genes (*GALNTL6*, *ALG8*), defining a metabolically altered phenotype consistent with aggressive growth and therapy resistance.

Hallmark-level patterns mirrored this, with immune and xenobiotic-metabolism signatures aligning with high-ratio tumours and glycolytic/EMT-linked programmes relatively enriched at lower ratios (Figure 3).

#### 2.3.5. HER2-Enriched Subtype: Growth Factor Signalling

The HER2-enriched subtype was characterised by enrichment of pathways associated with growth-factor receptor signalling and cellular proliferation, consistent with HER2-driven oncogenesis. HER2-enriched tumours with better DFS and higher *WWOX/HIF1A* ratios showed enrichment across metabolic, endocrine and regulatory pathways, including N-glycan biosynthesis (*KRTCAP2*, *ALG2*, *OSTC*), cell-cycle regulators (*CCNB3*, *CDKN2A*, *CDKN2C*, *PTTG1*), ABC transporters (*ABCA13*, *ABCC8*) endocrine-resistance (*SHC3*, *EGFR*, *CDKN2A*, *CDKN2C*) and terpenoid backbone biosynthesis, drug metabolism by cytochrome P450 (*FMO3*, *UGT2B15*, *ADH7*) and PPAR signalling (*FABP7*, *ACSL6*, *CD36*), as well as peroxisome, insulin-secretion and fatty-acid-degradation modules (*ADH7*, *ACSL6*) (Supplementary Figure S7).

In contrast, HER2-enriched tumours with poor DFS and lower *WWOX/HIF1A* ratios were characterised by activation of oncogenic and invasion-related pathways. KEGG analysis highlighted PI3K–Akt signalling (*CSF1R*, *FGF7*, *FGFR3*, *HGF*, *PIK3CG*), pathways in cancer (*ADCY7*, *CSF1R*, *FGFR3*, *FOXO1*, *HGF*, *TGFBR2*), Ras, MAPK and Rap1 signalling, actin-cytoskeleton regulation (*FGF7*, *FGFR3*, *ITGA1*, *ITGA4*, *PAK3*, *PDGFRA*, *SSH1*) and central carbon metabolism (*FGFR3*, *PDGFRA*), together with focal adhesion (*ZEB1/2*, *FOXO1*), transcriptional misregulation (*CSF1R*, *TGFBR2*), complement and coagulation cascades (*F13A1*, *CFH*, *C1S*) (Supplementary Figure S8).

#### 2.3.6. Ovarian Carcinoma: Distinct Metabolic and Stress-Response Profiles

The OV cohort displayed a molecularly distinct signature compared with the BRCA subtypes, determined by the enrichment of pathways governing metabolic regulation, cellular homeostasis, and stress responses. Specifically, OV associated with favourable DFS and lower *WWOX/HIF1A* ratios demonstrated a coordinated upregulation of metabolic, endocrine, and regulatory networks. KEGG pathway analysis revealed distinct enrichment in neuroactive ligand–receptor interactions, pancreatic secretion, lipid and protein digestion/absorption, bile secretion, PPAR signalling (*FABP1*, *FABP5*, *ACSBG1*, *APOA2*, *APOC3*, *AQP7*, *PLIN1*, *PLIN4*), and maturity-onset diabetes of the young (Supplementary Figure S9). Together, these signatures indicate a metabolically adaptive, hormonally active, and microenvironmentally engaged phenotype that drives a superior prognosis. Mechanistically, the main contributing genes highlighted specialized cellular programmes, including lipid transport and handling (*CLPS*, *FABP1*, *APOA4*, *PLA2G1B*, *PNLIPRP1*, *DGAT2*), proteolysis and secretory activity (*CELA2A/3A/3B*, *CPA1/2*, *PRSS3*, *PGA3/4/5*), and amino acid transport alongside tissue remodelling (*MEP1A*, *COL17A1*, *SLC3A2*).

In contrast, OV tumours with worse DFS and higher *WWOX/HIF1A* ratios exhibited a stark phenotypic shift, marked by widespread enrichment of receptor-mediated cascades, metabolic stress responses (*FFAR2*), and immune-inflammatory pathways (*MAPK8*). In these high-ratio tumours, KEGG analysis highlighted neuroactive ligand–receptor interactions (*HTR1A*, *HTR1E*, *HTR6*), calcium, cAMP (*CREB1*), and cGMP–PKG signalling, serotonergic synapses, growth-hormone synthesis/secretion, AGE–RAGE signalling in diabetes (*COL4A3*, *COL4A4*, *PLCG2*, *STAT5B*), and phagosome function (*STX7*, *MRC1*, *TLR4*, *PIK3C3*, *THBS4*, *TUBAL3*) (Supplementary Figure S10).

Hallmark patterns were broadly concordant with these transcriptomic shifts: the high-ratio phenotype was driven primarily by MYC targets, whereas KRAS signalling, inflammatory and interferon responses, EMT, and coagulation cascades were preferentially enriched at lower ratios (Figure 3).

### 2.4. Subtype-Specific Differences in Immune and Stromal Cell Populations 

**Figure 4:**
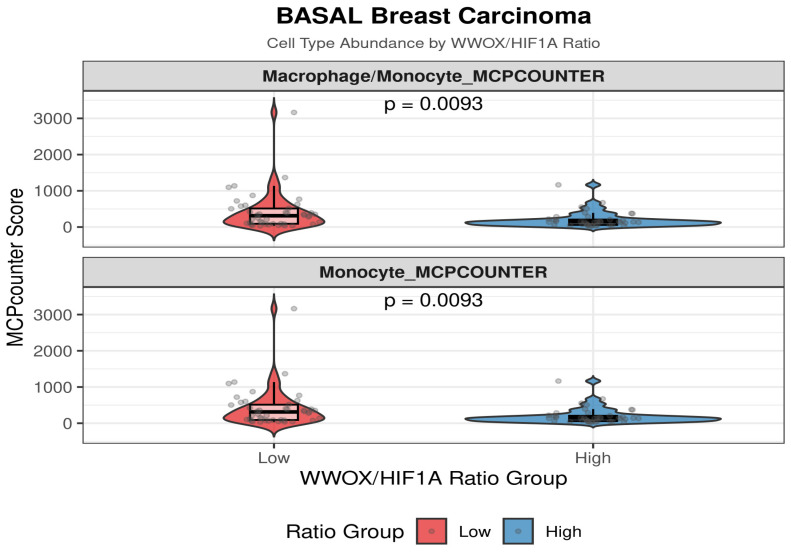
Boxplots show the MCPcounter-derived abundances for Monocyte and Macrophage/Monocyte populations in Basal-like BRCA, stratified by high and low *WWOX/HIF1A* ratio groups. Significant differences were detected using Wilcoxon rank-sum tests and are annotated where FDR < 0.05.

**Figure 5:**
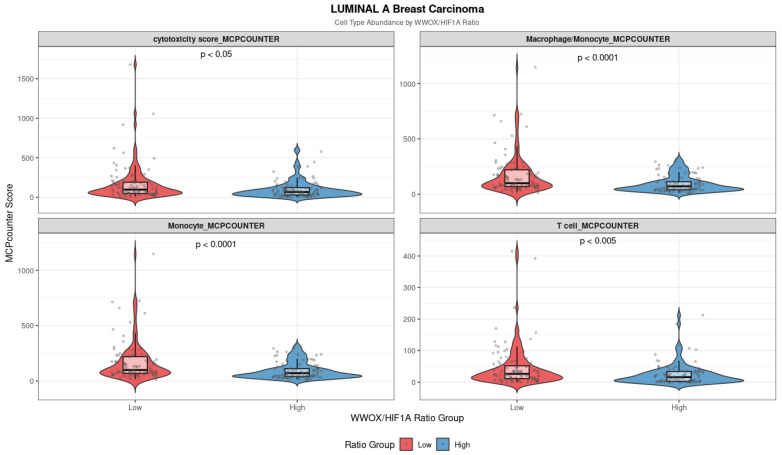
Boxplots depict the MCPcounter scores for Cytotoxicity, T cell, Monocyte, and Macrophage/Monocyte populations in Luminal A BRCA, stratified by high and low *WWOX/HIF1A* ratio groups. Significant differences were identified using Wilcoxon rank-sum tests and are annotated where FDR < 0.05.

To further investigate the tumour microenvironment associated with *WWOX/HIF1A* stratification, immune and stromal cell populations were compared across subtypes using MCPcounter scores (Supplementary Table S3; Figure 4 and Figure 5).

#### 2.4.1. Basal-like Breast Carcinoma

In basal-like BRCA, monocyte-lineage populations were significantly enriched in tumours with lower *WWOX/HIF1A* ratios. Specifically, Monocyte (*p* = 0.0093, FDR = 0.0139) and Macrophage/Monocyte scores (*p* = 0.0093, FDR = 0.0139) were elevated in the low-ratio group. These tumours were associated with worse DFS, indicating that increased infiltration of monocyte-derived cells tracks with an immunosuppressive tumour microenvironment linked to poorer clinical outcomes.

In contrast, neutrophil abundance did not differ significantly between groups (*p* = 0.1516, FDR = 0.1516), suggesting that the observed immune differences are specific to monocyte/macrophage populations rather than reflecting a non-specific global increase in immune infiltration.

#### 2.4.2. Luminal A Breast Carcinoma

In Luminal A tumours, a broader immune activation profile was observed. Tumours with lower *WWOX/HIF1A* ratios and better DFS demonstrated significantly increased T cell infiltration (*p* = 0.0034, FDR = 0.0056), cytotoxic activity (*p* = 0.0122, FDR = 0.0153), and monocyte (*p* = 0.0001, FDR = 0.0002), monocyte/macrophage populations (*p* = 0.0001, FDR = 0.0002).

This pattern suggests a more immune-infiltrated microenvironment characterised by both adaptive (T cell) and innate (monocyte/macrophage) components. The association with improved DFS indicates that, in this subtype, immune activation may reflect a more effective anti-tumour response. As in the basal-like BRCA subtype, neutrophil levels were not significantly different (*p* = 0.195, FDR = 0.195), reinforcing the specificity of the immune changes observed.

#### 2.4.3. Other Subtypes

In ovarian carcinoma, as well as HER2-enriched and Luminal B BRCA subtypes, no significant differences were observed across the evaluated immune and stromal cell populations (all FDR > 0.05). This suggests that the relationship between *WWOX/HIF1A* stratification and the tumour microenvironment is subtype-dependent, with the most pronounced associations observed in basal-like and Luminal A BRCA tumours.

### 2.5. Hormone-Related Gene Expression Across Cancer Subtypes 

Hormone-associated gene expression patterns varied across subtypes, reflecting both expected endocrine regulation in luminal tumours and alternative regulatory mechanisms in basal-like BRCA and OV (Table 1).

In basal-like BRCA, several hormone-associated genes, including *ATG16L2*, *BCAM*, *CCDC74A*, *KYNU*, *LATS2*, *PDZK1*, *PLOD2*, and *RPL13*, were differentially expressed. Most of these genes showed higher expression in the low *WWOX/HIF1A* ratio group, which was associated with worse DFS. This suggests that, in basal-like BRCA tumours, hormone-related pathways may be indirectly linked to hypoxia and metabolic stress rather than classical endocrine signalling.

In Luminal B tumours, *GAB2* was the only hormone-related gene significantly upregulated, with higher expression observed in the high-ratio group associated with worse DFS (Supplementary Figure S13). This limited signal is consistent with the more heterogeneous biology of Luminal B BRCA tumours.

In Luminal A BRCA, a broader set of hormone-associated genes, including *ABHD8*, *ARRB1*, *CDK6*, *CRISPLD2*, *DDX21*, *DOCK8*, *FLVCR2*, *LAMC1*, *LCP1*, *PLA2G16*, and *SDCBP*, were differentially expressed. Most genes showed higher expression in the low-ratio group, which in this subtype was associated with better DFS (Supplementary Figure S14). This pattern suggests that hormone-related signalling in Luminal A tumours remains functionally linked to favourable outcomes.

In ovarian carcinoma, genes including *ATP6V0A4*, *ENC1*, *GFRA1*, *HPDL*, and *WSB1* were upregulated in the low-ratio group associated with better DFS (Supplementary Figure S15), indicating distinct regulatory dynamics compared to breast cancer subtypes.

### 2.6. Multivariate Cox Regression Identifies Prognostic Gene Signatures 

#### 2.6.1. Full Breast Cancer Cohort 

In the full BRCA cohort (n = 390), we used LASSO-penalized Cox regression to regularise feature selection from an initial pool of clinical and gene-expression candidates, given the low event-to-variable ratio. Using an L_1_ penalty with a tuning parameter selected by 10-fold cross-validation, the penalized model shrank most coefficients towards zero and retained a subset of nine predictors (age, subtype, stage, *CDK6, CTBP1, DTX1, ENC1, PDZK1, RFNG*) (Supplementary Table S4). We emphasize, however, that LASSO regularization does not eliminate the fundamental statistical limitations imposed by the small number of overall events (22 events).

To obtain descriptive hazard ratios, we then fitted a multivariate Cox model including these LASSO-selected variables, recognising that post-selection estimates in this low-event setting are inherently exploratory. Advanced stage remained significantly associated with increased hazard (HR ≈ 2.78; 95% CI 1.24–6.23; *p* = 0.013), while several gene-level predictors (e.g., *CTBP1*, *CDK6*) showed weaker or borderline associations and age was not independently associated with outcome. The apparent C-index of this model was 0.79 (SE 0.055), and global tests reached significance (likelihood ratio *p* = 0.001; Wald *p* = 0.01) (Supplementary Figure S16, Supplementary Table S4), but these statistics are expected to be optimistic in view of the low event count.

Bootstrap internal validation confirmed that the multivariable breast model has negligible validated discriminative performance: the optimism-corrected C-indices were centred close to 0 with a wide interval (approximately −0.50 to 0.46). Taken together, these results indicate that the full-cohort LASSO-Cox model does not provide reliable prognostic discrimination for DFS in this setting and should be interpreted purely as hypothesis-generating.

#### 2.6.2. Subtype-Specific Analyses

Subtype-specific analyses were strictly constrained by very low DFS event counts (for example, Luminal A: n = 181). Because these cohorts lack sufficient statistical power to support robust multivariable penalised or standard Cox modelling without a high risk of overfitting, we present subtype-specific Cox results only as exploratory, hypothesis-generating descriptors of potential associations.

In Luminal A BRCA, limited exploratory Cox analysis suggested that *CDK6* and *LAMC1* may be associated with outcome. In contrast, Basal-like, HER2-enriched and Luminal B BRCA subtypes showed largely non-significant associations, which is most consistent with insufficient power rather than a definitive absence of biological relationships (Supplementary Table S5). 

#### 2.6.3. Ovarian Carcinoma Cohort 

In the ovarian carcinoma cohort (n = 228), LASSO-penalized Cox modelling identified *ATP6V0A4* as a key gene associated with outcome, with the overall model reaching statistical significance (Supplementary Table S5, Supplementary Figure S17). Other genes showed weaker, non-significant trends. Bootstrap internal validation yielded an apparent C-index of 0.55 and an optimism-corrected C-index of approximately 0.53, with a bootstrap interval of about 0.47–0.56, indicating moderate but not strong discriminative performance. This is consistent with more stable prognostic information in this higher-event setting than in the breast-only cohort, while still requiring validation in independent datasets.

## 3. Discussion

Cancer is increasingly recognized not as a single disease but as a collection of biologically distinct states shaped by dynamic interactions between tumour-intrinsic programmes and the surrounding microenvironment [18]. Among the key regulators of these processes are tumour suppressors and hypoxia-driven pathways [19], both of which independently associate with tumour progression, metabolism, and therapeutic response, yet how these systems correlate to shape tumour behaviour remains incompletely understood. In this study, we characterise the *WWOX/HIF1A* ratio as a unifying molecular feature associated with this interaction and reflecting a fundamental shift in tumour state across breast cancer subtypes and ovarian carcinoma (Table 2). Rather than acting as a simple biomarker, the *WWOX/HIF1A* ratio appears to delineate biologically distinct tumour phenotypes that integrate metabolic activity, signalling pathways, immune context, and clinical outcome. It is important to highlight that the prognostic associations described here are based on DFS, complementing and extending our prior comprehensive analysis of overall survival (OS) impact across cancer subtypes detailed in our earlier publication [15].

Sensitivity analyses comparing models with the ratio alone, *WWOX* and *HIF1A* separately, and all three terms together demonstrated that the *WWOX/HIF1A* ratio retains an independent association with DFS in the combined cohort. It effectively absorbs the small individual effects of *WWOX* and *HIF1A*, maintaining a concordance around 0.66. The ratio thus serves as a composite index summarizing the joint prognostic impact of both genes, providing integrative information beyond either gene in isolation.

A central finding of this study is the emergence of two contrasting tumour states associated with the *WWOX/HIF1A* ratio, and the prognostic meaning of those states depends strongly on subtype context. In basal-like and HER2-enriched BRCA, higher ratios are associated with better DFS and with a coordinated molecular profile characterized by enhanced metabolic and biosynthetic activity, increased translational capacity, and regulated signalling processes. Enrichment of oxidative phosphorylation and mitochondrial pathways, alongside ribosomal activity, suggests that these tumours maintain efficient energy production and protein synthesis, correlating with controlled cellular function rather than unchecked proliferation [20]. This metabolic profile contrasts with the traditional view that highly active metabolism necessarily reflects aggressive disease, instead indicating that metabolic competence and organization may be features of more stable tumour states [21]. The concurrent enrichment of neuroactive ligand–receptor interaction and signalling pathways further supports the notion of coordinated cellular communication, potentially reflecting a more differentiated or regulated phenotype. In contrast, in luminal A breast cancers and ovarian carcinomas, lower *WWOX/HIF1A* ratios are associated with more favourable DFS and with immune-permissive or metabolically coordinated states, underscoring that the prognostic direction of the axis is subtype-specific rather than uniform.

Conversely, tumours with lower *WWOX/HIF1A* ratios, associated with worse DFS in basal-like disease and other aggressive contexts, displayed a markedly different biological profile characterized by activation of oncogenic signalling pathways, metabolic stress adaptation, and increased cellular plasticity. The enrichment of PI3K–Akt, MAPK, mTOR, and ERBB signalling pathways highlights sustained proliferative and survival signalling, while the presence of hypoxia, glycolysis, and EMT signatures correlates with adaptation to adverse microenvironmental conditions and enhanced invasive potential. These findings are consistent with a tumour state closely linked to hypoxic stress and metabolic reprogramming, in which cells prioritize survival and dissemination over regulated growth [22]. The coexistence of oxidative phosphorylation with hypoxia-related pathways further suggests metabolic flexibility, a feature increasingly recognized as a hallmark of aggressive tumours.

The Notch-related findings add an important layer of biological connection to this model. Differential expression of genes such as *ADAM10*, *DTX3*, and related subtype-specific components suggests that the *WWOX/HIF1A* balance is linked not only to metabolism, but also to developmental signalling and cell-fate regulation [23,24,25]. In basal-like BRCA, low *WWOX/HIF1A* ratios were associated with *ADAM10* upregulation, a protease that activates Notch receptors and is known to correlate with EMT, invasion, and chemoresistance [26,27] and a more aggressive phenotype. By contrast, higher ratios were associated with *DTX3* expression and a more regulated molecular state, suggesting that *WWOX* may temper hypoxia-associated signalling and maintain differentiation metrics. In Luminal B tumours, *HIF1A* expression tracked with the ratio-defined groups and is interpreted primarily as a consistency check of the *WWOX/HIF1A* stratification rather than as an independent Notch-pathway discovery, whereas the additional Notch-related genes (*DTX1*, *NUMBL*, *PTCRA*, *HES6*) highlight subtype-specific wiring of this axis. This is important because it places the *WWOX/HIF1A* axis in a prominent position within a broader, interconnected hypoxia–Notch regulatory network. It also helps explain why tumours with the same nominal hypoxic balance correlate with different behaviours across subtypes: the downstream signalling architecture is distinct in every lineage.

Across subtypes, the KEGG and Hallmark analyses did not yield many pathways that survive strict FDR < 0.05 correction; instead, we highlight the most strongly enriched pathways up to FDR < 0.25 as exploratory trends, with adjusted FDR values shown directly in the dot plots. These pathway results are therefore interpreted as hypothesis-generating and require validation in independent datasets and functional studies. At the pathway level, the data show that the *WWOX/HIF1A* balance is associated with large-scale rewiring of tumour metabolism, signalling, and biosynthetic activity, but again the output is shaped by lineage context. Basal-like tumours with higher ratios showed enrichment of oxidative phosphorylation, ribosomal pathways, mitochondrial programmes, and coordinated translational activity, which together suggest a more organized and metabolically competent tumour state. This is not the same as simple “high metabolism” in the aggressive sense; rather, it points to a state in which energy production and protein synthesis remain regulated and integrated. Basal-like tumours with lower ratios, by contrast, were associated with oncogenic signalling, metabolic stress response, and immune-remodelling features. Luminal A tumours showed a different pattern, with lower ratios linked to immune-related and cytokine-associated pathways, while higher ratios were associated more strongly with biosynthetic and proliferative activity. Luminal B tumours likewise showed a mixed profile, combining immune, metabolic, and signalling pathways in a way that reflects their intermediate biology. HER2-enriched tumours were dominated by growth factor receptor signalling and cell-cycle-related programmes, consistent with their receptor-driven oncogenic context. Ovarian carcinoma showed its own distinct pattern, with metabolic, endocrine, and broad receptor–ligand signalling trends emerging as important features of the low-ratio state. Taken together, these data indicate that the same ratio can be read by different tumour lines in different ways, but the general principle remains constant: it marks the transition between organized regulatory states and stress-adapted aggressive states.

This subtype specificity becomes especially important when the immune microenvironment is considered. In basal-like BRCA, low *WWOX/HIF1A* ratios were associated with increased monocyte and macrophage infiltration, consistent with an immunosuppressive and pro-tumoural microenvironment. This is biologically plausible because hypoxia is known to correlate with the recruitment and polarization of myeloid populations that support tumour progression, immune evasion, and stromal remodelling [19]. In this setting, the ratio appears to reflect not only a tumour-intrinsic programme but also a microenvironmental state that tracks with aggressiveness. The low-ratio basal-like tumours therefore seem to be characterized by a combination of hypoxia-associated signalling and macrophage-rich immune suppression.

Luminal A tumours, however, display a different relationship. Here, lower ratios were associated with increased T cell infiltration, cytotoxic activity, and better DFS, suggesting a more immune-active and potentially immune-permissive environment. This is one of the clearest examples in the study of subtype-specific interpretation of the same molecular balance. Rather than uniformly promoting immune suppression, the *WWOX/HIF1A* ratio seems to shape the immune landscape in a manner that tracks with the transcriptional and hormonal background of the tumour. In luminal A disease, the immune signals associated with low ratios may reflect a more effective anti-tumour response, or at least a tumour context in which immune engagement is not overridden by aggressive hypoxic adaptation. This contrast with basal-like cancer underscores the importance of lineage context when interpreting tumour–immune interactions.

Taken together, these observations indicate that the *WWOX/HIF1A* ratio does not act as a uniform “higher is better” biomarker across all lineages but instead behaves as a context-dependent molecular integrator. In basal-like and HER2-enriched BRCA, higher ratios align with oxidative metabolic programmes, *DTX3*-linked Notch restraint, and reduced immunosuppressive macrophage signatures, and are associated with more favourable DFS, whereas in luminal A breast cancers and ovarian carcinomas, lower ratios coincide with cytokine-driven JAK–STAT activation, cytotoxic immune signatures, or coordinated metabolic–endocrine signalling and are associated with better DFS in those settings. These subtype-specific trajectories illustrate that the prognostic direction of the axis is determined by its integration into distinct metabolic, immune, and hormonal networks, consistent with a context-dependent integrator rather than a single, monotonic survival marker.

Hormone-related gene expression further extends this subtype-dependent framework and highlights an additional layer of biological interpretation of the *WWOX/HIF1A* axis. In basal-like tumours, differential expression of hormone-associated genes with elevated expression in the low *WWOX/HIF1A* ratio group (*ATG16L2*, *BCAM*, *CCDC74A*, *KYNU*, *LATS2*, *PDZK1*, *PLOD2*, and *RPL13*) appears to be linked primarily to stress adaptation and metabolic rewiring rather than classical endocrine signalling, consistent with the limited role of hormone pathways in this subtype. *LATS2*, a Hippo pathway component regulating proliferation and apoptosis [28]; *PDZK1*, interacting with hormone receptors and transporters [28]; and *KYNU*, a kynurenine pathway enzyme linked to immunosuppressive metabolic rewiring [29], suggest that hypoxia, metabolic stress, and hormone-related scaffolding converge in aggressive phenotypes within basal-like BRCA tumours.

In contrast, luminal A tumour exhibits a broader and more complex pattern of hormone-related gene expression, suggesting a tighter integration between endocrine signalling and the *WWOX/HIF1A* balance. Luminal A tumours with low *WWOX/HIF1A* expression ratios, associated with key genes such as *CDK6*, a cyclin-dependent kinase driving cell cycle progression, reflect hormone-driven proliferative signalling moderated in these tumours, potentially tracking with their generally favourable clinical behaviour [30]. Other genes like *ARRB1*, involved in nuclear receptor signalling modulation and G-protein coupled receptor desensitization, may contribute to nuanced regulation of hormone receptor activity and downstream transcriptional programmes [31]. *PLA2G16* and *SDCBP*, implicated in lipid metabolism and signal transduction, further highlight the metabolic integration of hormone signalling pathways essential for luminal tumour maintenance and therapy responsiveness [31,32]. The prominent expression of genes such as *CRISPLD2* and *DDX21*, involved in immune modulation and RNA helicase activity respectively, suggests that hormone pathway regulation in luminal A tumours extends beyond classic endocrine effects to encompass broader transcriptional and microenvironmental connections [33,34]. This expansive hormone-associated transcriptional plasticity in luminal A cancers underscores the complex crosstalk between hormone receptor signalling and the *WWOX/HIF1A* axis, tracking with both tumour phenotype diversity and therapeutic response, particularly to endocrine therapies.

In luminal B disease, the signal is more focused, with *GAB2* emerging as a key gene associated with the high-ratio, worse-survival state, indicating a more specific endocrine-linked regulatory correlation within this subtype [34,35]. Ovarian carcinoma again demonstrates a distinct transcriptional profile, reinforcing that the same molecular balance correlates with divergent features across different tumour types. Importantly, these findings indicate that the *WWOX/HIF1A* ratio does not simply reflect hypoxia or metabolic state but also intersects with endocrine regulatory programmes in a subtype-dependent manner. This is particularly relevant in luminal cancers, where hormone signalling is a central determinant of tumour behaviour and therapeutic response [33,34], suggesting that the biological and clinical implications of this axis may extend into endocrine treatment sensitivity.

The multivariate Cox regression results are in line with the molecular framework described above and suggest that some of the transcriptional features associated with the *WWOX/HIF1A* balance may carry prognostic information, although all such findings remain exploratory.

In the full BRCA cohort, the LASSO-derived model identified both clinical variables and gene-level predictors, including *CDK6*, *CTBP1*, *DTX1*, *ENC1*, *PDZK1*, and *RFNG*. Advanced clinical stage emerged as a strong predictor of mortality risk, while *CTBP1* and *CDK6* showed significant positive associations with hazard. These findings suggest that the molecular state captured by the *WWOX/HIF1A* balance is not merely descriptive, but tracks with clinically meaningful survival differences. Although still exploratory, these results indicate reinforced links between cell-cycle control, transcriptional repression, and hypoxia-modulated immune–metabolic states [30,36,37,38]. Although subtype-specific Cox analyses were limited by the number of events, the direction of the results still supports the broader narrative emerging from the pathway and immune data. In ovarian carcinoma, the overall model was also statistically significant, further indicating that the gene programmes associated with this axis have prognostic relevance beyond breast cancer alone.

What ties these observations together is the idea that the *WWOX/HIF1A* ratio reflects a regulatory balance between tumour suppression and hypoxia adaptation, but the downstream features of that balance are filtered through subtype-specific biology. In basal-like disease, a low-ratio state tends to align with hypoxia-driven signalling [11], macrophage-rich immune suppression [39], EMT [40], and worse outcome. In luminal A tumour, a low-ratio state is instead associated with immune engagement and better survival. In luminal B and HER2-enriched disease, the ratio captures a more mixed landscape shaped by proliferative and endocrine-related circuitry. In ovarian carcinoma, the balance correlates distinctly with metabolic, broad receptor–ligand signalling trends, and receptor-mediated pathways. These differences are not contradictions; rather, they show that the same upstream axis correlates with different biological parameters depending on cellular context, lineage history, and microenvironmental constraints.

Mechanistically, *WWOX* and *HIF1A* provide a plausible framework for this behaviour. *WWOX* is a tumour suppressor with documented roles in apoptosis, genomic stability, and metabolic regulation [4,11], whereas *HIF1A* is a core mediator of the hypoxic response, promoting glycolysis, survival, and plasticity under low oxygen conditions [41]. A shift toward lower *WWOX* and higher *HIF1A* activity would therefore be expected to correspond with hypoxia-adapted and invasive phenotypes. Our data extend this model by showing that the features accompanying this shift are not limited to metabolism alone. They also involve immune composition, Notch signalling, hormone-related transcription, and survival outcomes. This makes the *WWOX/HIF1A* ratio a more complete marker of tumour state than either gene considered individually.

From a clinical perspective, these findings suggest that the *WWOX/HIF1A* ratio may have value as an exploratory prognostic and stratification tool, but only when interpreted in the correct molecular context. Its utility will likely depend on tumour subtype, because the same directional change correlates with different biological and clinical consequences in different lineages. This also opens a therapeutic question: tumours in a low-ratio, hypoxia-adapted, immune-suppressive state may share vulnerabilities to strategies targeting hypoxia pathways, metabolic dependencies, or macrophage-driven immune evasion, whereas tumours in immune-active luminal settings may track with different interventions. The ratio therefore may be most useful not as a stand-alone biomarker, but as a guide to which biological processes dominate a given tumour.

## 4. Study Limitations and Future Directions

Several limitations should be acknowledged when interpreting these findings. First, all transcriptomic and clinical survival analyses derive exclusively from the public Cancer Genome Atlas (TCGA) repository [42] using uniformly processed Firehose data. While cross-validation and LASSO penalization support the internal stability of the *WWOX/HIF1A* ratio within this dataset, the results remain cohort-specific and exploratory. Validation in independent retrospective cohorts with annotated DFS, such as METABRIC [16] or selected GEO [17] series, is essential to confirm reproducibility across diverse clinical settings. Furthermore, the limited number of events in certain subtype-specific survival analyses reduces statistical power, rendering those specific associations strictly exploratory.

Second, the analytic framework relies on bulk tissue RNA-sequencing, which captures average expression across heterogeneous cellular populations. Provided, it cannot resolve spatial architecture, single-cell variation, or cell-type-specific effects. The identified relationships among metabolic programmes, immune microenvironments, and Notch signalling are computational and correlated rather than causal. Prospective in vitro and in vivo functional studies—including targeted perturbations of *WWOX* and *HIF1A*, manipulation of key Notch pathway components, and functional metabolic and immune assays- are required to establish direct mechanistic causality. Ultimately, these functional validations will determine whether the *WWOX/HIF1A* axis and its associated network can be developed into a clinically useful biomarker or leveraged to guide metabolic and immunotherapy-based strategies. Despite these limitations, the highly consistent patterns observed across survival, signalling, immune, hormone, and Cox analyses strengthen confidence in the overall framework.

## 5. Materials and Methods

### 5.1. Data Acquisition and Cohort Definition

Gene expression data (RNASeqV2, RSEM-normalized) and corresponding clinical profiles for patients with Breast Invasive Carcinoma (BRCA) and Ovarian Serous Cystadenocarcinoma (OV) were retrieved from The Cancer Genome Atlas (TCGA) via the Firehose Broad GDAC database (accessed 12 January 2024, http://gdac.broadinstitute.org/).

To construct the analytical cohorts, strict exclusion criteria were applied: male BRCA patients, patients with missing clinical data, and those lacking Neoplasm Cancer Status or follow-up timelines were removed. This filtering yielded initial datasets of 390 BRCA and 228 OV patients for baseline descriptive transcriptomic and pathway analyses, which were used for all the analyses (Supplementary Figure S1A).

For time-to-event survival modelling, a proxy for disease-free survival (DFS) was derived using the TCGA “Person Neoplasm Cancer Status” field (treating “With Tumour” as the event and “Tumour Free” as censoring) paired with “Days to Last Follow-up” and “Days to Death.” Patients missing any of these longitudinal parameters or key covariates were excluded as stated above, resulting in final DFS-proxy cohorts of 390 BRCA patients (22 events, 368 censored) and 228 OV patients (161 events, 67 censored), detailed in Supplementary Tables S1 and S2 and Supplementary Figure S1A. BRCA intrinsic subtypes (Basal-like, Luminal A, Luminal B, HER2-enriched) were integrated directly from published PAM50 calls and were not re-estimated.

### 5.2. Gene Expression Processing and Differential Expression

The *WWOX/HIF1A* expression ratio was computed per sample from the Level 3 RSEM-normalized expression values and utilized as a continuous variable in downstream gene and pathway-level analyses. To mitigate low-signal noise, low-expression transcripts were filtered out using the edgeR package (v3.42.4) [43], retaining only genes with counts per million (CPM) > 1 in at least 50% of samples, leaving a final dataset of 12,987 genes.

Global differential expression between ratio-stratified groups (defined via subtype-specific cutpoints; Supplementary Table S7) was assessed separately within each cohort using edgeR. Normalized count matrices were evaluated using negative-binomial generalized linear models (glmFit) and likelihood ratio tests (glmLRT) under a custom design matrix (~0 + group) using (low−high) contrasts. Differentially expressed (DE) genes were defined by an absolute log2 fold change ≥ 0.5 and a Benjamini–Hochberg (BH) false discovery rate (FDR) adjusted *p* ≤ 0.05.

### 5.3. Curated Gene Sets and Targeted Screening

A core set of 51 genes representing the Notch signalling pathway was obtained from KEGG (hsa04330) and cross-checked against the Molecular Signatures Database (MSigDB). The tumour suppressor *WWOX*, hypoxia-inducible factor *HIF1A* and the *WWOX/HIF1A* ratio were included given their established biological relevance.

To investigate hormone receptor–mediated transcription, we compiled target genes of oestrogen receptors (ESR1, ESR2) and the androgen receptor (AR) from the ChIP-X Enrichment Analysis (ChEA) [44] dataset and the ENCODE project [45]. All ESR1, ESR2 and AR target sets were merged and deduplicated to form a hormone-receptor target universe comprising (n = 5023) unique genes (Supplementary File S2), which was then intersected with edgeR-derived differentially expressed genes in each comparison to assess enrichment of hormone receptor targets among up or down-regulated genes. Cross-referencing with subtype-specific differentially expressed genes revealed distinct overlap patterns: Basal-like (n = 23), Luminal A (n = 20), Luminal B (n = 8), HER2-enriched (n = 6) and OV (n = 22) (Supplementary Table S8).

We additionally compiled a focused immune-checkpoint and T-cell exhaustion panel (*PDCD1*, *CD274*, *CTLA4*, *LAG3*, *TIGIT*, *HAVCR2*, *BTLA*, *IDO1*, *TOX*, *EOMES*, *CXCL13*) and extracted their log-CPM expression values from the edgeR-normalized RNA-seq data. Expression of these genes was examined in an exploratory manner between high and low *WWOX/HIF1A* ratio groups within each subtype using Wilcoxon rank-sum tests, and in relation to CD8 T-cell abundance and DFS in sensitivity analyses. As these checkpoint analyses did not reveal robust subtype-consistent associations beyond the main ratio-based findings, they were used primarily as internal quality control and are summarised briefly in the Results rather than presented in full detail.

### 5.4. KEGG Pathway Over-Representation Analysis (ORA)

Functional enrichment was evaluated using two complementary methodologies utilizing the global edgeR-derived DE gene lists or rankings. First, KEGG Over-Representation Analysis (ORA) was performed via hypergeometric tests against KEGG gene sets extracted from the MSigDB (v2024.1, C2 curated collection). Pathways with a Benjamini–Hochberg (BH) adjusted FDR < 0.05 were considered statistically significant. For comprehensive visualizations, exploratory trends up to an FDR ≤ 0.25 were included in bubble plots, with dot size proportional to the gene ratio and colour mapped to the enriched ratio group. Second, pre-ranked Gene Set Enrichment Analysis (GSEA) was implemented via the fgsea package (v1.26.0) [46] utilizing Hallmark gene sets (H collection) from MSigDB. Transcripts were ranked by their differential expression log_2_ fold-change, and normalized enrichment scores (NES) were calculated using 10,000 permutations under an established significance threshold of FDR < 0.25 [47].

### 5.5. Survival and Predictive Modelling

For univariate assessments, Kaplan–Meier survival curves were generated by stratifying patients into High and Low ratio groups using subtype-specific optimal cutpoints determined by maximally selected rank statistics (maxstat). To evaluate hazard ratios (HRs) without threshold-selection bias, univariable and multivariable Cox proportional hazards regression models were fitted using the standardized continuous ratio (Z-score, per SD change) as well as the cutpoint-stratified categorical ratio.

To conduct multivariable feature selection and mitigate collinearity in low-event settings, LASSO-penalized Cox proportional hazards modelling (glmnet v4.1-8) [48] was implemented. The penalized L_1_ model incorporated clinical covariates (age, stage, molecular subtype), individual gene candidates passing panel screens, and the *WWOX/HIF1A* ratio. The optimal penalty parameter (λ ≈ 0.0138) was determined via 10-fold cross-validation. Predictors retaining non-zero coefficients were subsequently evaluated in an unpenalized multivariable Cox framework to compute HRs, 95% confidence intervals (CIs), and nominal *p*-values. The proportional hazards assumption was verified via Schoenfeld residuals. 

### 5.6. Analysis of Tumour Immune Microenvironment 

To profile the tumour immune microenvironment, level 3 RSEM-normalized TCGA RNA-seq expression profiles were processed through the Estimation module on the TIMER2.0 web platform (http://timer.cistrome.org/, accessed on 10 July 2026), specifically executing TIMER2.0’s internal implementation of the MCP-counter algorithm [49,50,51]. No other deconvolution algorithms hosted on the TIMER2.0 platform (such as CIBERSORT, EPIC, or TIMER) were run or combined with these estimates. MCP-counter abundance scores were derived directly without additional batch corrections and evaluated strictly for inter-sample comparisons within individual cell populations. Statistical differences in cell population metrics between High and Low *WWOX/HIF1A* ratio groups were evaluated using two-sided Wilcoxon rank-sum tests, with statistical significance defined as FDR < 0.05.

### 5.7. Software and Computational Tools 

All analyses were conducted in R (v4.3.2) and RStudio (2024.09.0). Key R packages included: tidyverse (v2.0.0) [52] for data manipulation, survival (v3.5-7) survminer (https://rpkgs.datanovia.com/survminer/index.html, accessed on 10 July 2026) (utilising the maxstat package for cutpoint selection) for survival analyses, ggplot2 (https://ggplot2.tidyverse.org, accessed on 10 July 2026, reshape2 (https://jstatsoft.org/v21/i12, accessed on 10 July 2026) for data visualisation, dplyr (https://dplyr.tidyverse.org, accessed on 10 July 2026) and glmnet [48] for LASSO regression modelling, and msigdbr (https://igordot.github.io/msigdbr/, accessed on 10 July 2026) to provides Molecular Signatures Database (MSigDB) gene sets [24].

## 6. Conclusions

In summary, our findings show that the *WWOX/HIF1A* ratio captures a subtype-dependent shift in tumour biology that integrates metabolic state, Notch signalling, immune orientation, hormone-related transcription, and clinical outcome. Rather than functioning as a universal biomarker with a uniform meaning in every cancer type, it acts as a context-dependent molecular axis whose downstream associations are shaped by the intrinsic biology and baseline architecture of each lineage. By integrating survival, transcriptomic, pathway, immune, and hormone analyses, we provide a coherent, hypothesis-generating view of how this ratio delineates distinct, systems-level tumour states within TCGA cohorts. These observations contribute to the growing understanding of the interplay between metabolic and signalling pathways in cancer, underscoring the value of combined molecular metrics over single-gene effects. Ultimately, the *WWOX/HIF1A* balance provides a useful lens through which to master tumour heterogeneity and serves as a promising framework for future mechanistic and translational studies.

## Figures and Tables

**Table 1:** Hormone-associated genes differentially expressed between high and low *WWOX/HIF1A* ratio groups across breast cancer subtypes and ovarian carcinoma. Colour shading highlights expression directions: blue indicating significantly higher expression in the High-ratio group, and red indicating significantly higher expression in the Low-ratio group, facilitating rapid visual comparison of subtype-specific transcriptomic profiles. FDR-adjusted significance is shown where available; NS denotes genes with nominal significance but not FDR significance.

Subtype	Gene	*p*-Value	log2FC	FDR	Expression
Basal-like	*ATG16L2*	0.0008	0.5535	0.0021	Higher in High group
Basal-like	*BCAM*	0.0053	0.9740	0.0085	Higher in High group
Basal-like	*CCDC74A*	0.0406	0.3391	0.0451	Higher in High group
Basal-like	*KYNU*	0.0099	−0.8074	0.0132	Higher in Low group
Basal-like	*LATS2*	0.0451	−0.2925	0.0451	Higher in Low group
Basal-like	*PDZK1*	0.0014	1.3623	0.0027	Higher in High group
Basal-like	*PLOD2*	0.0005	−0.7978	0.0019	Higher in Low group
Basal-like	*RPL13*	0.0000	1.0155	0.0001	Higher in High group
HER2-enriched	*GAB2*	0.0072	−1.0883	0.0072	Higher in Low group
LuminalA	*ABHD8*	0.0089	0.1226	0.0098	Higher in High group
LuminalA	*ARRB1*	0.0013	0.3184	0.0018	Higher in Low group
LuminalA	*CDK6*	0.0000	−0.6830	0.0000	Higher in Low group
LuminalA	*CRISPLD2*	0.0006	−0.4293	0.0010	Higher in Low group
LuminalA	*DDX21*	0.0137	−0.1462	0.0137	Higher in Low group
LuminalA	*DOCK8*	0.0000	−0.5200	0.0000	Higher in Low group
LuminalA	*FLVCR2*	0.0000	−0.4405	0.0000	Higher in Low group
LuminalA	*LAMC1*	0.0002	−0.4015	0.0004	Higher in Low group
LuminalA	*LCP1*	0.0000	−0.8111	0.0000	Higher in High group
LuminalA	*PLA2G16*	0.0034	0.4929	0.0042	Higher in Low group
LuminalA	*SDCBP*	0.0000	−0.2973	0.0000	Higher in Low group
Ovarian	*ATP6V0A4*	0.0193	−0.8558	0.0193	Higher in Low group
Ovarian	*ENC1*	0.0083	−0.4318	0.0104	Higher in Low group
Ovarian	*GFRA1*	0.0061	−1.4522	0.0101	Higher in Low group
Ovarian	*HPDL*	0.0002	0.5834	0.0011	Higher in High group
Ovarian	*WSB1*	0.0035	−0.2156	0.0087	Higher in Low group

**Table 2:** Summary of molecular, metabolic, immune, Notch, and hormone signalling characteristics stratified by *WWOX/HIF1A* expression ratio groups across BRCA and OV subtypes.

Cancer Subtype	*WWOX/HIF1A* Ratio Group	Key Enriched Pathways	Metabolic Profile	Immune Landscape	Hormone Signalling
Basal-like BRCA	High	Oxidative phosphorylation; Ribosome; Mitochondrial bioenergetics; Neuroactive ligand-receptor interaction	Enhanced oxidative metabolism; Active mitochondrial function; Biosynthetic activity	Moderate immune activity	*PDZK1*; Hippo pathway involvement
Basal-like BRCA	Low	PI3K-Akt signalling; MAPK signalling; Central carbon metabolism; Proteoglycans in cancer; mTOR signalling	Glycolytic shift; Oncogenic metabolism; Metabolic flexibility for proliferation	Immunosuppressive; Increased monocyte/macrophage infiltration (*p* = 0.0093)	*KYNU*, *PLOD2*, *LATS2* Immunosuppressive kynurenine pathway
Luminal A BRCA	Low	Cytokine-cytokine receptor interaction; JAK-STAT signalling; Chemokine signalling; T cell receptor signalling	Immune-supportive metabolism; Cytokine production; Antigen presentation	Immunologically active; Enhanced T cell activity (*p* = 0.0034); High cytotoxicity (*p* = 0.0122)	*CDK6*, *LAMC1*, Broad hormonal regulation
Luminal A BRCA	High	Metabolic pathways; Ribosome; Nitrogen metabolism; translation programme	Hyperactive biosynthesis; Increased translation; Metabolic reprogramming	Limited immune engagement; Metabolically focused	*PLA2G16*, Limited hormone pathway activity
Luminal B BRCA	Low	Primary immunodeficiency; T/B cell receptor signalling; Cytokine interaction; Metabolic flexibility pathways	Immune activation metabolism; Detoxification; Metabolic adaptability	Robust immune activation; Enhanced adaptive immunity; Preserved immune function	Enhanced hormone receptor interactions
Luminal B BRCA	High	Metabolic reprogramming; Amino acid metabolism; Mitochondrial function; Glycosylation pathways	Altered amino acid metabolism; Dysregulated biosynthesis; Mitochondrial dysfunction	Immune suppression; Reduced immune surveillance	*GAB2*; Oncogenic hormone signalling
HER2-Enriched BRCA	High	N-glycan biosynthesis; Cell cycle regulation; Drug metabolism; PPAR signalling; Terpenoid biosynthesis	Regulated cell cycle metabolism; Drug metabolism; Lipid metabolism	Balanced immune environment; Controlled activation	Endocrine resistance pathways; Hormone metabolism
HER2-Enriched BRCA	Low	PI3K-Akt signalling; MAPK signalling; Focal adhesion; EGFR resistance; Epithelial–mesenchymal transition	Growth factor-driven metabolism; Invasion-supportive metabolism; Resistance mechanisms	Pro-inflammatory; Invasion-promoting immune environment	Hormone receptor dysfunction; Resistance mechanisms
Ovarian carcinoma	Low	Neuroactive ligand-receptor interaction; PPAR signalling; Pancreatic secretion; Complement cascades	Adaptive metabolism; Endocrine regulation; Homeostatic maintenance	Immune homeostasis; Controlled inflammatory response	*ATP6V0A4* (protective); *HPDL*; Metabolic homeostasis
Ovarian carcinoma	High	Calcium/cAMP signalling; Serotonergic synapse; AGE-RAGE signalling; Growth hormone synthesis	Dysregulated receptor–mediated signalling metabolism; Inflammatory metabolism; Stress responses	Immune dysfunction; Aberrant inflammatory signalling	*WSB1*, *ENC1*, *GFRA1*; Disrupted endocrine function

## Data Availability

TCGA RNA-seq data for breast cancer (BRCA) and Ovarian Carcinoma (OV) are publicly available from (http://gdac.broadinstitute.org/, accessed on 10 July 2026).

## Supplementary Materials

The following supporting information can be downloaded at: https://www.mdpi.com/article/10.3390/ijms27156740/s1.

## Abbreviations

Abbreviation	Definition
ADCY1/7/8	Adenylate cyclase 1/7/8 (cAMP signalling enzymes)
ADAM10	A disintegrin and metalloproteinase domain-containing protein 10
AR	Androgen receptor
ATG16L2	Autophagy-related 16-like 2
ATP6V0A4	V-type proton ATPase subunit A4
BCAM	Basal cell adhesion molecule
BH	Benjamini–Hochberg
BRCA	Breast invasive carcinoma (TCGA breast cancer cohort)
BTK	Bruton’s tyrosine kinase
CDK6	Cyclin-dependent kinase 6
CDKN2A/CDKN2C	Cyclin-dependent kinase inhibitor 2A/2C
CGA	Glycoprotein hormones alpha subunit
CI	Confidence interval
CPM	Counts per million
CTBP1	C-terminal binding protein 1
CTLA4	Cytotoxic T-lymphocyte-associated protein 4
DE	Differentially expressed genes
DFS	Disease-free survival
DTX1/DTX3	Deltex E3 ubiquitin ligase 1/3
EGFR	Epidermal growth factor receptor
EMT	Epithelial–mesenchymal transition
ENCODE	Encyclopedia of DNA Elements project
ESR1/ESR2	Oestrogen receptor 1/2
FDR	False discovery rate (Benjamini–Hochberg adjusted *p*-value)
FGSEA	Fast gene set enrichment analysis (R package)
GAB2	GRB2-associated binding protein 2
GEO	Gene Expression Omnibus
GIPR	Gastric inhibitory polypeptide receptor
GO	Gene Ontology
HIF1A	Hypoxia-inducible factor 1 alpha
HR	Hazard ratio
HRH3	Histamine receptor H3
JAK–STAT	Janus kinase–signal transducer and activator of transcription
KEGG	Kyoto Encyclopedia of Genes and Genomes
KYNU	Kynureninase
LAMC1	Laminin subunit gamma 1
LASSO	Least absolute shrinkage and selection operator
LATS2	Large tumour suppressor kinase 2 (Hippo pathway component)
LCP1	Lymphocyte cytosolic protein 1
MCPcounter	Microenvironment Cell Populations counter
METABRIC	Molecular Taxonomy of Breast Cancer International Consortium
MSigDB	Molecular Signatures Database
NES	Normalised enrichment score (GSEA)
NDUFA/B/F/S	NADH oxidoreductase subunits (mitochondrial complex I)
NOTCH	Notch signalling pathway
OV	Ovarian serous cystadenocarcinoma (TCGA OV cohort)
OS	Overall survival
PAM50	50-gene intrinsic molecular subtype classifier for breast cancer
PDZK1	PDZ domain-containing 1
PI3K–Akt	Phosphoinositide 3-kinase–Akt signalling pathway
PPAR	Peroxisome proliferator-activated receptor
PLOD2	Procollagen-lysine, 2-oxoglutarate 5-dioxygenase 2
qPCR	Quantitative polymerase chain reaction
RPL/RPS/MRPL	Ribosomal protein large/small/mitochondrial ribosomal large subunits
RSEM	RNA-Seq by Expectation-Maximisation (normalisation method)
TCGA	The Cancer Genome Atlas
TIMER2.0	Tumour Immune Estimation Resource, version 2.0
TNFRSF13C	Tumour necrosis factor receptor superfamily member 13C
TYMS/DTYMK	Thymidylate synthase/deoxythymidylate kinase
WWOX	WW domain-containing oxidoreductase (tumour suppressor)
*WWOX/HIF1A* ratio	Expression ratio between WWOX and HIF1A used for stratification
