# Supplementary material for: WWOX/HIF1A Balance Delineates Context-Dependent Molecular States in Breast Cancer Subtypes and Ovarian Carcinoma
Source: Int J Mol Sci. 2026 Jul 28;27(15):6740. doi: 10.3390/ijms27156740 (PMC13467099; doi:10.3390/ijms27156740)
Supplement: Supplementary file 1 [file ijms-27-06740-s001.zip › ijms-4365276-Supplementary File S1.pdf]

WWOX/HIF1A Balance Delineates Context-Dependent Molecular States in Breast Cancer Subtypes and Ovarian Carcinoma

Raneem Y. Hammouz *, Kinga Maciejek and Andrzej K. Bednarek

Department of Molecular Carcinogenesis, Medical University of Lodz, Żeligowskiego 7/9, 90-752 Lodz, Poland; kinga.maciejek@umed.lodz.pl (K.M.); andrzej.bednarek@umed.lodz.pl (A.K.B.)
* Correspondence: raneem.hamouz@umed.lodz.pl

Supplementary Table S1. Breast cancer(BRCA) patient clinical data. Stage I–IV according to AJCC 7th edition. LMP: last menstrual period.

**NUMBER OF PATIENTS**	433	
**RACE**	WHITE	302
	BLACK OR AFRICAN AMERICAN	32
	AMERICAN INDIAN OR ALASKA NATIVE	1
	NOT AVAILABLE	68
	ASIAN	30
**VITAL STATUS**	ALIVE	385
	DEAD	48
**PERSON NEOPLASM CANCER STATUS**	NOT AVAILABLE	43
	WITH Tumour	22
	Tumour FREE	368
**HISTOLOGICAL SUBTYPE**	BASAL-LIKE-LIKE	85
	HER2-ENRICHED-ENRICHED	47
	LUMINAL A-LIKE	199
	LUMINAL B-LIKE	102
**PATHOLOGIC STAGE**	STAGE I	81
	STAGE II	252
	STAGE III	89
	STAGE IV	1
	STAGE X	6
	NOT AVAILABLE	4
**MENOPAUSE STATUS**	Pre (<6 months since LMP AND no prior bilateral ovariectomy AND not on estrogen replacement)	105
	Peri (6-12 months since last menstrual period)	13
	Post (prior bilateral ovariectomy OR >12	278

	months since LMP with no prior hysterectomy)	
	Indeterminate (neither Pre or Postmenopausal)	0
	NOT AVAILABLE	37
**AGE AT INITIAL PATHOLOGIC DIAGNOSIS**	< 39	38
	40 - 50	94
	51 - 65	177
	66 - 100	124
**ESTROGEN RECEPTOR STATUS**	POSITIVE	330
	NEGATIVE	99
	Performed but Not Available or Not Available	3
	INDETERMINATE	1
**PROGESTERON RECEPTOR STATUS**	POSITIVE	282
	NEGATIVE	146
	Performed but Not Available or Not Available	3
	INDETERMINATE	2
**HER2-ENRICHED RECEPTOR STATUS**	POSITIVE	64
	NEGATIVE	354
	NOT AVAILABLE	8
	EQUIVOCAL	7

Supplementary Table S2. Ovarian cancer(OV) patient clinical data. Stage I–IV according to AJCC 7th edition.

**NUMBER OF PATIENTS**	249	
**RACE**	WHITE	205
	BLACK OR AFRICAN AMERICAN	22
	AMERICAN INDIAN OR ALASKA NATIVE	1
	NOT AVAILABLE	11
	ASIAN	10
**VITAL STATUS**	ALIVE	116
	DEAD	133
**PERSON NEOPLASM CANCER STATUS**	NOT AVAILABLE	21
	WITH Tumour	161
	Tumour FREE	67
**HISTOLOGICAL TYPE**	Cystadenocarcinoma, NOS	0
	Papillary carcinoma, NOS	0
	Papillary serous cystadenocarcinoma	1

	Serous cystadenocarcinoma, NOS	235
	NOT AVAILABLE/UNKNOWN	13
**PATHOLOGIC STAGE**	STAGE I	0
	STAGE II	17
	STAGE III	152
	STAGE IV	21
	NOT AVAILABLE	59
**AGE AT INITIAL PATHOLOGIC DIAGNOSIS**	< 39	6
	40 - 50	59
	51 - 65	120
	66 - 100	64

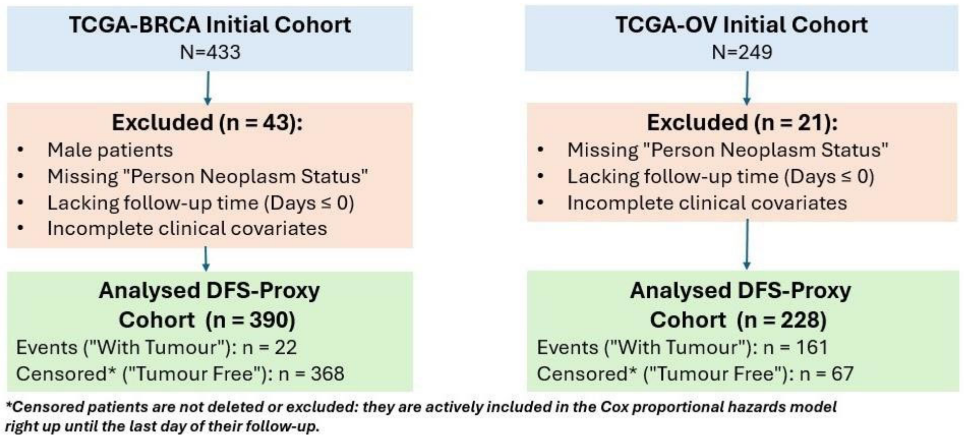

Supplementary Figure S1A. Cohort Selection and Exclusion Flowchart. Step-by-step sample filtering for TCGA-BRCA (n = 433) and TCGA-OV (n = 249) datasets down to the final analyzed DFS-proxy cohorts (n= 390 and n = 228, respectively). The same cohort was used throughout the analyses.

Supplementary Table S3. Comparison of Immune and Stromal Cell Populations Across Breast and Ovarian Cancer Subtypes. Summary of immune and stromal cell population differences by cancer subtype and *WWOX*/*HIF1A* ratio groups. The table lists breast and ovarian cancer subtypes with corresponding immune cell types analyzed using MCPcounter scores. Statistical significance of differences between low and high *WWOX*/*HIF1A* ratio groups was assessed by a Wilcoxon rank-sum test with false discovery rate (FDR) correction. P-values and FDR values are shown, with significance defined as FDR < 0.05. The direction of difference indicates whether cell abundance is higher in the low or high ratio group. Abbreviations: ns: not significant, BRCA: breast cancer.

Subtype	Cell Type	p-value	FDR	Significant (FDR < 0.05)	Direction (Low vs High)
Basal-like BRCA	Monocytes	0.0093	0.0139	Yes	Low > High
Basal-like BRCA	Macrophages/Monocytes	0.0093	0.0139	Yes	Low > High
Basal-like BRCA	Neutrophils	0.1516	0.1516	No	ns
Luminal A BRCA	T cells	0.0034	0.0056	Yes	Low > High
Luminal A BRCA	Cytotoxicity score	0.0122	0.0153	Yes	Low > High
Luminal A BRCA	Monocytes	0.0001	0.0002	Yes	Low > High
Luminal A BRCA	Macrophages/Monocytes	0.0001	0.0002	Yes	Low > High
Luminal A BRCA	Neutrophils	0.1950	0.1950	No	ns
Ovarian	Neutrophils	0.3088	0.4118	No	ns
Ovarian	Endothelial cells	0.0188	0.0751	No	ns
HER2-ENRICHED BRCA	Monocytes	0.8400	0.9876	No	ns
HER2-ENRICHED BRCA	Macrophages/Monocytes	0.8400	0.9876	No	ns
HER2-ENRICHED BRCA	Cancer-associated fibroblasts	0.9876	0.9876	No	ns
Luminal B BRCA	T cells	0.4406	0.5875	No	ns
Luminal B BRCA	CD8+ T cells	0.2272	0.4543	No	ns
Luminal B BRCA	B cells	0.7106	0.7106	No	ns
Luminal B BRCA	Neutrophils	0.1263	0.4543	No	ns

Supplementary Table S4. Exploratory multivariable Cox model for the full BRCA cohort based on LASSO-selected variables (N total= 390; 22 events, 368 censored). HR, hazard ratio; CI, confidence interval; p < 0.05*, p < 0.005***.

Variable	N(Total)	Hazard Ratio (HR)	95% CI	p-value	Notes
age	390	0.98	0.94 – 1.02	0.419	
type	390	1.56	0.97 – 2.50	0.069	Borderline
stage	390	2.78	1.24 – 6.23	0.013 *	Significant
CDK6	390	1.00	1.00 – 1.00	0.033 *	Significant
CTBP1	390	1.00	1.00 – 1.00	<0.001 ***	Highly significant
DTX1	390	0.99	0.98 – 1.00	0.064	Borderline
ENC1	390	1.00	1.00 – 1.00	0.068	Borderline
PDZK1	390	1.00	1.00 – 1.00	0.174	
RFNG	390	1.00	1.00 – 1.00	0.112	

Supplementary Table S5. Exploratory multivariable Cox model for the OV cohort based on LASSO-selected variables (N total = 228; 161 events, 67 censored). HR, hazard ratio; CI, confidence interval; p < 0.05.*

Variable	N(total)	Hazard Ratio (HR)	95% CI	p-value	Notes
age	228	1.01	0.99 – 1.01	0.308	
stage	228	1.03	0.66 – 1.60	0.893	
ADAM10	228	1.00	1.00 – 1.00	0.370	
ATP6V0A4	228	0.99	0.99 – 1.00	0.041 *	Significant
CCDC74A	228	1.00	1.00 – 1.00	0.522	
CDK6	228	1.00	1.00 – 1.00	0.082	Borderline
CRISPLD2	228	1.00	1.00 – 1.00	0.504	
DDX21	228	1.00	1.00 – 1.00	0.071	Borderline
DTX1	228	1.00	1.00 – 1.00	0.054	Borderline
EP300	228	1.00	1.00 – 1.00	0.252	
HPDL	228	1.00	1.00 – 1.00	0.324	
LCP1	228	1.00	1.00 – 1.00	0.562	
PDZK1	228	1.00	1.00 – 1.00	0.401	
SDCBP	228	1.00	1.00 – 1.00	0.265	

Supplementary Table S6. Sensitivity analysis of the *WWOX*/*HIF1A* ratio versus *WWOX* and *HIF1A* individually in the TCGA multi-cancer survival-proxy cohort (n = 618; 183 events). Sensitivity analysis of the *WWOX*/*HIF1A* ratio versus *WWOX* and *HIF1A* individually in TCGA breast and ovarian cancer DFS-proxy cohorts. HR, hazard ratio; CI, confidence interval; DFS, disease-free survival. The *WWOX*/*HIF1A* ratio was analyzed as a standardized continuous covariate (per standard deviation increase).

Cohort	Covariates	Variable	HR (95% CI)	p-value	Concordance
**Breast**	**Ratio only (per SD)**	Ratio	0.25 (0.15–0.44)	5.7×10^{-7}	0.66
	WWOX* + *HIF1A	*WWOX*	0.9982 (0.9972–0.9992)	2.6×10^{-4}	
		HIF1A	1.0001 (1.0000–1.0002)	1.7×10^{-4}	0.65
	***WWOX* + *HIF1A* + ratio (per SD)**	*WWOX*	0.9998 (0.9985–1.0011)	0.74	
		HIF1A	1.0000 (1.0000–1.0001)	0.43	
		Ratio	0.31 (0.14–0.70)	4.8×10^{-3}	0.66

**Ovarian**	**Ratio only (per SD)**	Ratio	1.11 (0.92–1.35)	0.27	0.49
	WWOX* + *HIF1A	*WWOX*	≈1.00 (n.s.)	n.s.	
		HIF1A	≈1.00 (n.s.)	n.s.	0.52
	***WWOX* + *HIF1A* + ratio (per SD)**	*WWOX*	≈1.00 (n.s.)	n.s.	
		HIF1A	≈1.00 (n.s.)	n.s.	
		Ratio	1.44 (0.99–2.09)	0.055	0.55

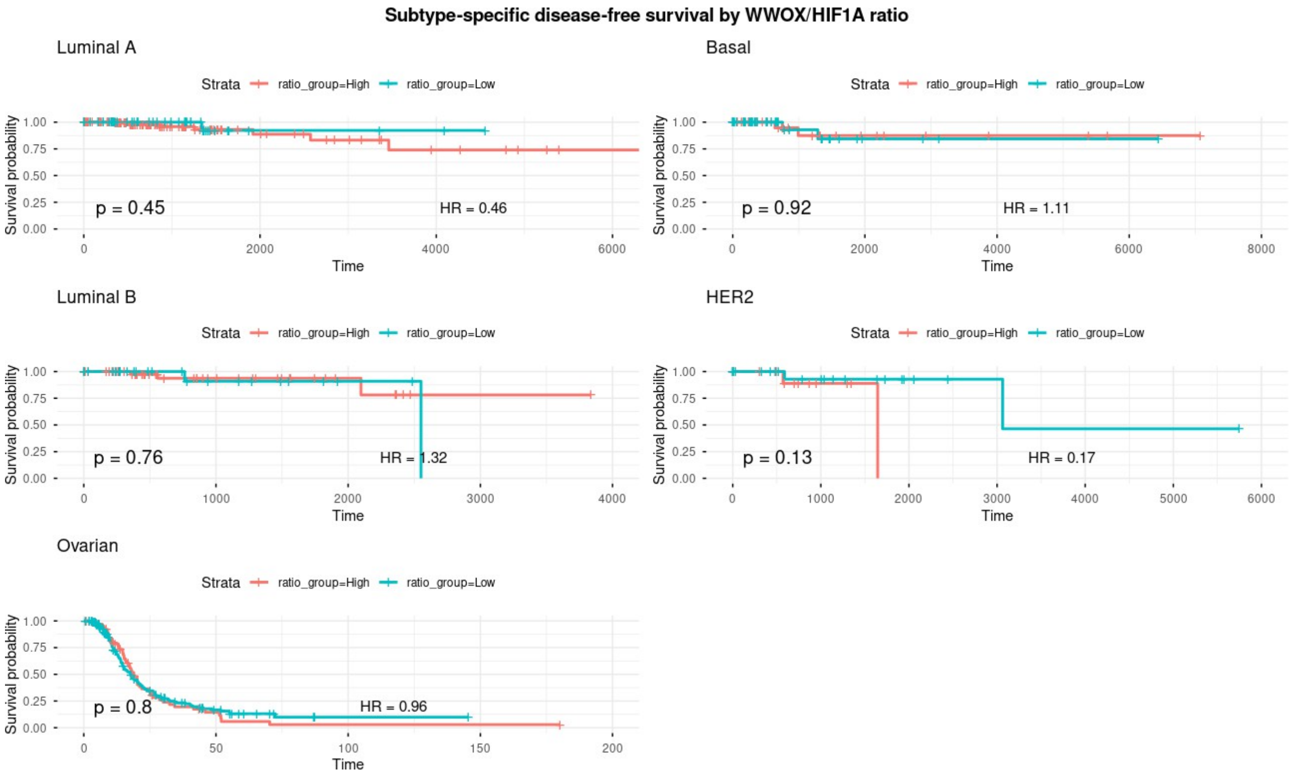

Supplementary Figure S1. Kaplan–Meier curves for disease-free survival (DFS) across BRCA subtypes and OV according to *WWOX/HIF1A* ratio. High and Low groups were defined using subtype-specific optimal cutpoints determined by maximally selected rank statistics, as described in the methodology. These analyses are exploratory and intended to provide descriptive context.

Supplementary Table S7. Subtype-specific *WWOX/HIF1A* ratio cutpoints and disease-free survival (DFS) associations. Patients were stratified into High and Low *WWOX/HIF1A* ratio groups using subtype-specific optimal cutpoints derived from maximally selected rank statistics (maxstat). The table reports the cutpoint value, hazard ratio (HR) for DFS (High vs Low), corresponding p-value, and the group with relatively favourable DFS. Given the data-driven nature of cutpoint selection, these results are considered exploratory and hypothesis-generating rather than confirmatory, and none reached conventional statistical significance.

subtype	Cutpoint value	HR	p-value	more favourable DFS group
**basal-like**	0.08865	1.11	0.92	high
**HER2-enriched**	0.1345	0.17	0.13	high
**Lum A**	0.1689	0.46	0.45	low
**RLum B**	0.07937	1.32	0.76	low
**OV**	0.05263	0.96	0.8	low

Supplementary Table S8. Subtype-specific intersection of differentially expressed genes with hormone receptor transcriptional target networks. Summary of differentially expressed genes (DEGs) identified across BRCA molecular subtypes and OV that overlap with the consolidated *ESR1*, *ESR2*, and *AR* transcription factor target universe (n = 5,023 unique genes compiled from ChEA and ENCODE ChIP-seq databases). DEs were defined using edgeR under a threshold of log2FC ≥ 0.5 and FDR ≤ 0.05. Detailed gene lists for each subtype overlap are provided in Supplementary File S2.

**Basal-like**	**Her2-enriched**	**Luminal A**	**Luminal B**	**Ovarian**
PDZK1	*PITPNC1*	*CDK6*	*PLOD2*	*TDRD1*
TFF1	*ADORA1*	*DOCK8*	*CALCR*	*DHRS2*
STC2	*ATXN1*	*CYP1A1*	*SQSTM1*	*GFRA1*
SLC16A6	*BACE1*	*BMP7*	*MYPN*	*PPAP2B*
RHPN2	*ENC1*	*CHPT1*	*CLDN9*	*RET*
SEC14L2	*GAB2*	*CRISPLD2*	*GAB2*	*DIO1*
BCAM		*ABHD8*	*GPR37L1*	*PLCG2*
ABAT		*PLA2G16*	*SLC16A6*	*WSB1*
SEMA3B		*DDX21*		*WISP2*
LMCD1		*LCP1*		*GRAMD1A*
LATS2		*FLVCR2*		*MYPN*
ATG16L2		*ARRB1*		*CALCR*
CCDC74A		*SEC14L2*		*ZNF365*
RPL13		*GRB10*		*TFF1*
KYNU		*KRT15*		*ATP6V0A4*
PLOD2		*SDCBP*		*HPDL*
MCCC2		*LAMC1*		*IER2*

MYEOV		*GPRC5A*		*CHPT1*	
DHRS2		*PACSIN1*		*IRS2*	
PRKRIP1		*DSCAM*		*ENC1*	
NT5C2				*JPH3*	
RET				*TPD52L1*	
NOP16					

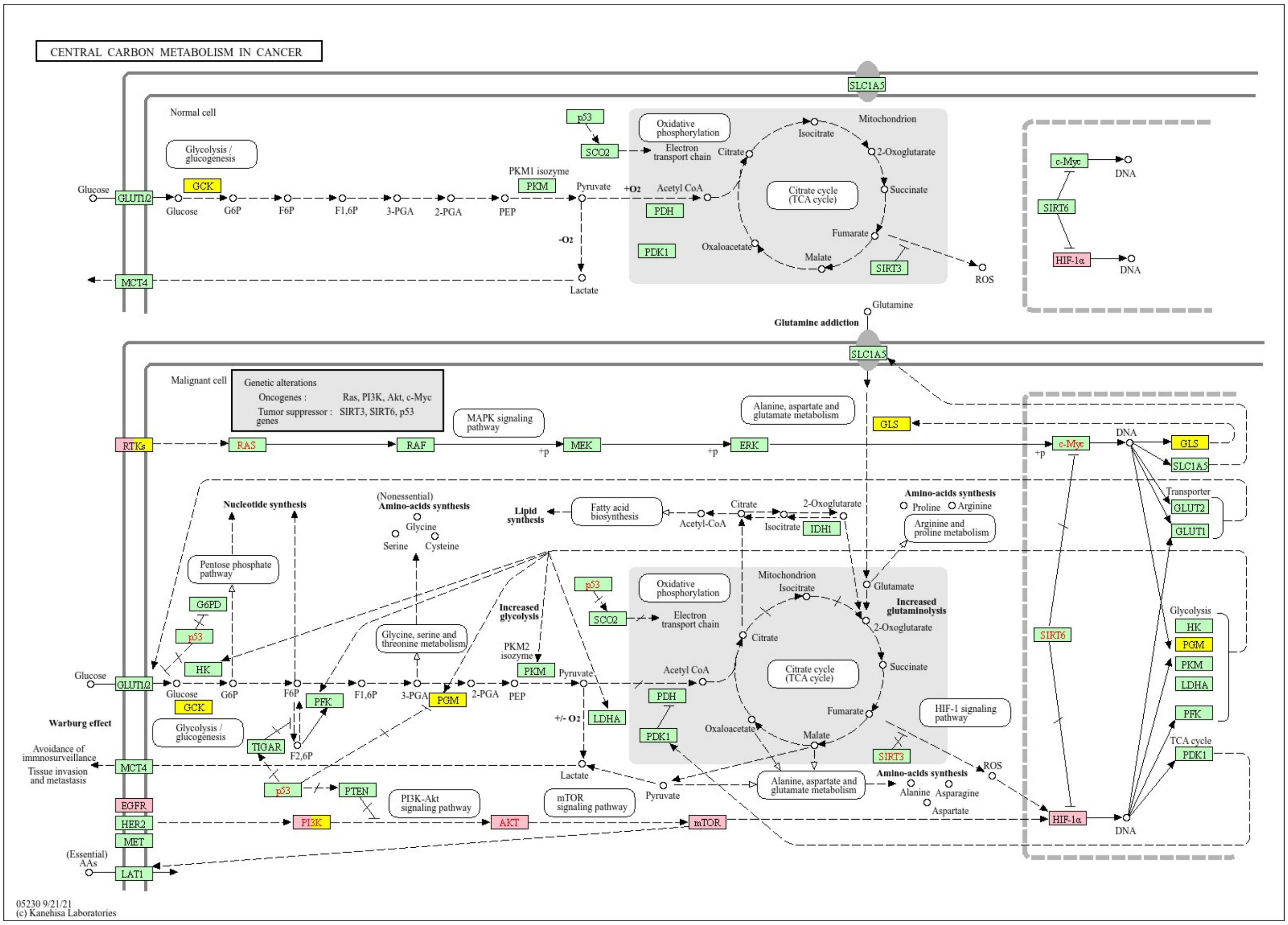

Supplementary Figure S2. Metabolism differentially expressed pathways in basal-like BRCA stratified by *WWOX/HIF1A* ratio. Key signalling pathways vary significantly with prognosis. Yellow indicates good prognosis, pink signifies poor prognosis, and green indicates genes with unchanged expression.

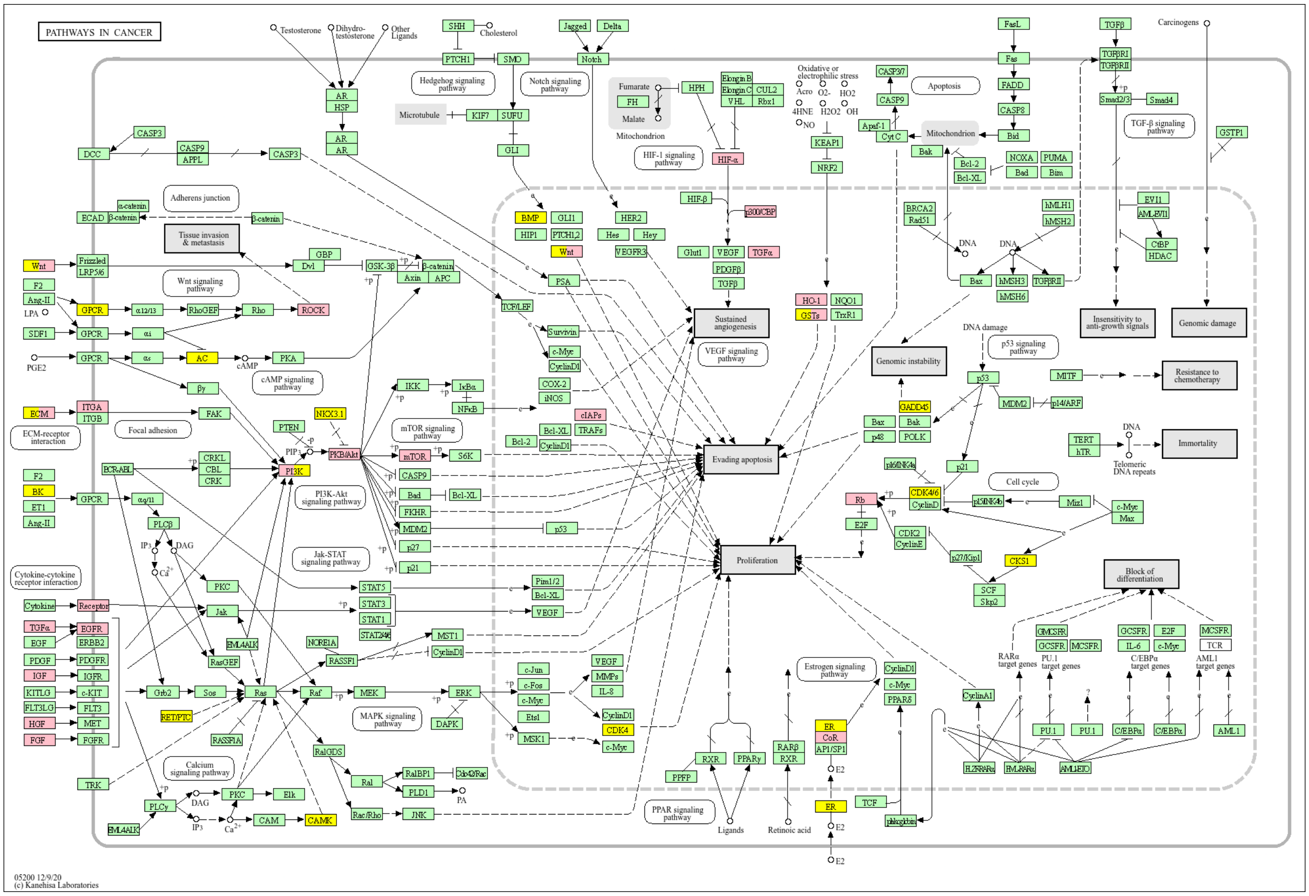

Supplementary Figure S3. Differentially expressed pathways in basal-like BRCA stratified by *WWOX*/*HIF1A* ratio. Key signalling pathways vary significantly with prognosis. Yellow indicates good prognosis, pink signifies poor prognosis, and green indicates genes with unchanged expression.

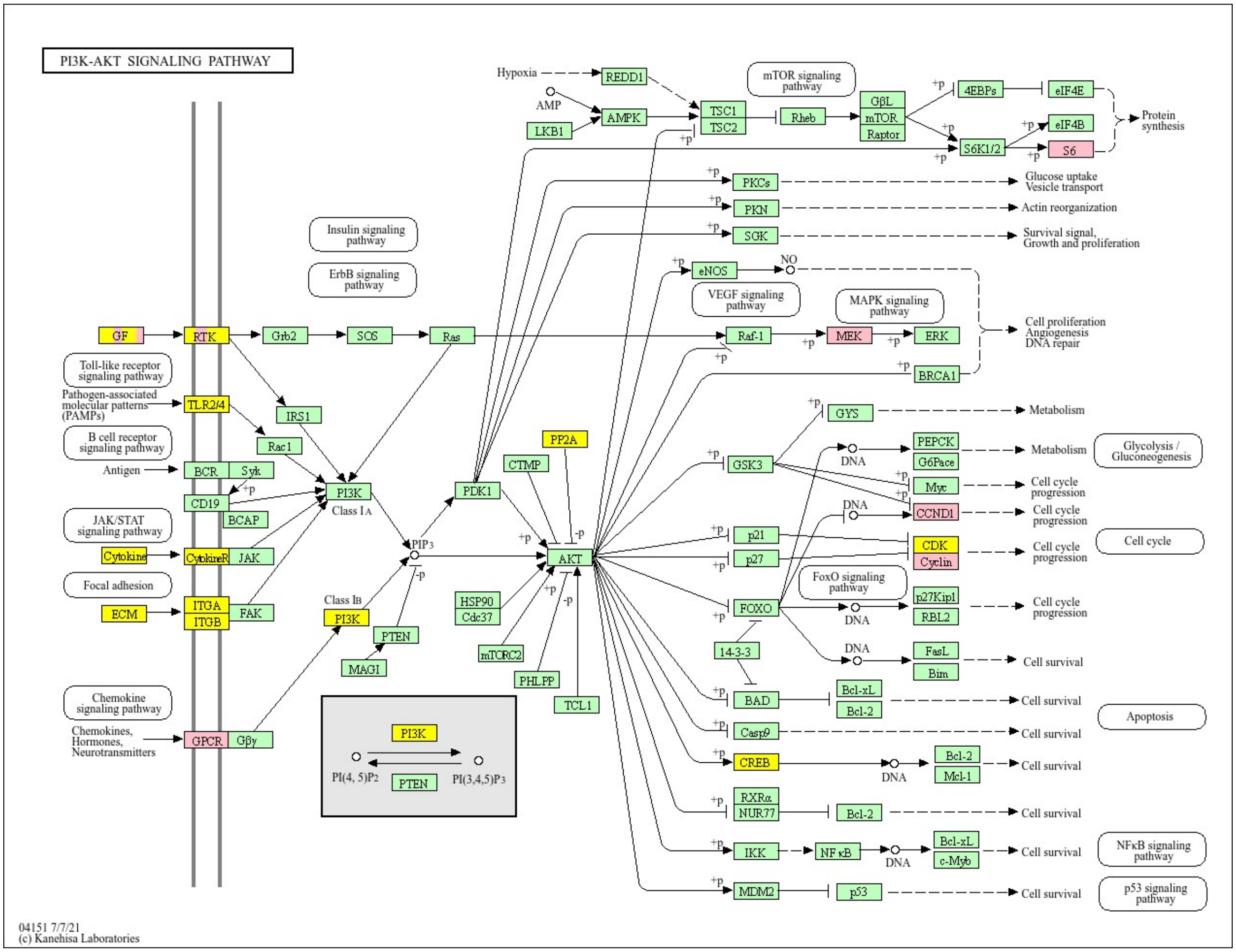

Supplementary Figure S4. Differentially expressed pathways in luminal A BRCA stratified by *WWOX*/*HIF1A* ratio. Key signalling pathways vary significantly with prognosis. Yellow indicates good prognosis, pink signifies poor prognosis, and green indicates genes with unchanged expression.

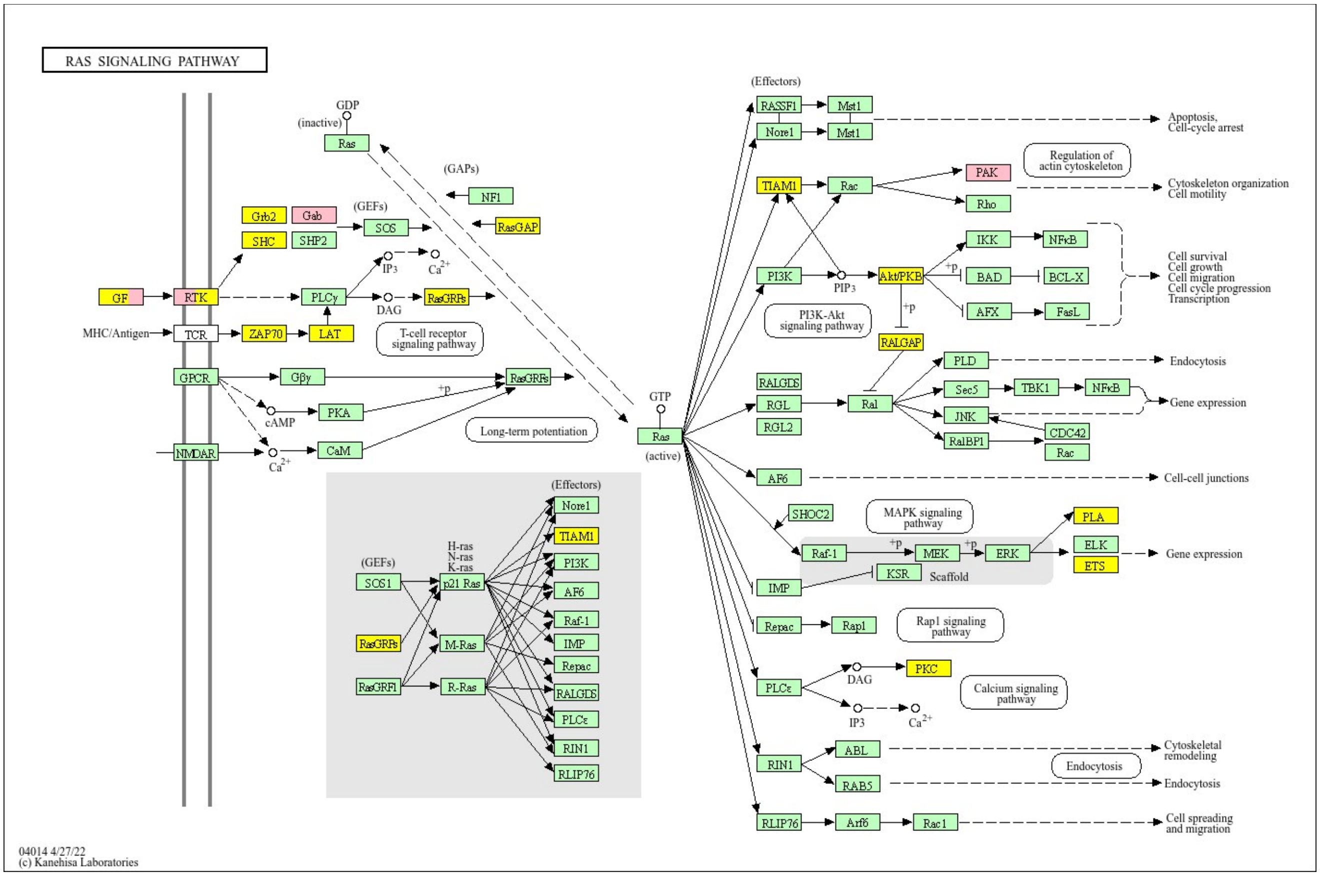

Supplementary Figure S5. Differentially expressed pathways in luminal B BRCA stratified by *WWOX*/*HIF1A* ratio. Key signalling pathways vary significantly with prognosis. Yellow indicates good prognosis, pink signifies poor prognosis, and green indicates genes with unchanged expression.

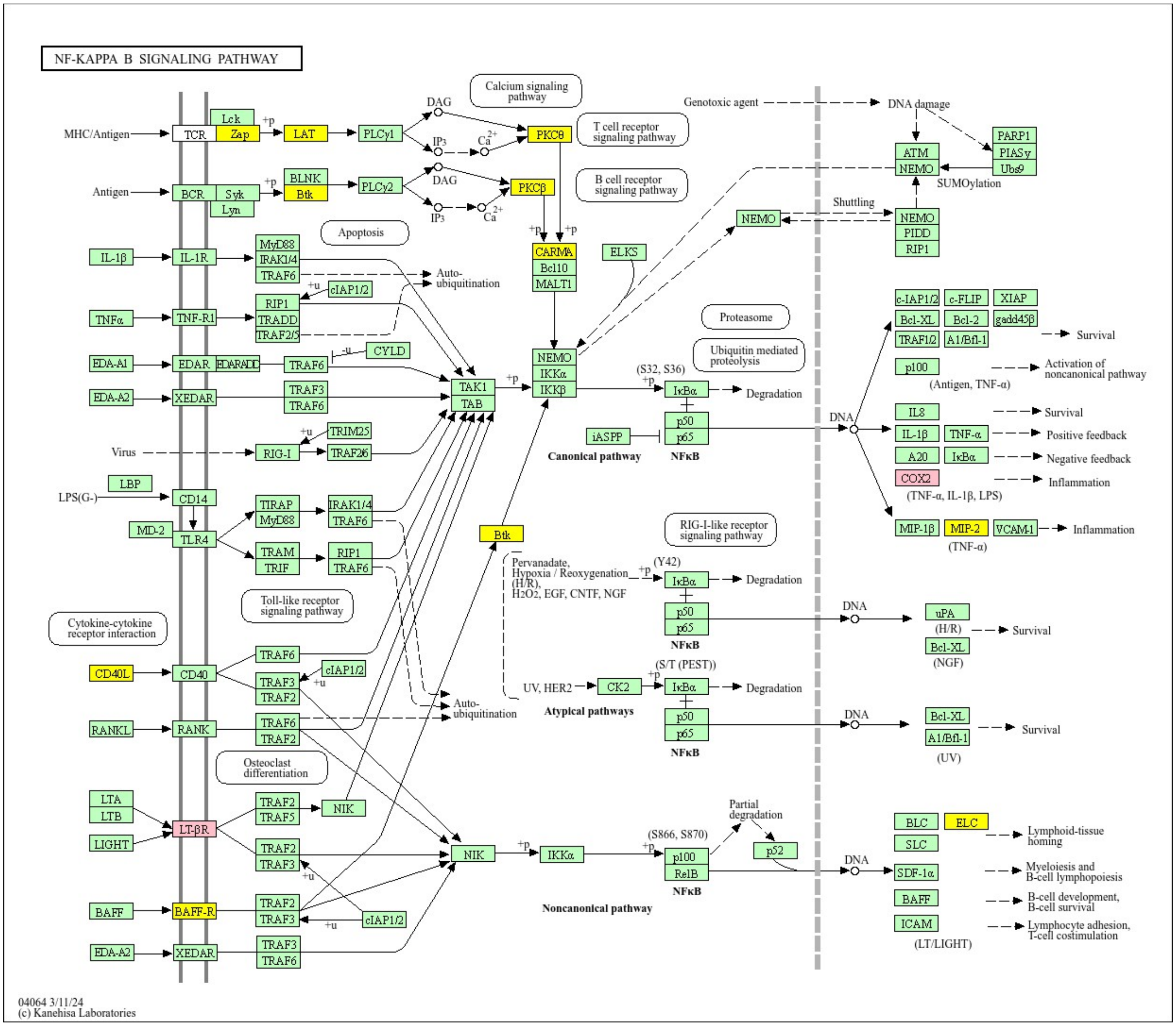

Supplementary Figure S6. Differentially expressed pathways in luminal B BRCA stratified by *WWOX*/*HIF1A* ratio. Key signalling pathways vary significantly with prognosis. Yellow indicates good prognosis, pink signifies poor prognosis, and green indicates genes with unchanged expression.

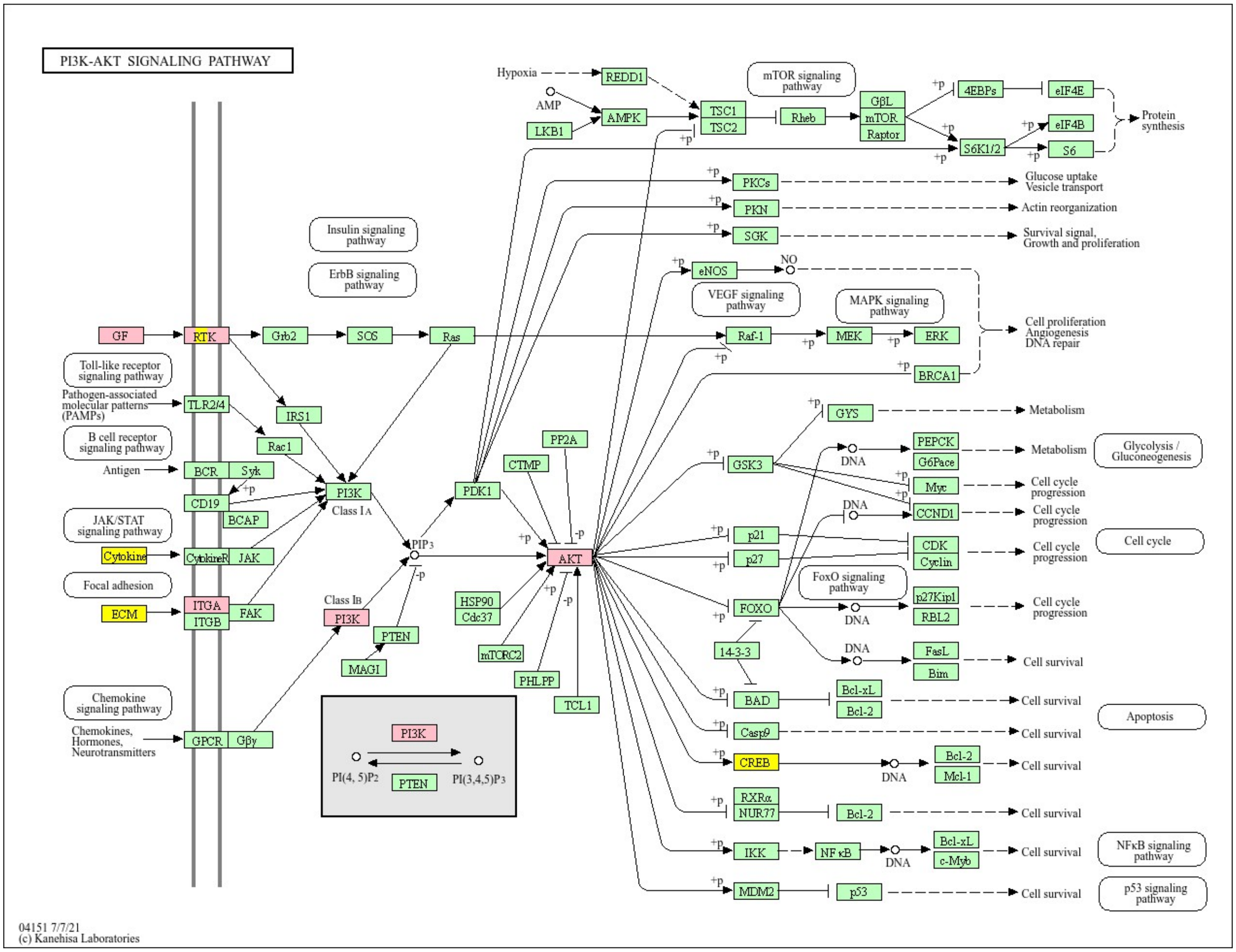

Supplementary Figure S7. Differentially expressed pathways in HER2-ENRICHED BRCA stratified by *WWOX/HIF1A* ratio. Key signalling pathways vary significantly with prognosis. Yellow indicates good prognosis, pink signifies poor prognosis, and green indicates genes with unchanged expression.

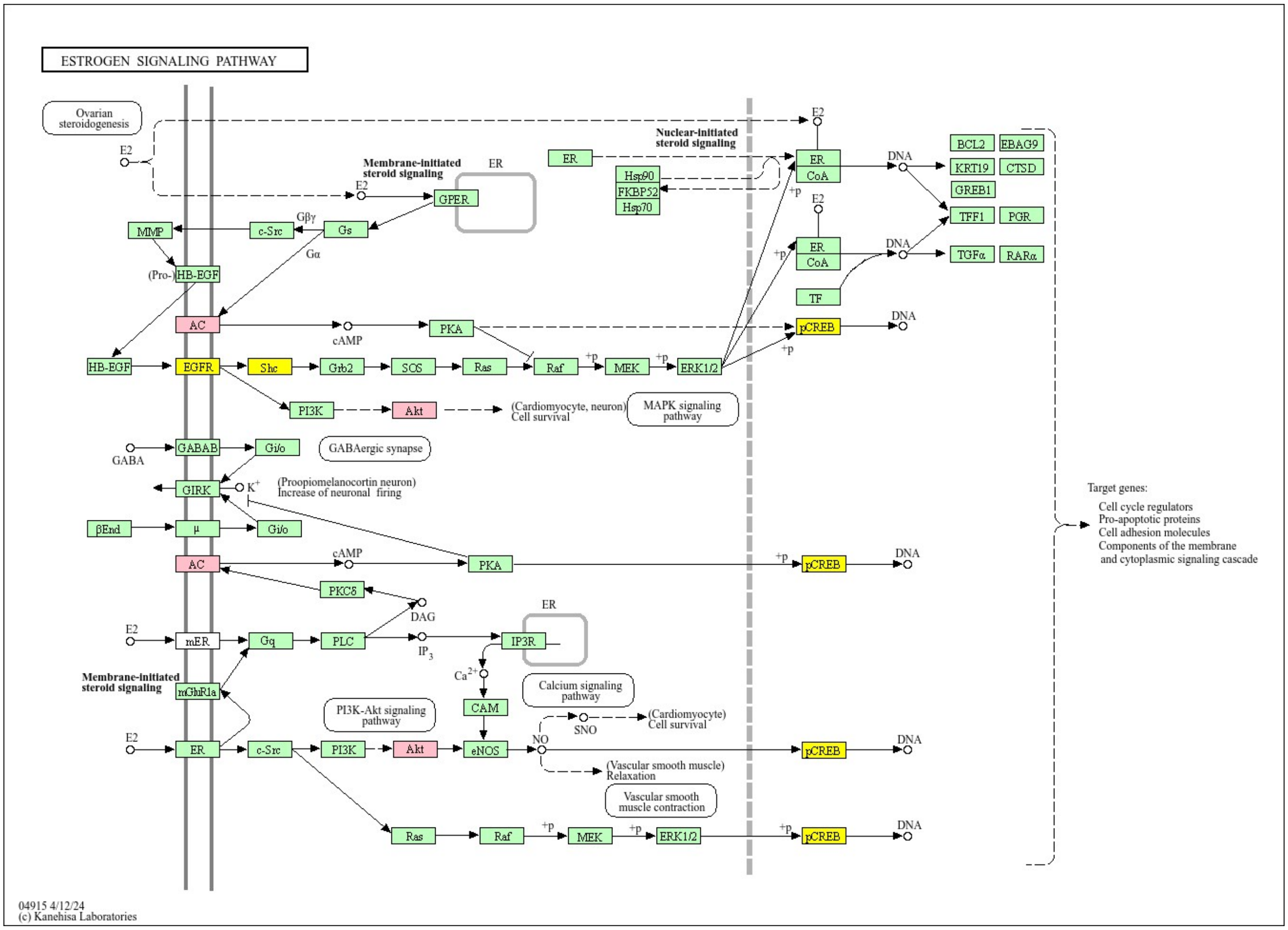

Supplementary Figure S8. Differentially expressed pathways in HER2-ENRICHED BRCA stratified by *WWOX/HIF1A* ratio. Key signalling pathways vary significantly with prognosis. Yellow indicates good prognosis, pink signifies poor prognosis, and green indicates genes with unchanged expression.

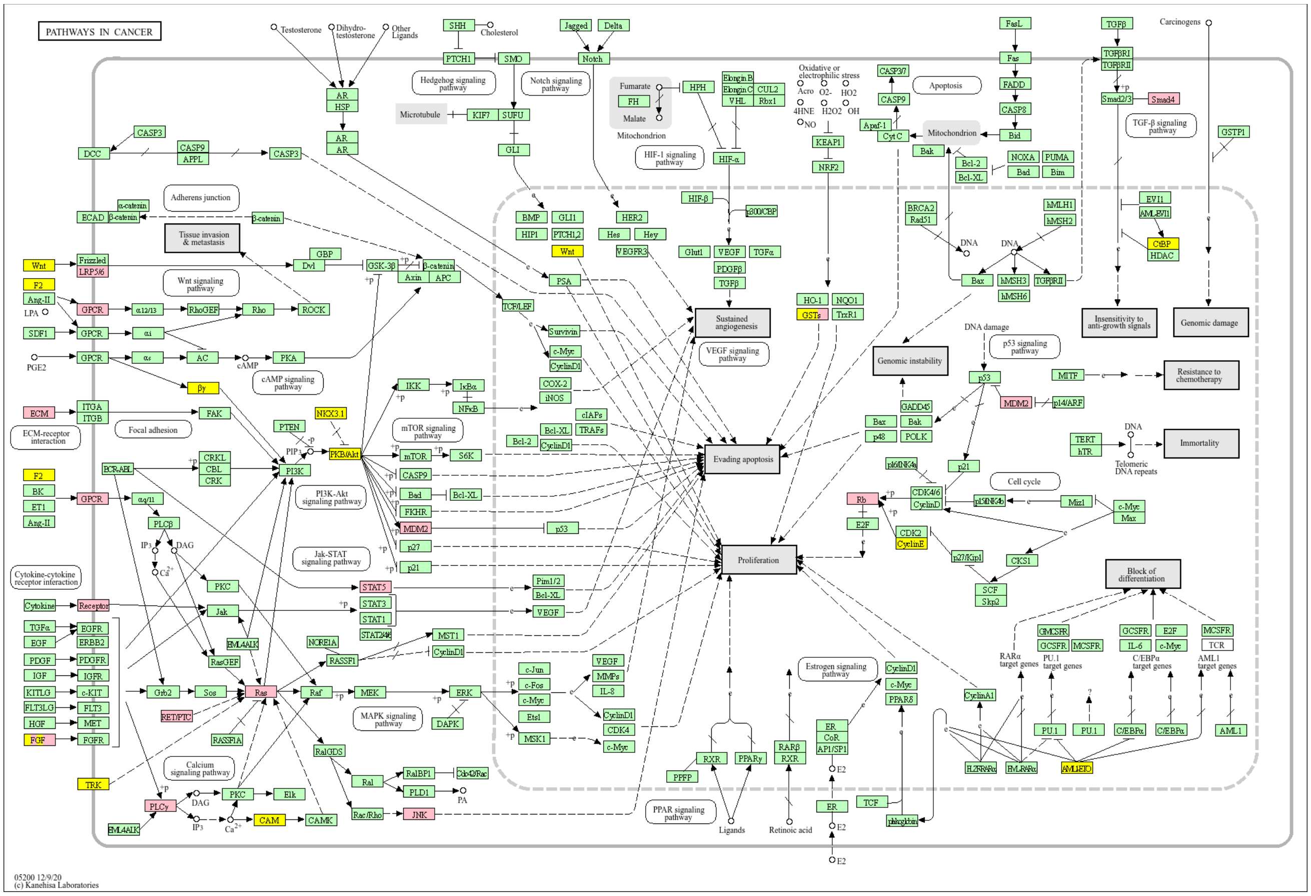

Supplementary Figure S9. Differentially expressed pathways in OV based on *WWOX/HIF1A* ratio. Yellow indicates pathways enriched in good-prognosis tumours, pink denotes pathways upregulated in poor-prognosis tumours, emphasizing the balance between metabolic regulation, immune response, and tumour aggressiveness, and green indicates genes with unchanged expression.

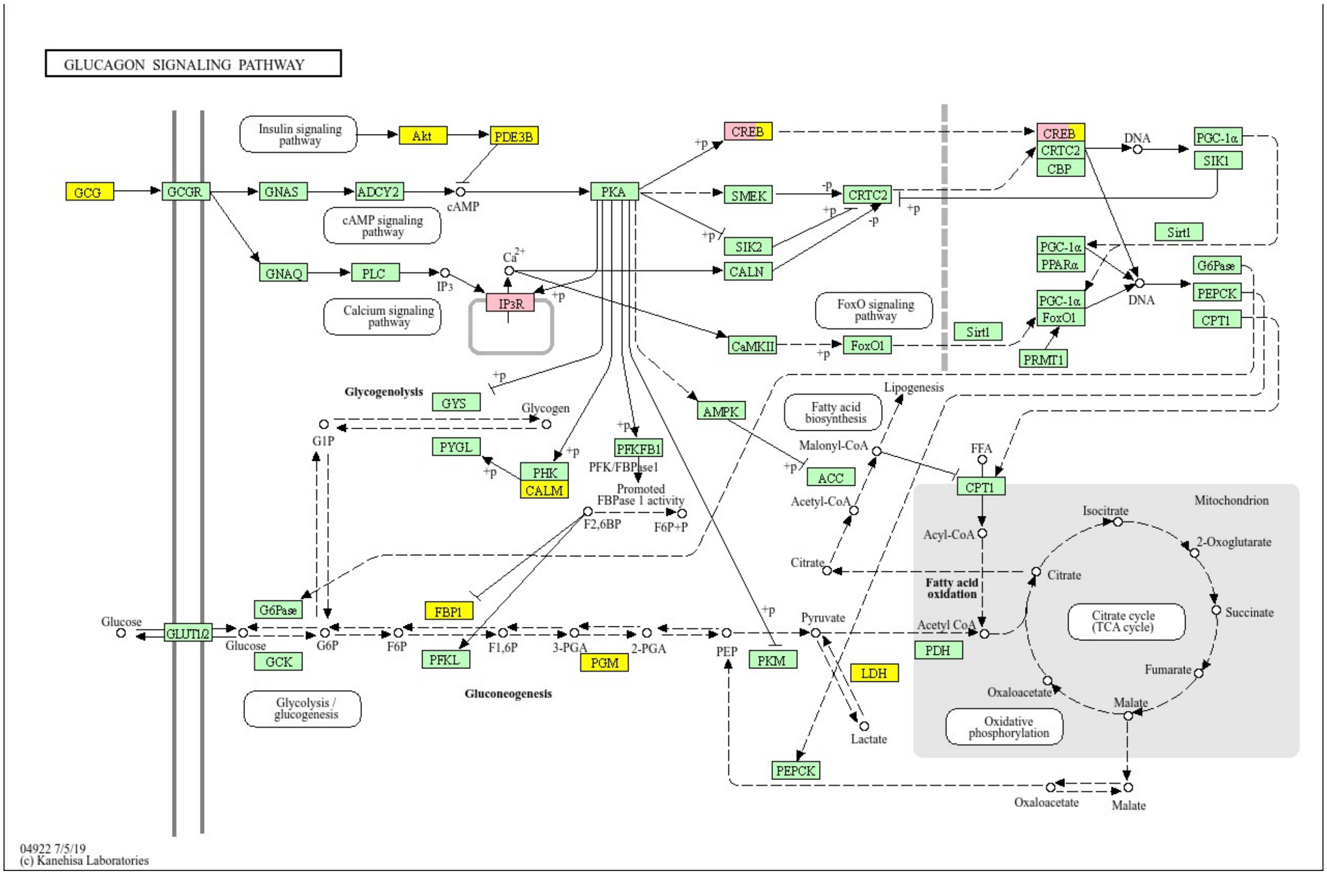

Supplementary Figure S10. The glucagon signalling pathway is differentially expressed in the OV and stratifies prognosis according to the *WWOX/HIF1A* ratio. Good prognosis tumours show higher expression of genes involved in oxidative metabolism and tumour suppression. In the figure, yellow indicates good prognosis, pink indicates poor prognosis, and green indicates genes with unchanged expression.

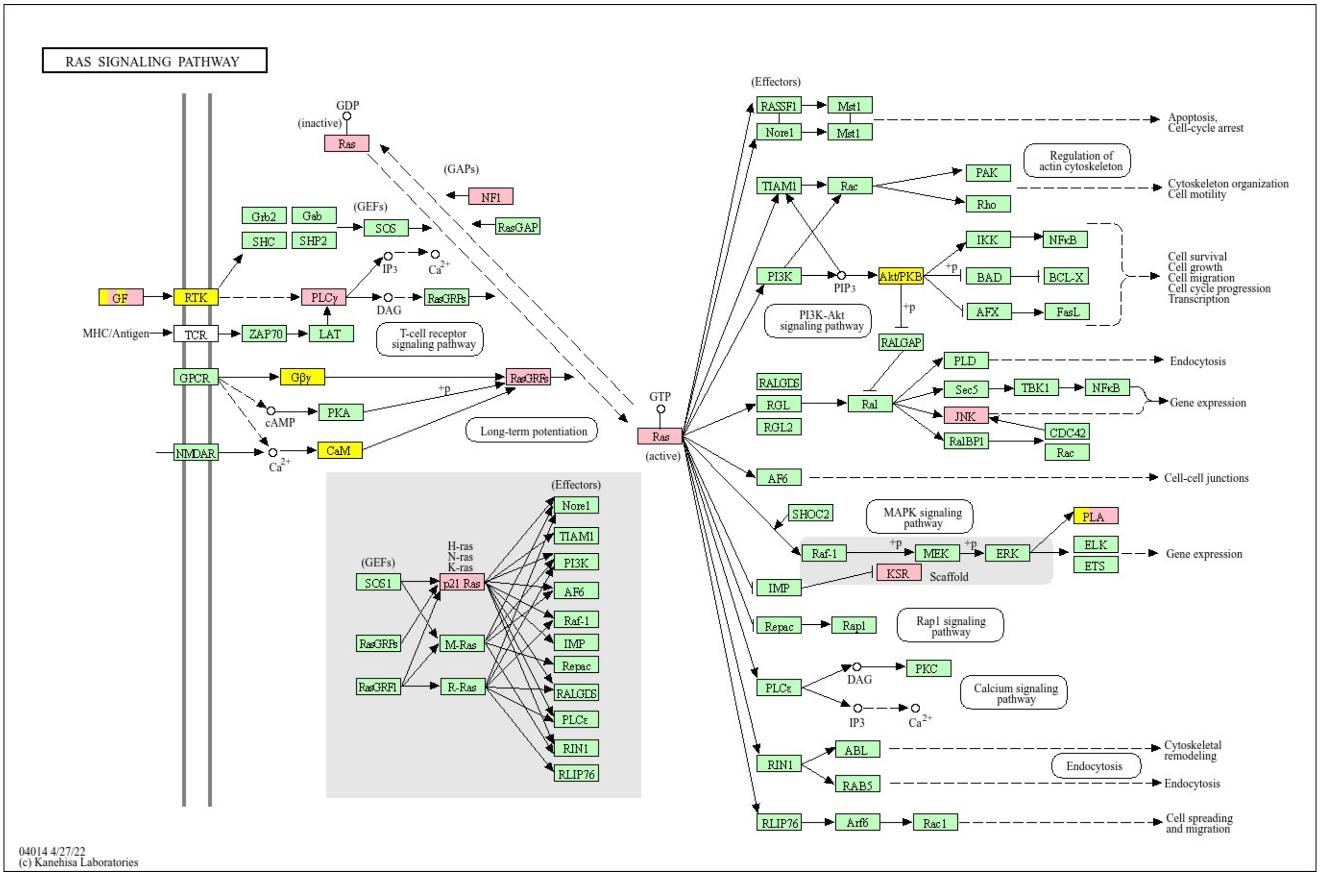

Supplementary Figure S11. The RAS signalling pathway is differentially expressed in the OV and stratifies prognosis according to the *WWOX*/*HIF1A* ratio. Good prognosis tumours show higher expression of genes involved in oxidative metabolism and tumour suppression. In the figure, yellow indicates good prognosis, pink indicates poor prognosis, and green indicates genes with unchanged expression.

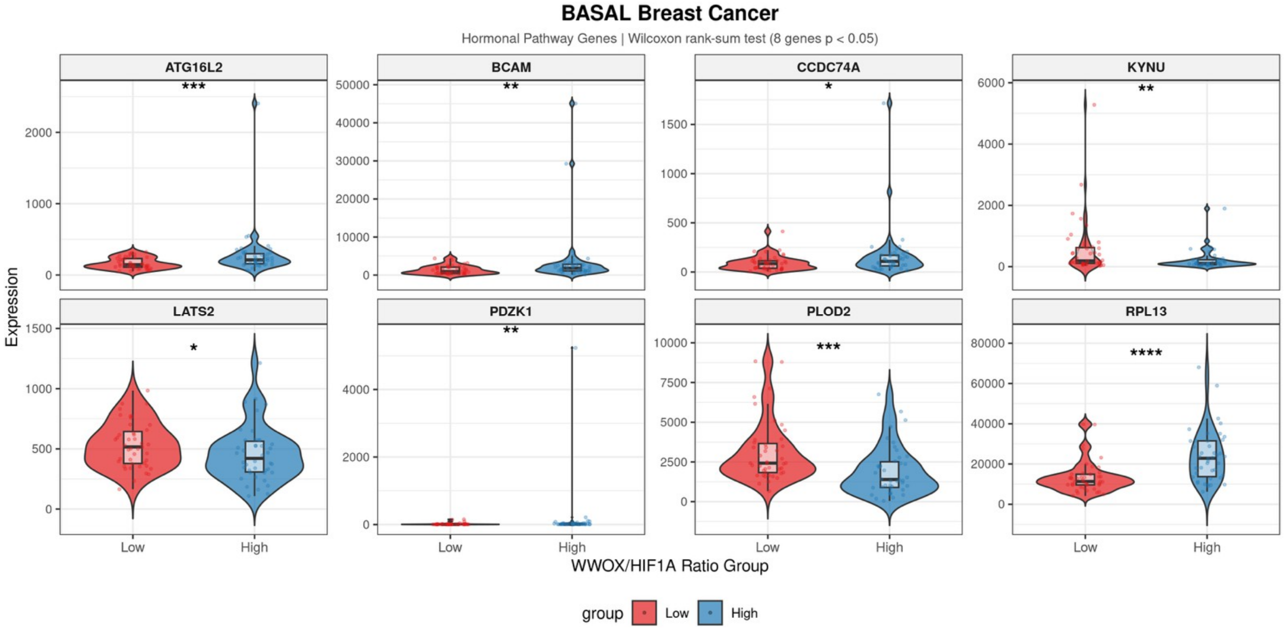

Supplementary Figure S12. Boxplot of significant genes in Luminal B BRCA, revealing marked expression differences.

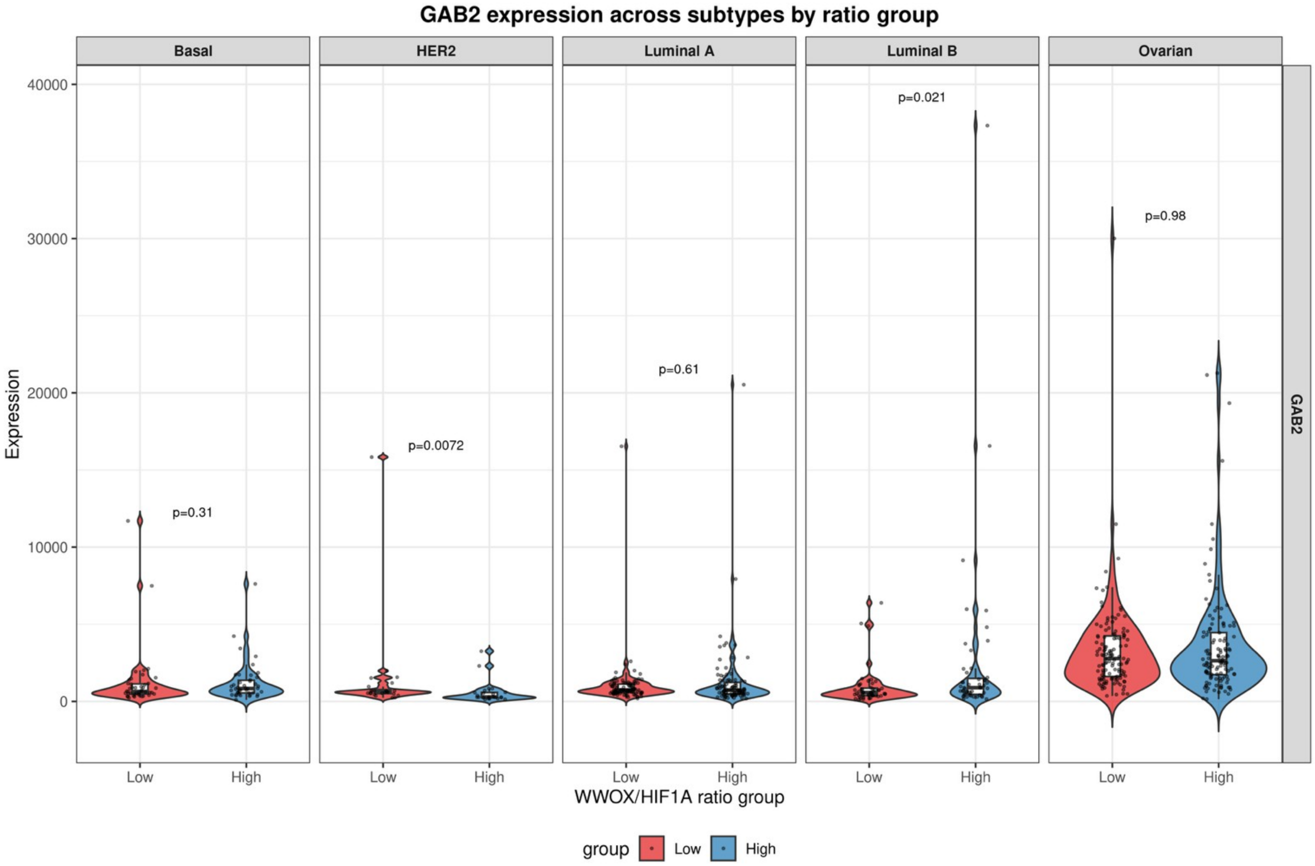

Supplementary Figure S13. Boxplot of *GAB2* (hormone pathway-related) in Luminal B BRCA, revealing marked expression differences.

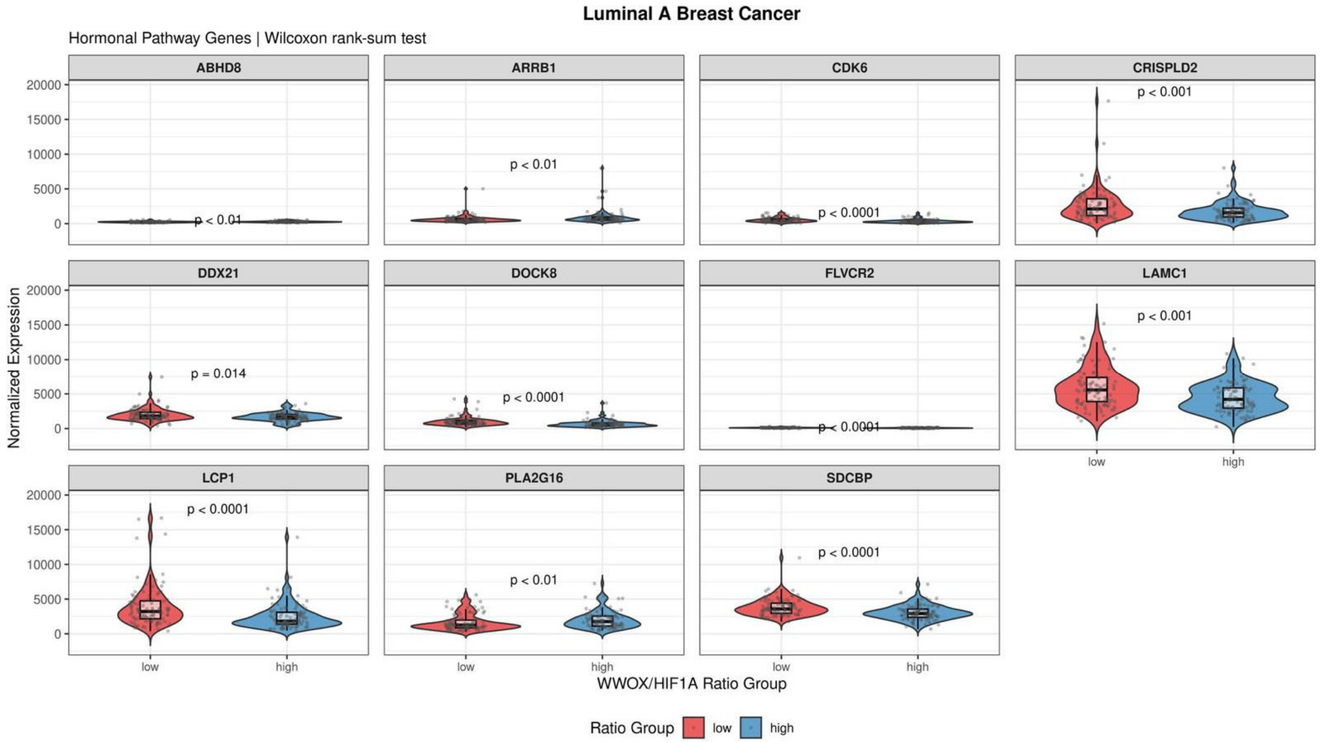

Supplementary Figure S14. Boxplots of significant genes in Luminal A BRCA (Wilcoxon $p \leq 0.05$), separating high and low expression groups.

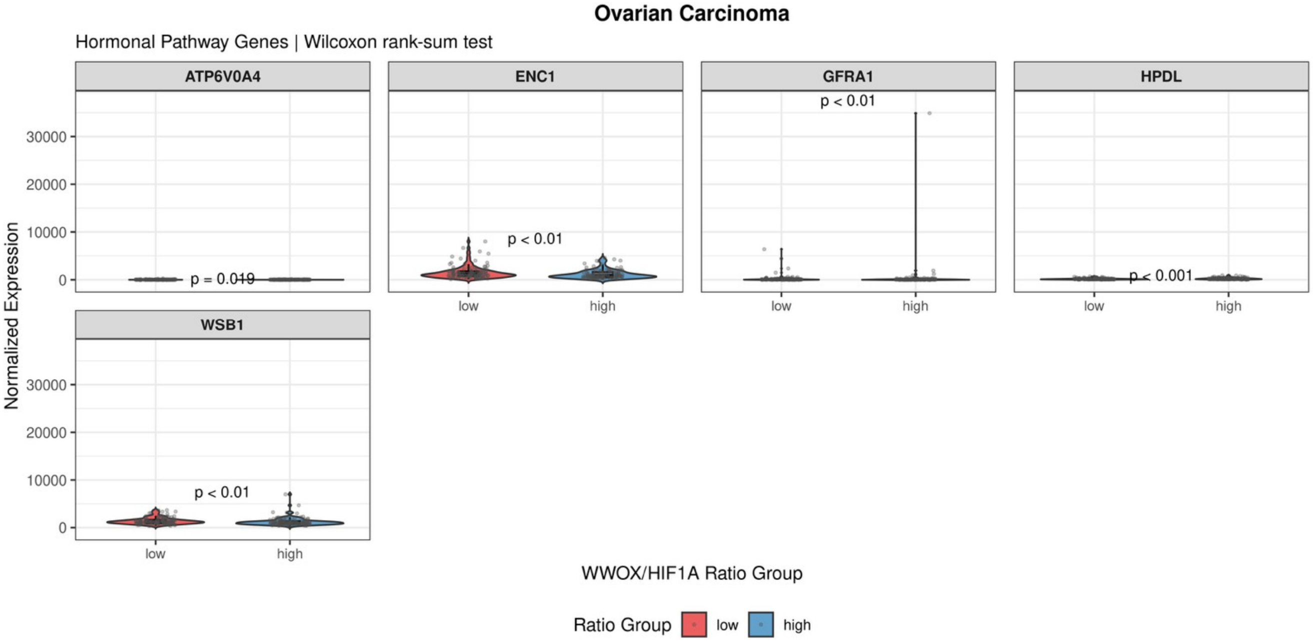

Supplementary Figure S15. Boxplots of significant hormone pathway genes in OV (Wilcoxon p ≤ 0.05), grouped by high/low expression cohorts.

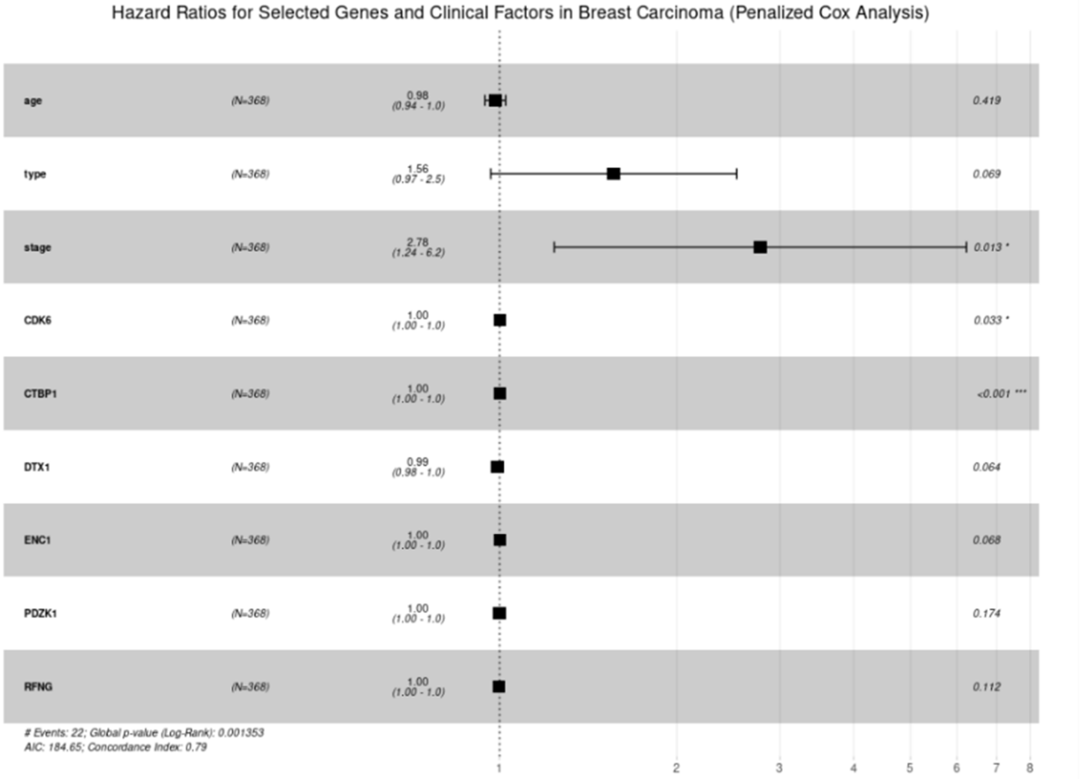

Supplementary Figure S16. Penalized Cox Regression in BRCA. Penalized Cox (LASSO) survival analysis in the BRCA cohort (N = 390; 22 events, 368 censored). A broader set of clinical and gene expression variables was entered into a LASSO-penalized Cox model with cross-validated λ; only predictors retaining non-zero coefficients after penalization are displayed. Hazard ratios and post-selection p-values are shown for descriptive characterization of the LASSO-selected features and should be interpreted as exploratory.

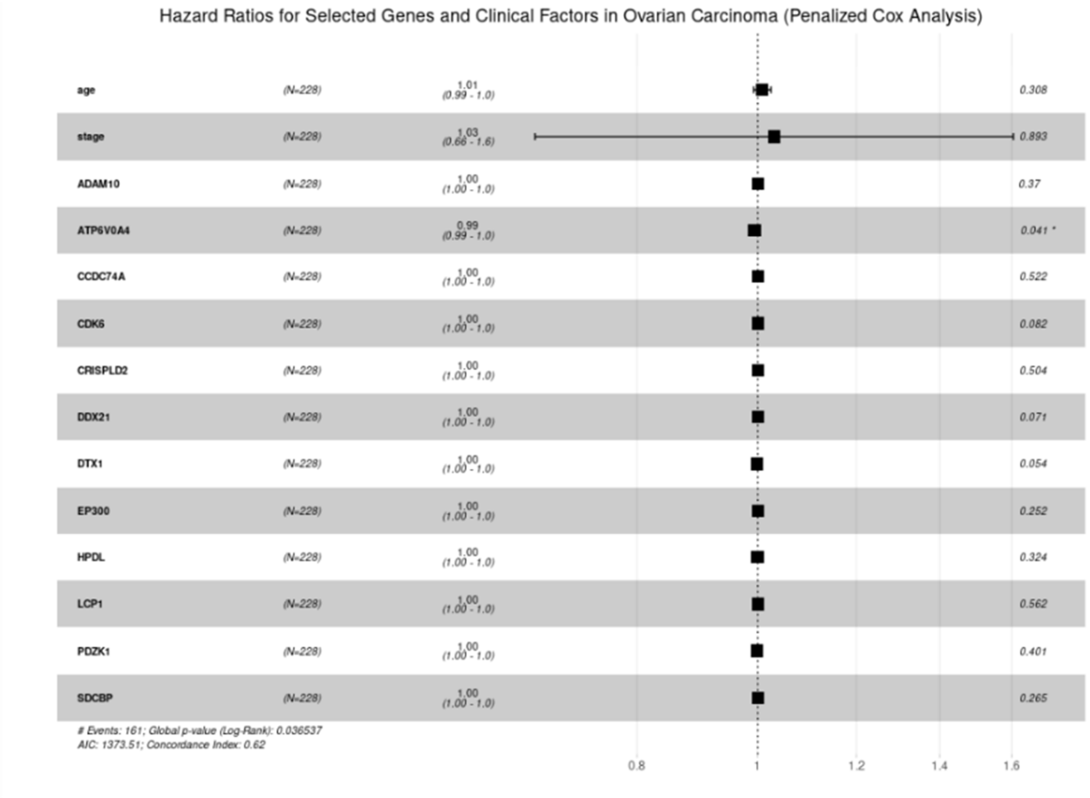

Supplementary Figure S17. Penalized Cox Regression in OV. Penalized Cox (LASSO) survival analysis in the OV cohort (N = 228; 161 events, 67 censored). Clinical and gene expression variables were subjected to LASSO-penalized Cox regression with cross-validated λ, and predictors with non-zero coefficients after penalization are displayed. Hazard ratios and post-selection p-values are presented for descriptive tracking of the selected features; given the higher number of events in this cohort, the model provides more stable prognostic information but still requires external validation.
