# Supplementary material for: WWOX/HIF1A Balance Delineates Context-Dependent Molecular States in Breast Cancer Subtypes and Ovarian Carcinoma
Source: Int J Mol Sci. 2026 Jul 28;27(15):6740. doi: 10.3390/ijms27156740 (PMC13467099; doi:10.3390/ijms27156740)
Supplement: Supplementary file 1 [file ijms-27-06740-s001.zip › ijms-4365276-Supplementary File S2.pdf]

ESR2 CHEA Transcription Factor Targets	ESR1 ENCODE	ESR1 CHEA Transcription Factor Targets	AR CHEA Transcription Factor Targets
ABAT	AAK1	A1BG	AAA1
ABCC5	AAMP	A4GALT	AADACL2
ABHD2	AANAT	AAK1	AADAT
ABHD8	AARS2	ABAT	AAGAB
ACAD10	ABAT	ABCA1	AARD
ACER3	ABCA12	ABCA4	AARS
ACOT4	ABCA17P	ABCA7	AARS2
ACOX3	ABCA3	ABCA8	AASDH
ADGRE5	ABCB8	ABCB1	AASDHPPT
ADORA1	ABCC11	ABCB11	ABAT
AFAP1L2	ABCC5	ABCB6	ABCA1
AGAP11	ABCD1	ABCC11	ABCA10
AGO2	ABCG1	ABCC3	ABCA13
AKAP1	ABHD11	ABCC5	ABCA4
AKR1C2	ABHD16B	ABCD2	ABCA9
ANGPTL4	ACAD8	ABCD4	ABCB1
ANK3	ACBD5	ABCG2	ABCB10
ANKRD1	ACER2	ABCG5	ABCB5
ANKRD13D	ACKR2	ABCG8	ABCC1
ANKRD23	ACOT9	ABHD11	ABCC2
ANKRD33	ACP6	ABHD12	ABCC3
APMAP	ACSF3	ABHD14A	ABCC4
ARHGEF16	ACTN1	ABHD2	ABCD3
ARID4B	ACVR1C	ABL1	ABHD11
ARRB1	ADAMTS8	ABLIM1	ABHD12
ARRDC2	ADAMTSL5	ABR	ABHD2
ASPSCR1	ADARB1	ACAA2	ABHD5
ATG16L2	ADAT3	ACACA	ABI1
ATP1B1	ADCY10	ACAD8	ABI3
ATP2C2	ADGRF1	ACADM	ABI3BP
ATP6V0A4	ADGRG1	ACAP2	ABL1
ATP6V0E1	ADHFE1	ACLY	ABL2
ATP6V1C2	ADK	ACO2	ABLIM2
ATXN1	AFAP1	ACOT12	ABLIM3
B3GALT4	AFF1	ACOT2	ABR
B4GALT1	AFG3L1P	ACOT4	ABTB2
BACE1	AGPAT2	ACOX1	ACAA1
BAG1	AGPS	ACOX2	ACACA
BANP	AGR2	ACOX3	ACACB
BARD1	AGR3	ACOXL	ACAD11
BATF	AHDC1	ACSL1	ACAD8
BCAM	AHNAK	ACSL5	ACADSB
BCL2	AHNAK2	ACTB	ACAN
BCL3	AIFM2	ACTBL2	ACAP2
BCL9L	AK3	ACTN1	ACAT2
BDKRB2	AKAP1	ACTN4	ACBD3
BMF	AKAP8	ACVR2A	ACBD6
BMP7	AKNA	ADAM12	ACER1
BTBD11	AKR1D1	ADAM19	ACER3
C14ORF132	ALDH18A1	ADAM24P	ACF
C16ORF74	ALDH3A1	ADAMDEC1	ACIN1
C17ORF58	ALDH3A2	ADAMTS18	ACKR2

C1ORF122	ALDH3B1	ADAMTS19	ACKR3
C1ORF21	ALDH3B2	ADAMTS2	ACO1
C1ORF216	ALKBH4	ADAMTS3	ACOT13
C1QTNF6	ALMS1P	ADAMTS5	ACOT7
C22ORF29	ALPI	ADAMTS6	ACOX3
C6ORF141	ALPP	ADAMTSL1	ACPP
C6ORF165	ALS2CL	ADAMTSL3	ACSL3
CA12	AMOTL2	ADCK3	ACSL4
CALCR	AMZ1	ADCY2	ACSL6
CALML5	ANAPC13	ADCY5	ACSM2B
CAMK2G	ANGPTL4	ADD3	ACSM3
CAP2	ANKRD1	ADGRA1	ACSS3
CAPN5	ANKRD12	ADGRA3	ACTA1
CAPN8	ANKRD2	ADGRD1	ACTBL2
CCDC103	ANKRD30BL	ADGRE5	ACTC1
CCDC64B	ANKRD40	ADGRF1	ACTL6A
CCDC74A	ANKRD62P1-PARP4P3	ADGRG1	ACTL7B
CCDC92	ANKS6	ADGRG2	ACTN2
CCPG1	ANXA1	ADGRG6	ACTN3
CCRN4L	ANXA11	ADH1A	ACTR2
CD55	ANXA2	ADH4	ACTR3
CD82	ANXA3	ADH7	ACTRT2
CD9	ANXA6	ADIPOR2	ACVR2A
CDC34	ANXA9	ADORA1	ACY1
CDH1	AOC3	ADRA1B	ACYP1
CDH10	AP2M1	ADRBK2	ACYP2
CDH3	AP3M1	AEBP1	ADAD1
CDK6	APBA2	AFAP1	ADAM10
CEBPA	APLF	AFAP1L2	ADAM15
CELSR2	APOA1	AFF1	ADAM19
CEMIP	APPL1	AFF3	ADAM20
CGN	APRT	AFP	ADAM22
CHD9	AQP5	AGO2	ADAM28
CHPT1	ARFGEF1	AGPAT1	ADAM29
CHSY1	ARFGEF2	AGR2	ADAM32
CLCF1	ARFGEF3	AGR3	ADAM7
CLDN23	ARFRP1	AGT	ADAMTS12
CLDN9	ARHGAP23	AGTR2	ADAMTS14
CMSS1	ARHGAP32	AHCY	ADAMTS15
CNST	ARHGAP35	AHCYL1	ADAMTS16
CNTD2	ARHGAP40	AHCYL2	ADAMTS17
CREBRF	ARHGEF19	AHNAK	ADAMTS18
CRISPLD2	ARHGEF37	AK2	ADAMTS2
CROT	ARHGEF38	AK3	ADAMTS20
CSRNP1	ARID3A	AK5	ADAMTS3
CSRP1	ARID5A	AKAP1	ADAMTS7
CTNND2	ARL14	AKAP13	ADAMTS8
CTSD	ARL3	AKAP7	ADAMTSL1
CYB5A	ARPC1B	AKNAD1	ADAMTSL3
CYP1A1	ARRB1	AKR1A1	ADARB1
CYP1B1	ARRDC1	AKT2	ADARB2
CYSRT1	ART4	ALCAM	ADCK2
DCAF4	ASB1	ALDH2	ADCK5
DCDC2	ASB13	ALDH3B2	ADCY1

DDX21	ASS1	ALG14	ADCY10
DDX47	ATF1	AMBRA1	ADCY2
DENND2C	ATG16L1	AMOTL2	ADCY5
DENND2D	ATG4D	AMPD3	ADCY7
DEPTOR	ATP10A	ANGEL1	ADCY8
DHRS2	ATP13A2	ANGPT4	ADD1
DHRS3	ATP1A1	ANGPTL4	ADD2
DIO1	ATP8B2	ANK3	ADD3
DIP2C	AZI2	ANKFN1	ADGRA1
DLL1	B3GALNT2	ANKRD13D	ADGRA3
DNAAF1	B3GALT4	ANKRD28	ADGRB3
DNAJB6	B3GNT3	ANKRD30B	ADGRD1
DOCK1	B3GNT6	ANKRD44	ADGRE1
DOCK8	B4GALT5	ANKRD50	ADGRF1
DOK7	BAD	ANKRD55	ADGRF3
DOPEY2	BAIAP2L1	ANO1	ADGRG6
DSCAM	BANP	ANO2	ADGRG7
EDC3	BATF	ANO6	ADGRL2
EDN1	BAZ2B	ANPEP	ADGRL3
EFHD1	BCAM	ANTXR1	ADGRL4
EFNA1	BCAP29	ANTXR2	ADGRV1
EFR3B	BCAP31	ANXA11	ADH4
ELF3	BCAS1	ANXA2	ADH6
ELMSAN1	BCL3	ANXA3	ADH7
ENC1	BCL6	ANXA5	ADI1
ENTPD3	BCR	ANXA6	ADIG
ENTPD8	BFSP1	AP3B1	ADK
EPHA2	BHLHE40	AP3S2	ADO
EPS15L1	BICC1	AP5S1	ADORA1
ERC2	BICD2	APBB2	ADORA2B
ESR2	BIRC5	APCDD1	ADRA1B
FAM174B	BLNK	APLF	ADRA1D
FAM213A	BLVRB	APOA2	ADRA2A
FAXDC2	BMF	APOF	ADRA2C
FCAR	BMS1	APPBP2	ADRB1
FKBP4	BNC1	APTX	ADRB2
FLNB	BNC2	AR	ADRB3
FLVCR2	BOC	ARAP3	ADRBK1
FREM2	BOLA1	AREL1	ADRBK2
FUT4	BOP1	ARFGAP3	ADSS
FXR1	BPESC1	ARFGEF1	ADTRP
FXYD3	BRD3	ARG1	AFAP1
GAB2	BRI3BP	ARGLU1	AFAP1L1
GALNT16	BRWD1-IT2	ARHGAP12	AFAP1L2
GDF15	BSG	ARHGAP18	AFF1
GDPD3	BTBD17	ARHGAP20	AFF2
GFRA1	BTBD18	ARHGAP28	AFF3
GLA	BTBD19	ARHGAP31	AFG3L2
GNA14	BTG4	ARHGEF10L	AFTPH
GPR37L1	C10ORF128	ARHGEF12	AGA
GPRC5A	C10ORF25	ARHGEF17	AGAP1
GPRC5C	C10ORF91	ARHGEF19	AGAP3
GPSM1	C11ORF52	ARHGEF3	AGBL1
GRAMD1A	C14ORF132	ARID1B	AGBL3

GRB10	C14ORF180	ARID2	AGBL4
GREB1	C15ORF39	ARL3	AGGF1
GREB1L	C15ORF52	ARMC2	AGK
GSTM3	C15ORF62	ARPP19	AGO2
GTF2IRD1	C16ORF13	ARRDC2	AGO3
H19	C17ORF99	ARRDC4	AGPAT3
HCAR1	C18ORF63	ARSG	AGPS
HEG1	C19ORF60	ART3	AGR2
HES1	C19ORF70	AS3MT	AGR3
HEY1	C1ORF100	ASAP1	AGT
HIPK2	C1ORF122	ASB16	AGTPBP1
HK1	C1ORF159	ASGR1	AGTR1
HOMER2	C1ORF168	ASGR2	AGTRAP
HOXC13	C1ORF186	ASIC2	AHDC1
HPDL	C1ORF216	ASPA	AHNAK
HS6ST2	C1ORF229	ASPG	AHRR
HSPA4	C1QL1	ASS1	AIDA
HSPB8	C1QTNF3	ASUN	AIFM1
IER2	C1QTNF6	ATF3	AIG1
IER3	C20ORF195	ATF6	AIM1
IFT122	C22ORF29	ATG3	AJAP1
IGDCC3	C22ORF39	ATG4C	AK5
IGFBP4	C2ORF54	ATG7	AKAP1
IGFL1	C4ORF19	ATHL1	AKAP10
IGSF9	C4ORF46	ATP10A	AKAP12
IL17RB	C5AR2	ATP12A	AKAP13
INSIG2	C5ORF30	ATP1B1	AKAP14
IRF2BPL	C5ORF49	ATP2B1	AKAP17A
IRS1	C5ORF56	ATP2B4	AKAP2
IRS2	C5ORF64	ATP2C2	AKAP5
ISG20	C6ORF132	ATP5L	AKAP6
ITGA5	C6ORF165	ATP6V0A4	AKAP7
JAK2	C6ORF223	ATP6V0C	AKAP8
JPH3	C7ORF57	ATP8A1	AKAP8L
JUNB	C8ORF86	ATP8B1	AKNA
KANK1	C9ORF131	AUTS2	AKR1B1
KCNK1	C9ORF142	AVP	AKR1C2
KCNMA1	C9ORF152	B2M	AKR1D1
KEAP1	CA12	B3GALT1	AKR7A2
KIAA0907	CABIN1	B3GALTL	AKT3
KIAA1467	CABLES2	B3GAT1	ALAS2
KLF9	CACHD1	B3GNTL1	ALDH1A1
KLHDC9	CACTIN-AS1	B4GALT1	ALDH1A2
KRT13	CADPS2	B4GALT5	ALDH1A3
KRT15	CALCOCO1	BACE2	ALDH1B1
KYNU	CALM2	BAHCC1	ALDH1L1
LAMC1	CAND1	BAIAP2L1	ALDH1L2
LATS2	CANT1	BANF2	ALDH2
LCP1	CAP2	BANP	ALDH3A2
LDHD	CAPN10	BARX2	ALDH4A1
LMCD1	CAPN15	BBC3	ALDH5A1
LOC145837	CAPN2	BBOX1	ALDH7A1
LOC284379	CAPN8	BCAM	ALDH8A1
LOC285696	CAPN9	BCAN	ALDOB

LRIG1	CAPS	BCAR3	ALG2
LRRC8D	CARD10	BCAS1	ALG6
MACF1	CASP4	BCAS3	ALG8
MAOA	CASP7	BCL2	ALK
MARCKS	CATIP	BCL3	ALKBH3
MB	CATSPER3	BCL6	ALLC
MB21D2	CBLC	BCL7B	ALMS1
MBNL2	CBWD1	BCL9	ALOX5
MBOAT1	CBWD2	BFSP1	ALOXE3
MCCC2	CBWD3	BICC1	ALPI
METTL2A	CBWD5	BIK	ALPK3
MICB	CBWD6	BIRC5	ALPL
MID1IP1	CCDC146	BLNK	ALX3
MIDN	CCDC159	BMF	AMACR
MITF	CCDC176	BMP2K	AMD1
MREG	CCDC33	BMP4	AMELX
MTCP1	CCDC34	BMP7	AMER2
MTRR	CCDC57	BMPER	AMFR
MX1	CCDC74B	BNC2	AMIGO1
MYB	CCDC88C	BNIP1	AMN1
MYC	CCL15	BPHL	AMOTL1
MYEOV	CCL28	BPIFC	AMPH
MYH9	CCND1	BRCA1	AMTN
MYPN	CCNO	BRE	ANAPC1
N4BP3	CD3EAP	BRF1	ANAPC13
NANS	CD46	BRIP1	ANAPC15
NAV2	CD55	BSG	ANAPC16
NBEA	CD82	BST2	ANGPT1
NBPF1	CD9	BTBD10	ANGPT4
NDRG1	CDC14A	BTBD11	ANGPTL1
NFIC	CDCA3	BTBD3	ANGPTL6
NHS	CDCA7	BTBD9	ANK1
NKD2	CDCA7L	BTG1	ANK2
NOP16	CDH1	C10ORF2	ANK3
NOTCH3	CDH18	C11ORF49	ANKEF1
NPAS3	CDH2	C12ORF49	ANKFN1
NPNT	CDK12	C12ORF71	ANKFY1
NRIP1	CDK17	C14ORF159	ANKK1
NT5C2	CDK18	C15ORF26	ANKMY2
NUAK1	CDK5	C15ORF27	ANKRA2
OGFR	CDS1	C16ORF58	ANKRD1
OPA3	CEBPE	C16ORF74	ANKRD10
OPRL1	CECR6	C16ORF87	ANKRD11
OSBPL7	CELF1	C17ORF82	ANKRD12
P2RY6	CELSR1	C1ORF189	ANKRD13A
P3H4	CENPBD1	C1ORF21	ANKRD13C
PACSIN1	CENPT	C1ORF210	ANKRD13D
PALM	CENPV	C1ORF233	ANKRD18B
PARM1	CEP135	C1QTNF3	ANKRD19P
PCSK6	CEP63	C1QTNF6	ANKRD20A11P
PCTP	CERS1	C1QTNF7	ANKRD27
PDLIM1	CES2	C20ORF196	ANKRD28
PDZK1	CETN3	C20ORF85	ANKRD29
PFKFB3	CFAP45	C2CD2L	ANKRD30A

PHLDB1	CFD	C3	ANKRD31
PIAS3	CGN	C4ORF19	ANKRD33
PIAS4	CHD9	C6ORF141	ANKRD33B
PID1	CHDH	C7ORF55	ANKRD34C
PIGB	CHMP4B	C8ORF34	ANKRD35
PIP4K2A	CHPT1	C9	ANKRD42
PITPNC1	CHRNE	CA12	ANKRD44
PKIB	CHST15	CA13	ANKRD45
PKNOX1	CHURC1	CA3	ANKRD50
PLA2G16	CHURC1-FNTB	CA8	ANKRD52
PLAC1	CIB4	CABIN1	ANKRD53
PLCG2	CIC	CACNA1D	ANKRD55
PLEKHF1	CILP	CACNA2D1	ANKRD9
PLEKHF2	CIPC	CACNA2D2	ANKS3
PLEKHN1	CISD3	CACNA2D3	ANLN
PLOD2	CITED2	CACNG6	ANO1
PNPLA7	CKB	CALCR	ANO10
PODXL	CKS1B	CALD1	ANO3
PPAP2B	CKS2	CALM1	ANO4
PPFIBP2	CLASP2	CALML4	ANO6
PPL	CLCF1	CALML5	ANP32A
PPP2R2C	CLDN16	CAMK1G	ANTXR2
PRKCD	CLDN25	CAMK2D	ANXA2
PRKRIP1	CLDN4	CAP2	ANXA2P1
PRPS1	CLDND1	CAPN13	ANXA2P2
PRRT2	CLEC16A	CAPN5	ANXA3
PSTPIP2	CLN8	CAPN8	ANXA4
PTGER3	CLP1	CARD10	ANXA5
PTGES	CLPSL1	CARD11	AOAH
PTP4A2	CLPSL2	CASP7	AP1G1
PTRH1	CLSTN3	CASP8	AP1M1
PVT1	CLTB	CASP8AP2	AP2A1
QSOX1	CLUH	CASR	AP2A2
RAB15	CMPK1	CASZ1	AP2B1
RAB26	CMTM2	CAV2	AP3B1
RAB37	CNDP2	CBFA2T3	AP3D1
RAB4B	CNOT11	CBL	AP3M2
RAD54B	CNTD2	CBS	AP3S1
RALGDS	COL12A1	CBX2	AP3S2
RAPGEFL1	COL14A1	CCDC117	AP4B1
RASA3	COL9A2	CCDC125	AP5S1
RASEF	COLEC12	CCDC141	APBA1
RCAN1	COPS7B	CCDC171	APBA2
RET	COQ2	CCDC42	APBB1IP
RHOB	COX4I1	CCDC57	APBB2
RHOBTB3	COX7C	CCDC6	APC
RHPN2	CP	CCDC63	APCDD1
RIMS3	CPA4	CCDC65	APIP
RIPK4	CPEB3	CCDC68	APLNR
RNF170	CPM	CCDC69	APLP2
RPL13	CPT1B	CCDC71L	APMAP
RPL23AP42	CPXM1	CCDC80	APOA4
RUSC2	CRIP2	CCDC83	APOBEC3F
S100P	CRKL	CCL28	APOC2

S1PR3	CRTC1	CCNH	APOC4
SASH1	CRTC2	CCNI	APOD
SDCBP	CRYAA	CCR6	APOL3
SEC14L2	CRYGC	CD180	APOL4
SELT	CSDE1	CD1D	APOO
SEMA3B	CSRNP2	CD320	APP
SEMA3E	CSRP3	CD44	APPBP2
SEPT9	CST6	CD55	AQP12A
SERPINA3	CSTB	CD81	AQP2
SFXN2	CT62	CD82	AQP9
SGK1	CTBP2	CD83	AQR
SGK3	CTDP1	CD86	AR
SH3BP4	CTDSP1	CDC14A	ARAF
SHANK2	CTGF	CDC42EP1	ARAP1
SHROOM2	CTSD	CDC7	ARAP2
SIDT2	CUEDC1	CDCA7	ARAP3
SIGIRR	CXCL17	CDCP2	AREG
SIK1	CXORF38	CDH11	ARFGAP2
SLC16A6	CXXC5	CDH12	ARFGEF2
SLC19A1	CYB561	CDH13	ARFIP1
SLC22A23	CYB561D2	CDH5	ARG2
SLC29A1	CYB5D1	CDK14	ARGFXP2
SLC2A1	CYFIP2	CDKAL1	ARGLU1
SLC2A10	CYP1A1	CDKN1A	ARHGAP11A
SLC37A3	CYP1B1	CDKN2AIP	ARHGAP21
SLC7A5	CYP2B6	CDKN2B	ARHGAP24
SLC9A2	CYP2R1	CDKN3	ARHGAP26
SLCO2A1	CYP51A1	CDON	ARHGAP27
SLITRK4	CYTH3	CDRT4	ARHGAP28
SMARCA2	D2HGDH	CEACAM3	ARHGAP29
SNX29	DACT2	CECR5	ARHGAP32
SOCS3	DAP3	CELF2	ARHGAP35
SOX13	DAPK2	CELSR2	ARHGAP5
SOX18	DBR1	CEMIP	ARHGAP6
SOX3	DCAF10	CENPA	ARHGAP8
SOX9	DCAF6	CEP112	ARHGDIB
SPATS2	DCAKD	CEP120	ARHGEF10
SPIRE2	DCBLD1	CEP128	ARHGEF11
SPTBN2	DCLK2	CEP162	ARHGEF12
SQSTM1	DCUN1D3	CERK	ARHGEF18
SRPK1	DCUN1D5	CERS1	ARHGEF26
ST3GAL1	DDAH1	CERS2	ARHGEF28
STC2	DDAH2	CES3	ARHGEF3
SULT2B1	DDIT4	CGN	ARHGEF37
SVIL	DDO	CGNL1	ARHGEF7
SYBU	DDX23	CHAT	ARID1A
TAX1BP3	DDX3X	CHD1L	ARID2
TCF12	DDX47	CHD3	ARID5B
TDRD1	DDX51	CHD9	ARIH1
TFAP2C	DDX58	CHMP1A	ARL13A
TFF1	DECR1	CHRM3	ARL13B
TGFB2	DERL1	CHST11	ARL14
TGFBRAP1	DGAT1	CHST9	ARL14EP
TGM2	DHCR24	CHUK	ARL16

THBS1	DHFRL1	CISH	ARL2BP
TIA1	DHRS3	CITED2	ARL4A
TIPARP	DHRSX	CKB	ARL4C
TLN2	DHX8	CLCA2	ARL4D
TMEM63A	DIAPH3	CLCF1	ARL5B
TMEM79	DIP2C	CLDN10	ARL8B
TNFRSF12A	DIRC1	CLDN14	ARMC1
TNIP1	DKFZP434A062	CLDN23	ARMC2
TNS3	DKKL1	CLDN9	ARMC3
TOM1L2	DMRT2	CLEC14A	ARMC5
TOP1	DNAJA2	CLEC16A	ARMC7
TPD52L1	DNAJC5B	CLIC4	ARMC9
TRAK1	DNAL4	CLIP1	ARNT2
TSC22D3	DNMBP	CLMN	ARNTL2
TUFT1	DOK2	CLPSL2	ARPC3
UBTD1	DOK7	CLPX	ARR3
UGDH	DOPEY1	CLSTN1	ARRDC1-AS1
UNKL	DSCAM-AS1	CMAS	ARRDC4
USP10	DSCR3	CMIP	ARSE
VAMP5	DSN1	CMKLR1	ARSG
VASN	DSTN	CMSS1	ARSK
VAV2	DTD2	CNGB3	ART4
VPS9D1	DTWD1	CNN3	ARV1
VTCN1	DUSP23	CNR1	ARX
VWF	DUSP6	CNST	ASAP1
WBP4	E2F2	CNTN2	ASAP2
WFS1	E2F8	CNTNAP1	ASB12
WIPI1	E4F1	COBL	ASB13
WISP2	EBAG9	COL15A1	ASB14
WNT7B	ECEL1P2	COL1A2	ASB2
WSB1	ECH1	COL23A1	ASB4
WWC3	ECI1	COL26A1	ASB6
WWP2	ECSIT	COL3A1	ASB7
XBP1	EDN1	COL4A2	ASCC1
ZBED4	EDN2	COL4A3BP	ASCC2
ZCCHC8	EEA1	COL4A4	ASCC3
ZCWPW1	EEF2K	COL4A6	ASCL1
ZNF217	EEF2KMT	COL6A1	ASF1A
ZNF365	EFCAB14	COL6A2	ASIC2
ZNF385A	EGOT	COL6A3	ASIC3
ZNF462	EHBP1L1	COL6A5	ASIC4
ZNF703	EHD1	COL8A1	ASL
ZNF823	EHD4	COL8A2	ASNSD1
	EIF2B5	COLEC10	ASPH
	EIF3A	COLGALT2	ASPRV1
	EIF4B	COMMD10	ASRGL1
	EIF4E2	COMT	ASTL
	ELANE	COPS8	ASTN2
	ELF3	CORO2A	ASXL2
	ELMSAN1	CORO2B	ATAD2
	ELOVL2	COX6C	ATAD2B
	ELP3	COX7A2	ATAD5
	EMC10	COX7A2L	ATF2
	EMC8	CPB2	ATF3

EMP1	CPEB4	ATF6
ENAH	CPED1	ATF7
ENG	CPLX2	ATF7IP
ENGASE	CPN1	ATF7IP2
ENKUR	CPQ	ATG10
ENO1	CPSF6	ATG12
ENO1-AS1	CPT1A	ATG14
ENTPD3-AS1	CPXM2	ATG2B
ENTPD5	CRABP2	ATG7
ENTPD8	CRADD	ATIC
EPC2	CRAT	ATL1
EPCAM	CRCP	ATL2
EPHB2	CREB1	ATL3
EPN3	CREBL2	ATM
EPS8L1	CRIP2	ATMIN
EPS8L2	CRISPLD2	ATOH8
EQTN	CRKL	ATP10A
ERBB2	CRLF3	ATP11A
ERI3	CRMP1	ATP1A3
ERN1	CROT	ATP1B1
ERRFI1	CRP	ATP1B2
ESD	CRTC3	ATP1B3
ESPN	CRY1	ATP2A3
ESRP2	CRYBB3	ATP2B1
ETFDH	CRYL1	ATP2B2
ETNK2	CS	ATP2B3
EVI2A	CSDE1	ATP2B4
EVI5	CSF2RA	ATP2C2
EXD3	CSF3	ATP5EP2
EXO5	CSMD1	ATP5F1
EYA4	CSMD2	ATP5G2
FABP3	CSNK1G2	ATP6V0A2
FADD	CSRNP1	ATP6V0A4
FAHD1	CSRNP3	ATP6V0D1
FAM102A	CTAGE5	ATP6V0E1
FAM126A	CTC1	ATP6V1B1
FAM136A	CTDSPL	ATP6V1E1
FAM169B	CTIF	ATP6V1G1
FAM173A	CTLA4	ATP6V1H
FAM195A	CTR9	ATP8A1
FAM198A	CTSB	ATP8A2
FAM208B	CTSD	ATP8B2
FAM20C	CTTNBP2NL	ATP8B4
FAM227B	CTU1	ATP9B
FAM25A	CUBN	ATPAF1
FAM25C	CUEDC1	ATRN
FAM25G	CUX1	ATRNL1
FAM3B	CXCL1	ATRX
FAM63A	CXCL12	ATXN1
FAM71E1	CXCL9	ATXN10
FAM73A	CXORF40A	ATXN2
FAM81A	CXORF40B	ATXN3
FAM83B	CYP17A1	ATXN7
FAM83E	CYP1A1	AUH

FAM84B	CYP1B1	AUTS2
FAM86EP	CYP21A2	AXDND1
FAM96B	CYP26A1	AXIN1
FAT2	CYP27A1	AZGP1
FBLN1	CYP2A7	AZIN1
FBP1	CYP2B6	AZU1
FBXL16	CYP2R1	B3GALNT1
FBXL19	CYP3A5	B3GALNT2
FBXL19-AS1	CYP4A11	B3GALT1
FBXL3	CYP4F8	B3GALT2
FBXO34	CYSRT1	B3GALT5
FBXO4	CYTH3	B3GALT6
FBXO48	DAB2	B3GAT1
FBXW11	DACT2	B3GNT1
FBXW4P1	DAP	B3GNT6
FCGBP	DAPK1	B3GNTL1
FEM1A	DCAF4	B4GALNT3
FER1L6-AS1	DCC	B4GALT1
FFAR4	DCDC2	B4GALT5
FGF18	DCLK1	B4GALT6
FGF19	DCN	B9D1
FGF22	DCUN1D4	BAALC
FGF9	DDAH1	BACE2
FKBP10	DDC	BACH2
FKBP14	DDHD1	BAG1
FKBP4	DDIT3	BAG4
FLCN	DDO	BAIAP2
FLJ22447	DDX26B	BAIAP2L1
FLJ22763	DEDD	BAMBI
FLJ26245	DEFB109	BANP
FLJ40194	DENND2A	BARD1
FLJ42969	DENND2C	BARHL1
FLJ45079	DENND4C	BARHL2
FLNA	DEPTOR	BARX1
FMN1	DESI2	BARX2
FOXK1	DFFA	BASP1
FOXN1	DFNB31	BATF
FOXS1	DGKD	BAX
FREM2	DGKI	BAZ2A
FRRS1	DGUOK	BAZ2B
FSTL3	DHCR24	BBOX1
FTCDNL1	DHRS2	BBS1
FTO	DHRS3	BBS10
FUNDC1	DHRS7	BBS2
FUNDC2	DHX16	BBS4
FUT3	DHX35	BBX
FUT4	DHX40	BCAP31
FUT8	DLC1	BCAR4
FZD5	DLG5	BCAS1
G0S2	DLGAP1	BCAS3
GAA	DLL1	BCCIP
GABARAP	DMGDH	BCHE
GADD45GIP1	DNAH11	BCKDHB
GAL3ST2	DNAJB6	BCL10

GAL3ST3	DNAJC11	BCL11B
GALNS	DNAJC12	BCL2
GALNT7	DNAJC15	BCL2L1
GAMT	DNASE2B	BCL2L11
GAPDH	DNER	BCL9
GAS2L1	DNM3	BCLAF1
GAS5	DNM3OS	BCOR
GAS6	DOCK4	BCORP1
GATB	DOCK7	BCYRN1
GATC	DOCK9	BDH1
GATSL3	DOK7	BEND2
GBAS	DPAGT1	BEND5
GBE1	DPF3	BEND6
GBP5	DPP4	BEND7
GCHFR	DPP6	BFAR
GCLM	DPYSL3	BHLHA15
GCN1L1	DRD3	BHLHE22
GCNT1	DSC2	BHLHE40
GCNT2	DSCAM	BHMT2
GCOM1	DST	BICD1
GDA	DSTN	BID
GDF1	DUSP12	BIVM
GDPD1	DUSP16	BLK
GEMIN7	DUSP23	BLNK
GGA1	DUSP26	BLOC1S4
GGT1	DYM	BMF
GGT6	DYNC1I1	BMI1
GIPC1	DYNLRB2	BMP2K
GLCCI1	DYRK2	BMP4
GLI3	E2F3	BMP6
GNAS	EBAG9	BMP8B
GNB1L	ECE1	BMPR1A
GNB4	ECHDC3	BMPR1B
GNMT	ECHS1	BMPR2
GNPNAT1	ECI2	BMS1
GNPTG	ECM2	BNC1
GOLGA4	EDA2R	BNC2
GPKOW	EDEM1	BNIP3
GPR108	EDN1	BOC
GPR137	EDNRA	BOLA2
GPR153	EDNRB	BORA
GPR39	EEF1B2	BPGM
GPR83	EEF2	BPIFA3
GPRC5C	EEPD1	BPIFA4P
GPRIN1	EFCAB1	BPIFB1
GPSM1	EFCAB3	BPIFB4
GPT	EFEMP1	BPIFC
GPT2	EFHD1	BPTF
GREB1	EFNA1	BRCA2
GRHL1	EFNA5	BRD1
GRHL3	EFR3B	BRD2
GRINA	EGFL6	BRD3
GRK4	EGFLAM	BRD4
GSC2	EGFR	BRD7

GSE1	EGR2	BRD8
GSK3A	EGR3	BRDT
GSN	EI24	BRE
GSPT1	EIF2S2	BRINP3
GSTP1	EIF3C	BRIP1
GSTZ1	EIF3D	BRMS1L
GTF2F1	EIF3L	BRSK1
GTF2IRD1	EIF4EBP3	BRWD1
GTPBP2	ELF3	BTBD1
GUCA2A	ELL	BTBD17
GUCA2B	ELL2	BTBD3
GYLTL1B	ELMO1	BTBD7
GYPA	ELMSAN1	BTBD8
GYPC	ELN	BTBD9
H1FX	ELOVL6	BTC
H1FX-AS1	ELP3	BTD
H3F3A	EMB	BTF3L4
H3F3AP4	EMCN	BTG3
HAGH	EMILIN1	BTN2A2
HAUS8	EMILIN2	BTN3A3
HAVCR2	EML1	BTNL3
HBA2	EML4	BTNL9
HCN2	ENAH	BTRC
HDAC7	ENC1	BUB3
HEATR6	ENG	BVES
HEBP2	ENO1	BZW2
HERC5	ENPP2	C10ORF107
HES1	ENPP6	C10ORF11
HES2	ENSA	C10ORF113
HES3	EPAS1	C10ORF12
HES4	EPB41	C10ORF71
HHIP	EPB41L2	C10ORF88
HIST1H4B	EPC1	C10ORF90
HIST3H3	EPHA2	C11ORF24
HIVEP2	EPM2A	C11ORF40
HMBOX1	EPRS	C11ORF48
HMGA1P7	EPS15	C11ORF49
HMGXB3	EPS15L1	C11ORF53
HMHA1	ERC1	C11ORF57
HMOX2	ERC2	C11ORF63
HNRNPH3	ERCC6L2	C11ORF68
HOGA1	ERLIN2	C11ORF70
HOMER2	ERMP1	C11ORF80
HOXA7	ERN1	C11ORF95
HOXB4	ERVW-4	C12ORF29
HOXB5	ESAM	C12ORF42
HOXB7	ESR1	C12ORF56
HOXC10	ESR2	C12ORF57
HPN	ETFA	C12ORF65
HR	ETFB	C12ORF71
HSD11B1L	ETL4	C12ORF74
HSD17B1	ETNK2	C12ORF76
HSD17B4	ETNPPL	C14ORF105
HSF1	EVL	C14ORF132

HSPB1	EXOC4	C14ORF159
HSPB8	EXOSC2	C14ORF166
HSPB9	EXOSC8	C14ORF2
HUS1	EXT1	C14ORF80
HUWE1	EYA2	C15ORF27
ICOSLG	F11R	C15ORF32
ID1	F12	C15ORF40
ID4	F2R	C15ORF41
IER3	F3	C15ORF43
IER5L	FAAH	C15ORF48
IFRD1	FABP9	C15ORF54
IFRD2	FADS2	C16ORF45
IFT20	FAH	C16ORF54
IFT81	FAHD2A	C16ORF72
IGFBP2	FAIM	C16ORF90
IGFN1	FAIM2	C17ORF53
IKBKG	FAM102B	C17ORF58
IKZF2	FAM110C	C17ORF67
IL15	FAM114A1	C17ORF70
IL17RB	FAM117B	C17ORF82
IL19	FAM129A	C18ORF54
IL1R2	FAM160A1	C19ORF45
IL20	FAM160B1	C19ORF47
IL24	FAM163A	C19ORF48
IL27	FAM174B	C1D
IL6ST	FAM189A2	C1GALT1
INADL	FAM20B	C1ORF101
INPP5A	FAM213A	C1ORF110
INPP5F	FAM219A	C1ORF115
INTS10	FAM3B	C1ORF116
INTS5	FAM49A	C1ORF168
INTS9	FAM53B	C1ORF189
IP6K3	FAM69A	C1ORF198
IPPK	FAM84B	C1ORF21
IQCD	FAM86C1	C1ORF43
IQCE	FANCD2OS	C1ORF54
ISPD	FAP	C1ORF87
ISYNA1	FARP1	C1ORF94
ITGB2	FAXDC2	C1QBP
ITGB6	FBF1	C1QTNF3
ITGB8	FBLN2	C1QTNF7
ITPK1	FBN2	C20ORF85
ITPR1	FBXL7	C21ORF33
ITSN2	FBXO31	C21ORF59
IVL	FBXO32	C22ORF24
JAK1	FBXO7	C22ORF34
JAZF1	FBXW7	C22ORF42
JAZF1-AS1	FBXW8	C2CD2
JDP2	FCER1G	C2CD4A
JPH2	FCGRT	C2CD5
JUND	FER	C2ORF48
KALRN	FERMT1	C2ORF68
KANK2	FEZ2	C2ORF76
KAT2A	FGB	C2ORF88

KAT6B	FGD4	C3ORF20
KBTBD4	FGD6	C3ORF30
KBTBD6	FGF18	C3ORF58
KCNA2	FGF2	C3ORF79
KCNC4	FGF9	C4ORF22
KCNE3	FGG	C4ORF3
KCNF1	FGGY	C4ORF33
KCNH8	FGL1	C4ORF36
KCNIP4	FHIT	C4ORF45
KCNJ1	FHL2	C4ORF47
KCNJ5	FKBP4	C5ORF15
KCNK15	FLNB	C5ORF24
KCNK6	FLRT2	C5ORF28
KCNQ5	FLT4	C5ORF30
KCTD11	FLVCR2	C5ORF42
KCTD15	FMNL1	C5ORF45
KCTD18	FMNL2	C5ORF46
KCTD20	FMO2	C6ORF201
KCTD6	FMOD	C6ORF222
KDM4B	FN1	C6ORF58
KEAP1	FNBP1	C6ORF89
KIAA0087	FNDC1	C7ORF25
KIAA1407	FNDC3A	C7ORF26
KIAA1598	FNDC3B	C7ORF60
KIAA1683	FNDC4	C8B
KIF21A	FNTB	C8ORF31
KLC1	FOS	C8ORF4
KLF10	FOSL2	C8ORF46
KLF13	FOXB1	C9ORF106
KLF15	FOXD2	C9ORF152
KLF6	FOXJ3	C9ORF3
KLF9	FOXK1	C9ORF64
KLHDC7A	FOXN1	C9ORF69
KLHDC8A	FOXO1	C9ORF85
KLHL21	FOXP1	C9ORF89
KLHL33	FRAT1	CA10
KLK13	FRAT2	CA12
KLRC2	FREM2	CA13
KLRC3	FRG1	CA4
KLRF2	FRMD4B	CA6
KMT2D	FRMD6	CA8
KRT13	FRZB	CAAP1
KRT18	FSIP1	CABIN1
KRT23	FSTL1	CABLES1
KRT4	FSTL4	CACHD1
KRT73	FUNDC1	CACNA1A
KRT8	FXN	CACNA1B
KRT80	FXR1	CACNA1C
KRTAP1-5	FXYD6	CACNA1D
KRTAP10-4	FXYD7	CACNA1G
KRTAP2-1	FYN	CACNA1S
KRTAP2-2	FZD8	CACNA2D1
KRTAP3-1	G6PC	CACNA2D3
KRTAP3-2	GAB2	CACNA2D4

KRTAP4-7	GADD45A	CACNB2
KRTAP4-8	GAL3ST1	CACNB3
KRTAP4-9	GAL3ST4	CACNG1
LAMA5	GALM	CACNG2
LAMB3	GALNT11	CACNG3
LAMC1	GALNT15	CACNG5
LAMTOR2	GALNT16	CACNG6
LARP4	GALNT18	CACNG7
LARS2	GALNT7	CADPS
LASP1	GALNTL6	CADPS2
LATS1	GAREM	CALB1
LCA5L	GAS2	CALB2
LCN12	GAS5	CALD1
LCN2	GAS6	CALM1
LCN8	GAS7	CALM2
LDB1	GATA1	CALML4
LDB3	GATA6	CALML5
LDLRAD3	GATAD2B	CALN1
LETM1	GBE1	CAMK1
LGI1	GC	CAMK1G
LHCGR	GCAT	CAMK2B
LHX4	GCLC	CAMK2D
LINC00111	GCLM	CAMK2N2
LINC00167	GDE1	CAMKK2
LINC00173	GDF10	CAMKMT
LINC00208	GDF15	CAMP
LINC00235	GDF5	CAMSAP2
LINC00273	GEMIN5	CAMTA1
LINC00336	GFOD1	CAP2
LINC00482	GFRA1	CAPG
LINC00483	GGT5	CAPN1
LINC00511	GHR	CAPN10
LINC00574	GINS3	CAPN13
LINC00596	GJB1	CAPN14
LINC00862	GK2	CAPN3
LINC01553	GLA	CAPN7
LINC01555	GLB1L2	CAPN9
LINC01559	GLCCI1	CAPRIN1
LITAF	GLCE	CAPS2
LMBR1	GLI2	CAPZB
LMCD1	GLI3	CARD11
LMX1B	GLIS1	CARD18
LOC100128361	GLIS3	CASC10
LOC100128531	GLRB	CASC3
LOC100129534	GMFB	CASC5
LOC100129931	GNA14	CASK
LOC100130238	GNE	CASP2
LOC100130264	GNG2	CASQ2
LOC100130417	GNGT1	CASZ1
LOC100130691	GNMT	CATSPER3
LOC100130880	GNPNAT1	CATSPERB
LOC100131496	GNS	CATSPERD
LOC100132146	GOLPH3L	CAV1
LOC100133669	GOT1	CAV2

LOC100506023	GOT2	CAV3
LOC100506469	GPAM	CBFA2T2
LOC100506746	GPC6	CBFA2T3
LOC100507217	GPD2	CBL
LOC100630918	GPHN	CBLB
LOC151484	GPLD1	CBLC
LOC283332	GPM6B	CBLL1
LOC284344	GPR149	CBLN2
LOC284454	GPR156	CBLN4
LOC284865	GPR157	CBR4
LOC286190	GPR22	CBWD2
LOC344887	GPR37L1	CBWD5
LOC389033	GPR39	CBX2
LOC399715	GPR65	CBX4
LOC400548	GPR83	CBX8
LOC440028	GPRC5C	CBY1
LOC553103	GPRC5D	CBY3
LOC645513	GPRIN3	CC2D1A
LOC652276	GPT	CC2D1B
LOC727710	GPT2	CC2D2A
LOC728392	GPX3	CCAR2
LOC730102	GPX4	CCBE1
LOC730668	GPX7	CCBL2
LONRF2	GRAMD1A	CCDC109B
LOX	GRAMD1B	CCDC110
LOXL4	GRAMD3	CCDC112
LPAR1	GREB1	CCDC127
LPCAT3	GREM2	CCDC129
LPIN1	GRHPR	CCDC141
LPIN2	GRIA4	CCDC155
LRFN3	GRIK3	CCDC171
LRG1	GRIK4	CCDC172
LRP5L	GRM4	CCDC174
LRP6	GSE1	CCDC178
LRRC10B	GSTA1	CCDC18
LRRC15	GSX1	CCDC181
LRRC17	GTF2IRD2	CCDC183
LRRC31	GTF3C4	CCDC186
LRRC52	GUCA2B	CCDC23
LRRC58	GUCY2GP	CCDC33
LRRC8B	GULP1	CCDC34
LRSAM1	GXYLT2	CCDC38
LRTOMT	GYPC	CCDC42
LRWD1	H2AFZ	CCDC51
LSM14A	H3F3B	CCDC53
LTBP3	HABP2	CCDC54
LTBR	HAGH	CCDC57
LUC7L	HAL	CCDC6
LY6E	HAND2	CCDC74A
LYPLAL1	HBEGF	CCDC81
LYRM1	HCAR1	CCDC85C
LYRM9	HDAC11	CCDC88A
LYSMD1	HDAC4	CCDC88B
LZTS3	HDAC7	CCDC88C

MACC1	HDAC8	CCDC93
MAD2L1BP	HERC1	CCER1
MAF1	HES1	CCHCR1
MAFA	HEY2	CCK
MAGEA11	HFE2	CCKAR
MALAT1	HGD	CCL1
MALL	HHAT	CCL20
MALSU1	HIBADH	CCL27
MAMSTR	HIF1A	CCL3L1
MAN2C1	HIPK1	CCL4
MANEAL	HIST1H2BG	CCL7
MAP2K7	HIST2H2AB	CCNA1
MAP3K12	HIST2H4A	CCNA2
MAP3K8	HIST3H3	CCND1
MAP6D1	HIVEP1	CCND2
MAPK13	HIVEP2	CCNE1
MAPK9	HMBOX1	CCNE2
MARCH5	HMCES	CCNF
MARVELD2	HMCN2	CCNH
MAST2	HMGCL	CCNJ
MAT1A	HMGCLL1	CCNJL
MAT2A	HMGN3	CCNL1
MAU2	HNF1A	CCNL2
MB	HNF4A	CCNY
MCAT	HNRNPLL	CCP110
MCEE	HOMER1	CCPG1
MCF2L	HOXA10	CCR3
MCF2L-AS1	HOXB3	CCR7
MCM8	HOXC10	CCR9
MCTS2P	HOXC13	CCSER1
MDM2	HOXC6	CCSER2
ME3	HOXD8	CCT6B
MED13L	HPGD	CCT6P3
MED4	HRAS	CCZ1
MEF2A	HRH1	CCZ1B
MEOX1	HS3ST3A1	CD163L1
METAP1D	HS3ST5	CD164
METRNL	HS6ST2	CD180
METTL7B	HSD11B2	CD1B
MFGE8	HSD17B11	CD1E
MFI2-AS1	HSD17B2	CD200R1L
MFSD12	HSD17B8	CD209
MFSD2A	HSF2	CD22
MFSD3	HSF2BP	CD226
MFSD7	HSPA12B	CD24
MGP	HSPB8	CD244
MIB2	HTR2A	CD2BP2
MICALCL	HTRA1	CD300E
MID1	HUNK	CD34
MIEF2	HYAL1	CD36
MIEN1	HYAL3	CD38
MIER1	ICAM3	CD3E
MINOS1	ICAM4	CD40LG
MINOS1-NBL1	ICAM5	CD46

MIOS	ID2	CD55
MIR1227	ID3	CD58
MIR1250	ID4	CD63
MIR135B	IDH1	CD69
MIR149	IER2	CD7
MIR190B	IER3	CD70
MIR196A2	IFNAR2	CD79B
MIR196B	IFNK	CD82
MIR2117	IFRD2	CD83
MIR23A	IFT122	CD86
MIR23B	IGDCC4	CD9
MIR24-1	IGF1	CD93
MIR24-2	IGF2R	CD96
MIR27A	IGFBP1	CD99
MIR27B	IGFBP3	CDC14B
MIR3074	IGFBP4	CDC25A
MIR30A	IGFBP7	CDC27
MIR3148	IGSF10	CDC34
MIR3155A	IGSF3	CDC42BPA
MIR3155B	IGSF8	CDC42EP3
MIR3158-1	IKBKB	CDC42EP4
MIR3158-2	IKBKG	CDC45
MIR3188	IKZF2	CDC7
MIR3193	IL12B	CDCA2
MIR326	IL17D	CDCA7
MIR3614	IL17RA	CDCA7L
MIR3622A	IL17RB	CDCA8
MIR3622B	IL17RD	CDH1
MIR3648-1	IL1R1	CDH10
MIR3653	IL1R2	CDH11
MIR365B	IL33	CDH12
MIR3687-1	IL6ST	CDH13
MIR3926-1	ILF3	CDH15
MIR3926-2	IMMP2L	CDH18
MIR4252	IMP4	CDH19
MIR4258	INADL	CDH2
MIR4261	INF2	CDH22
MIR4429	INHBB	CDH23
MIR4440	INMT	CDH24
MIR4446	INSIG2	CDH26
MIR4469	INTU	CDH4
MIR4472-2	IPO13	CDH5
MIR4478	IPO5	CDH6
MIR4488	IQCA1	CDH7
MIR4505	IQSEC3	CDH9
MIR4513	IRAK2	CDHR2
MIR4519	IRF2BP2	CDHR3
MIR4530	IRF2BPL	CDHR5
MIR4633	IRF4	CDK10
MIR4654	IRF8	CDK13
MIR4669	IRS2	CDK14
MIR4674	IRX5	CDK18
MIR4681	ISG20	CDK19
MIR4692	ISLR	CDK2

MIR4698	ISOC1	CDK2AP1
MIR4715	ITFG2	CDK5R1
MIR4725	ITGA10	CDK5RAP2
MIR4729	ITGA2	CDK7
MIR4734	ITGA9	CDK8
MIR4738	ITGB1	CDKAL1
MIR4761	ITGB3	CDKL1
MIR4774	ITGB5	CDKL2
MIR548J	ITIH1	CDKN2B
MIR550A1	ITIH2	CDKN2B-AS1
MIR550A2	ITM2C	CDON
MIR550A3	ITPR1	CDS1
MIR550B1	JAG1	CDS2
MIR550B2	JAK2	CDV3
MIR554	JARID2	CDY2B
MIR567	JAZF1	CDYL
MIR589	JPH4	CDYL2
MIR623	JUNB	CEACAM4
MIR661	KALRN	CEACAM6
MIR663A	KANK1	CEBPZ
MIR7-3	KAT2B	CECR5-AS1
MIR7-3HG	KAT6B	CECR6
MIRLET7BHG	KATNAL1	CELF4
MISP	KAZN	CELSR1
MLLT6	KCNA1	CELSR2
MLPH	KCNE4	CEMIP
MLXIPL	KCNG4	CENPE
MMRN2	KCNH2	CENPF
MN1	KCNK2	CENPJ
MNT	KCNMA1	CENPK
MOB1A	KCNMB1	CENPL
MOB2	KCNQ5	CENPN
MPC1	KCNT2	CENPO
MPC2	KCTD15	CENPP
MPHOSPH10	KCTD3	CENPW
MPST	KCTD4	CEP112
MRPL24	KDM2A	CEP131
MRPS26	KDM7A	CEP135
MS4A15	KDR	CEP164
MS4A7	KHDRBS3	CEP170
MS4A8	KHK	CEP170B
MSMB	KIAA0513	CEP192
MSMO1	KIAA0922	CEP350
MT1B	KIAA1024	CEP44
MT2A	KIAA1024L	CEP55
MTDH	KIAA1211L	CEP57L1
MTG1	KIAA1551	CEP83
MTHFD1L	KIAA1614	CEP89
MTMR4	KIAA2022	CERS3
MTSS1L	KIF1B	CERS6
MUC1	KIF26B	CES3
MUC2	KIF3B	CETN3
MUC5B	KIF5C	CETN4P
MUM1	KIFAP3	CFAP43

MXD3	KIFC1	CFAP46
MXRA5	KIFC3	CFAP47
MYB	KIRREL3	CFAP54
MYBPC2	KLC1	CFAP58
MYEOV	KLF10	CFAP61
MYL6	KLF2	CFAP69
MYL6B	KLF3	CFAP70
MYLIP	KLHL26	CGNL1
MYO10	KLKB1	CHAF1B
MYO9B	KMO	CHAT
MYOF	KNG1	CHD1
MYOG	KPNA2	CHD2
MYOT	KRAS	CHD3
MYZAP	KRR1	CHD6
NAA15	KRT13	CHD9
NAA38	KRT15	CHERP
NAALADL2	KRT18	CHIA
NAB2	KRT19	CHKA
NADSYN1	KRT23	CHL1
NAIP	KRT78	CHML
NAP1L4	KRT8	CHMP1B
NAT14	KRT86	CHMP2A
NAT8	KSR1	CHMP2B
NAT8B	KSR2	CHMP4C
NBN	KXD1	CHMP7
NBPF1	KYNU	CHN2
NBR1	LAMA4	CHORDC1
NCALD	LAMA5	CHP1
NCCRP1	LAMB1	CHPF
NCMAP	LAPTM4A	CHPF2
NCOA4	LAPTM4B	CHRDL1
NDC1	LARGE	CHRM3
NDST1	LARS	CHRNA2
NDUFC1	LAX1	CHRNA3
NDUFS3	LBH	CHRNA5
NDUFV2	LBP	CHRNA7
NEAT1	LCAT	CHRNB3
NECAB3	LCLAT1	CHRND
NEDD9	LCN2	CHST1
NEFH	LCOR	CHST10
NEGR1	LDAH	CHST11
NEK6	LDB2	CHST13
NEURL3	LDLRAD4	CHST4
NFKB1	LEAP2	CHST8
NFKBIA	LECT2	CHST9
NHLRC4	LEF1	CHSY1
NIPAL3	LEKR1	CHSY3
NIT1	LEMD1	CIDEA
NMRAL1	LEP	CIRH1A
NMT1	LEPR	CISH
NNT	LGALS8	CIT
NOC4L	LGALS9	CITED1
NOMO1	LGALSL	CITED2
NOMO2	LGI4	CITED4

NOMO3	LGR6	CKAP2L
NOP14	LHFPL2	CKAP5
NOP14-AS1	LHX6	CKM
NOTCH1	LIFR	CKMT1B
NOTUM	LIMA1	CLASP1
NOXA1	LIMD1	CLASP2
NPAS4	LIMS1	CLASRP
NPR3	LIMS2	CLC
NPRL2	LIPC	CLCA3P
NR1I3	LITAF	CLCN7
NR2F6	LMAN2L	CLDN1
NR4A3	LMCD1	CLDN10
NRAP	LMNTD1	CLDN12
NRARP	LMOD3	CLDN14
NRCAM	LNPEP	CLDN15
NRDE2	LNX1	CLDN3
NRG4	LNX2	CLDN8
NRIP1	LOC145837	CLEC11A
NRP1	LOC284379	CLEC16A
NRP2	LOC284912	CLEC4E
NSUN3	LOC285696	CLEC4G
NT5C2	LOC441956	CLEC4M
NT5DC3	LONP2	CLEC7A
NTAN1	LONRF1	CLIC5
NTHL1	LOXL2	CLIP1
NUAK1	LPP	CLIP2
NUBP2	LRCH1	CLIP4
NUCB1	LRCH3	CLMN
NUDT14	LRFN2	CLN8
NUDT16P1	LRG1	CLNK
NUDT3	LRIG1	CLOCK
NUMA1	LRIG3	CLPB
NUTF2	LRP1	CLPP
NWD1	LRP4	CLPTM1L
NXNL2	LRPPRC	CLPX
NYX	LRRC17	CLRN1-AS1
OBP2B	LRRC49	CLTA
OCA2	LRRC52	CLTB
ODF3	LRRC61	CLTC
OLFML2A	LRRC69	CLU
OLFML3	LRRC8E	CLUAP1
OLIG1	LRTM1	CLUH
OLR1	LSM10	CLVS2
OPHN1	LSM14A	CLYBL
OR13C2	LSM4	CMBL
OR2F2	LTA4H	CMIP
OR5AP2	LTBP1	CMKLR1
ORC6	LTF	CMPK2
ORMDL3	LUM	CMSS1
OTUD1	LUZP1	CMTM3
OVOL2	LY6G6E	CMTM8
P2RX6P	LY86	CNBD1
P2RX7	LYN	CNEP1R1
P4HA2	LYPLAL1	CNGA1

PA2G4	LYST	CNGA3
PA2G4P4	LZTS2	CNGB3
PABPC1	MAB21L2	CNIH3
PADI3	MACF1	CNKSR3
PALMD	MAF	CNN3
PANK4	MAGI1	CNNM1
PARL	MALL	CNOT2
PARP4	MAMDC2	CNOT4
PARVA	MAML2	CNOT6L
PBLD	MAML3	CNPY2
PCBD2	MAN1A1	CNR1
PCDH1	MAOA	CNRIP1
PCM1	MAP2K4	CNTFR
PCYT1A	MAP2K6	CNTLN
PCYT2	MAP2K7	CNTN1
PDCD10	MAP3K5	CNTN3
PDCD6IP	MAP3K7	CNTN4
PDCL3	MAP4	CNTN5
PDE6B	MAP4K4	CNTNAP2
PDE7A	MAP6	CNTNAP4
PDF	MAP6D1	CNTNAP5
PDLIM1	MAPK14	COA1
PDZD4	MAPKAP1	COBL
PDZK1	MAPKAPK2	COBLL1
PEMT	MAPT	COG1
PERM1	MARK1	COG2
PERP	MARK2	COG3
PFDN2	MARVELD1	COG6
PGAP3	MAST4	COL10A1
PGLYRP2	MASTL	COL12A1
PGPEP1	MAT1A	COL18A1
PGR	MAX	COL21A1
PGS1	MB	COL22A1
PHACTR1	MBD3L2	COL23A1
PHACTR4	MBNL1	COL24A1
PHF12	MBNL2	COL25A1
PHF13	MBP	COL26A1
PHF23	MC2R	COL27A1
PHGR1	MCM3	COL28A1
PI16	MCTP2	COL4A1
PIGP	MCU	COL4A2
PIKFYVE	MCUR1	COL4A3BP
PIM3	MDFIC	COL4A5
PIP	MDM2	COL5A1
PIPOX	ME3	COL5A2
PISD	MED12L	COL6A1
PKD2L2	MED13L	COL6A3
PKM	MED24	COL8A1
PKP1	MED4	COL9A1
PKP2	MEF2C	COLCA2
PLA1A	MEGF11	COLEC11
PLA2G6	MEGF9	COLGALT2
PLAC1	MEIS1	COLQ
PLAT	METTL24	COMMD10

PLCB1	METTL2A	COMMD4
PLCE1	METTL7A	COMMD6
PLD1	MEX3C	COMMD8
PLEC	MFGE8	COMMD9
PLEKHA1	MFN1	COPB1
PLEKHA2	MGA	COPS7A
PLEKHA8	MGAT5	COPS7B
PLEKHD1	MGEA5	COPS8
PLEKHG3	MGMT	COQ10B
PLEKHH1	MGST1	COQ7
PLEKHH3	MICAL2	CORIN
PLEKHJ1	MICAL3	CORO2A
PLIN5	MICALL1	CORO2B
PLS1	MICB	CORO6
PLS3	MIDN	CORT
PLXNB2	MINOS1	
PMAIP1	MITF	COX20
PMEPA1	MKL1	COX5B
PMM2	MKLN1	COX6A2
PNKD	MKNK2	COX7B2
PNMT	MLF2	CP
PNPLA2	MLPH	CPAMD8
PNRC2	MLXIPL	CPB1
POGK	MMD	CPB2
POLI	MME	CPEB2
POLRMT	MMP19	CPEB3
POMT2	MOB3B	CPEB4
PORCN	MOCS1	CPLX2
POU2F3	MOCS2	CPLX4
PP14571	MORN3	CPNE1
PPL	MOV10	CPNE2
PPM1J	MOXD1	CPNE4
PPM1M	MPDZ	CPNE5
PPP1R12C	MPP6	CPNE8
PPP1R13L	MPPED1	CPO
PPP1R14A	MPZL2	CPPED1
PPP2R1A	MR1	CPQ
PPP4C	MRC2	CPSF7
PPP4R1	MREG	CPT1A
PPRC1	MRFAP1	CPXCR1
PPT1	MRPL19	CPZ
PQLC2L	MRPL22	CRABP2
PRCC	MRPL33	CRACR2A
PRDM10	MRPL43	CRADD
PRDX6	MRPS18A	CRB2
PRKACA	MRPS22	CRBN
PRKAG2	MRVI1	CRCP
PRKCD	MS4A7	CREB3L1
PRL	MTA1	CREB5
PRPS1	MTCL1	CREBBP
PRR15L	MTDH	CREG2
PRR18	MTHFD1	CRELD2
PRR34	MTHFD1L	CRH
PRR35	MTNR1B	CRHR1

PRSS22	MTSS1	CRHR1-IT1
PRSS27	MTUS1	CRIM1
PRSS37	MUSK	CRISP2
PRUNE	MYB	CRISP3
PSEN2	MYBBP1A	CRISPLD1
PTGDS	MYBPC1	CRISPLD2
PTGR1	MYCBPAP	CRKL
PTGR2	MYD88	CRNN
PTK6	MYEOV	CROT
PTMA	MYF6	CRTAM
PTP4A2	MYH10	CRTAP
PTPN13	MYH13	CRTC1
PTPRF	MYH9	CRY1
PTPRO	MYL12B	CRYAA
PTTG1IP	MYO16	CRYBG3
PUM1	MYO1E	CRYZL1
PVRL2	MYO3A	CSAD
PWWP2B	MYO5B	CSF1
PXK	MYO5C	CSF1R
PXMP4	MYO6	CSGALNACT1
PXT1	MYOCD	CSMD1
QPRT	MYOF	CSMD2
QSOX1	MYOG	CSMD3
QSOX2	MYOM2	CSNK1A1
QTRTD1	MYOT	CSNK1G3
RAB18	MYRIP	CSNK2A1
RAB26	MYT1L	CSPG4
RAB40C	N4BP3	CSRNP1
RAB5C	N6AMT1	CSRP2
RABL6	NAGLU	CST8
RAD23B	NAGS	CSTF2T
RAD54L	NARS	CT45A6
RALGAPA2	NAT2	CT55
RALGPS2	NAT6	CTAGE1
RALY	NAV1	CTAGE4
RANBP1	NAV2	CTAGE6
RANBP10	NAV3	CTBP1
RANBP17	NBEA	CTBP2
RANGAP1	NBN	CTGF
RAP2A	NBPF1	CTIF
RAPGEF3	NBPF10	CTNNA1
RAPGEF5	NBPF14	CTNNA2
RARA	NBPF15	CTNNA3
RARRES2	NBPF20	CTNNAL1
RASA3	NBPF3	CTNNB1
RASEF	NBPF8	CTNNBIP1
RASGEF1B	NCKAP5	CTNNBL1
RAVER1	NCOA2	CTNND1
RBBP7	NCOA7	CTNND2
RBBP8NL	NCOR2	CTPS1
RBL2	NDRG1	CTSC
RBM6	NDRG2	CTSG
RBMS3	NDST1	CTSL3P
RBP5	NDUFA4	CTSW

RCOR3	NDUFS1	CTTNBP2
RDH13	NDUFS2	CTTNBP2NL
RDM1	NEDD4L	CTXN3
RDX	NEGR1	CUL1
REEP4	NEIL2	CUL2
RERG	NEK10	CUL3
RFESD	NEK7	CUL5
RFPL1S	NEK8	CUX1
RFX2	NEK9	CUX2
RFX4	NELL1	CWC22
RGL2	NFATC2	CWC25
RGL3	NFE2L1	CWF19L2
RGPD6	NFE2L2	CX3CR1
RGS10	NFIC	CXCL1
RGS4	NFIL3	CXCL2
RGS8	NFKB1	CXCL5
RHOBTB3	NFKBIA	CXCL8
RHOC	NGEF	CXCR6
RHOD	NHEJ1	CXORF21
RHOF	NHS	CXORF23
RHOV	NID2	CXORF51A
RIPK4	NKAIN1	CXORF57
RND3	NKAIN3	CXORF58
RNF144B	NKD2	CYB5A
RNF157	NKIRAS1	CYB5R3
RNF19A	NLN	CYB5R4
RNF223	NLRP3	CYBA
RNF224	NME1	CYBRD1
RNH1	NME8	CYC1
RNU11	NMNAT2	CYFIP2
ROBO1	NMUR2	CYLC1
ROBO3	NNMT	CYLC2
RPGRIP1L	NOG	CYMP
RPL12	NOTCH2NL	CYP19A1
RPL18A	NPAS2	CYP1A1
RPL29	NPLOC4	CYP1A2
RPL8	NQO1	CYP1B1-AS1
RPLP2	NQO2	CYP24A1
RPRM	NR0B2	CYP26B1
RPS20	NR1H4	CYP2A6
RPS6KL1	NR1I3	CYP2J2
RRAS	NR2F6	CYP2S1
RRN3P3	NR3C1	CYP2U1
RSPH14	NRAP	CYP3A43
RSPH4A	NRG1	CYP3A7
RTFDC1	NRIP1	CYP46A1
RTKN	NRXN3	CYP4A11
S100A10	NSMCE1	CYP4B1
S100A11	NSMF	CYP4F22
SAMD9	NT5C1B	CYP4V2
SCAF1	NT5C2	CYP7B1
SCAMP4	NT5E	CYTH1
SCARNA16	NT5M	CYTH3
SCD	NTF3	DAAM1

SCGB1D2	NTM	DAAM2
SCGB2A1	NTN1	DAB1
SCNM1	NUAK1	DAB2IP
SCNN1A	NUDT12	DACH1
SCP2	NUDT7	DACH2
SCRIB	NUP155	DACT1
SDK2	NUP210	DACT2
SDPR	NUPL1	DACT3
SEC14L1	NVL	DAGLA
SEC31A	NXN	DAOA
SEH1L	NXPH3	DAPK1
SEMA3B	OAF	DAPL1
SEMA3C	ODF1	DARS
SEPT8	OFCC1	DAZAP1
SEPT9	OGN	DBH
SERBP1	OLFML1	DBI
SERPINA1	OLFML2A	DBN1
SERPINA6	ONECUT3	DBNDD1
SERPINI1	OPA3	DBR1
SERTAD2	OPCML	DBT
SETD2	OPTN	DBX2
SETD6	OR13C8	DCAF11
SF3A2	OR6B3	DCAF6
SF3B2	ORAOV1	DCAF8L1
SFMBT1	OSBPL1A	DCANP1
SFN	OSBPL7	DCC
SFXN2	OSGIN1	DCD
SGCA	OTOS	DCDC2
SGK3	OVGP1	DCK
SGMS2	OXA1L	DCLK2
SGOL1	OXT	DCLRE1B
SH2B3	OXTR	DCN
SH3BGR	P2RX4	DCTN6
SH3RF2	P2RY14	DCUN1D1
SHARPIN	P2RY2	DCUN1D3
SHC1	P3H2	DCUN1D4
SHMT1	PABPC1	DCUN1D5
SIAH2	PADI1	DDAH1
SIDT2	PADI2	DDB1
SIK1	PAG1	DDC
SIPA1L3	PAH	DDHD1
SIRT5	PAK2	DDI2
SKA1	PALLD	DDIT4
SKIL	PALM2	DDO
SLC10A3	PAN3	DDX10
SLC11A2	PANK1	DDX17
SLC17A9	PANK2	DDX18
SLC22A5	PANK3	DDX31
SLC25A1	PAPD5	DDX39A
SLC25A10	PAPPA2	DDX3X
SLC25A24	PAPSS1	DDX47
SLC25A25	PARD3	DDX49
SLC25A30	PARD6B	DDX52
SLC25A36	PARP16	DDX59

SLC25A42	PARP8	DDX60
SLC25A45	PARVA	DEDD2
SLC26A1	PAWR	DEFA1
SLC29A1	PBX1	DEFB114
SLC34A2	PCCA	DEFB121
SLC35F6	PCK1	DEFB128
SLC37A3	PCMT1	DEFB134
SLC38A9	PCNX	DENND1A
SLC39A10	PCNXL2	DENND5B
SLC43A2	PCNXL4	DENR
SLC44A1	PCOLCE2	DEPDC1
SLC45A4	PCP4L1	DEPDC1B
SLC46A2	PCSK4	DEPDC7
SLC47A2	PCSK5	DERL1
SLC4A2	PCSK6	DES
SLC4A7	PCTP	DEXI
SLC6A12	PCYT1A	DFNB31
SLC6A13	PDCD10	DGCR14
SLC7A1	PDCD1LG2	DGCR2
SLC7A5	PDCL3	DGKB
SLC7A5P1	PDDC1	DGKD
SLC7A5P2	PDE10A	DGKG
SLC7A6	PDE11A	DGKI
SLC9A8	PDE2A	DHDDS
SLCO4A1	PDE3A	DHDH
SLFN5	PDE4C	DHFR
SLIT3	PDE4D	DHODH
SLITRK4	PDE7B	DHRS1
SLPI	PDE8B	DHRS12
SMCR5	PDHA1	DHRS3
SMG7	PDK4	DHRS4L1
SMG7-AS1	PDP2	DHTKD1
SMPD3	PDZK1	DHX15
SNAI1	PDZRN3	DHX33
SNCG	PELI2	DHX34
SNHG11	PELO	DHX36
SNHG20	PELP1	DIAPH2
SNORA11B	PEMT	DIAPH3
SNORA11C	PER1	DICER1
SNORA52	PEX14	DIO2
SNORA71E	PEX26	DIO3
SNORA76C	PFKFB3	DIP2B
SNORD104	PGF	DIP2C
SNORD125	PGM5	DIRAS2
SNORD1A	PGPEP1	DIRAS3
SNORD1B	PGR	DIRC3
SNORD54	PGRMC1	DIS3L2
SNORD60	PGRMC2	DISC1
SNORD74	PGS1	DISP1
SNORD75	PHACTR1	DKFZP434H168
SNORD76	PHAX	DKK3
SNTB1	PHC2	DKK4
SNX14	PHF14	DLD
SNX19	PHF21B	DLEC1

SNX9	PHF5A	DLEU2
SP1	PHLDB1	DLEU7
SPATA13	PHLPP1	DLG2
SPATA2	PI15	DLG5
SPATA2L	PI4K2A	DLGAP1
SPC24	PICALM	DLK1
SPIB	PID1	DLL1
SPIRE2	PIGB	DMBT1
SPNS1	PIGP	DMC1
SPON1	PIK3AP1	DMD
SPPL2A	PIK3CB	DMRTA1
SPRY2	PIM1	DMXL1
SPSB3	PIM3	DMXL2
SPTBN4	PIP	DNAAF2
SPTBN5	PISD	DNAAF5
SRCAP	PITPNB	DNAH1
SRD5A3	PITRM1	DNAH11
SRI	PITX2	DNAH14
SRMS	PJA2	DNAH17
SRSF3	PKDCC	DNAH2
SSPN	PKIB	DNAH5
SSR3	PKIG	DNAH6
SSR4P1	PKN1	DNAH7
SSTR2	PKNOX1	DNAH8
ST3GAL2	PKNOX2	DNAH9
ST6GALNAC2	PLA2G6	DNAI2
ST8SIA4	PLAC1	DNAJA3
ST8SIA6	PLAC8	DNAJB12
STARD8	PLCE1	DNAJB3
STAT5B	PLCL1	DNAJB4
STAU1	PLD1	DNAJB9
STC2	PLEKHA2	DNAJC1
STK25	PLEKHA5	DNAJC12
STKLD1	PLEKHA6	DNAJC15
STPG1	PLEKHA7	DNAJC17
STRADB	PLEKHA8	DNAJC19
STRBP	PLEKHB2	DNAJC28
STS	PLEKHF1	DNAJC5B
STX19	PLEKHG1	DNAJC6
STX4	PLEKHG3	DNAJC9
STXBP2	PLEKHH2	DNAL1
SUGP1	PLG	DNASE1
SUGT1P3	PLIN2	DNASE2B
SULT1A2	PLK3	DNER
SULT1C2	PLOD2	DNM1L
SULT2B1	PLRG1	DNM3
SUMO1P3	PLSCR1	DNMBP
SUMO3	PLSCR2	DNMT1
SUPT3H	PLSCR4	DNMT3A
SURF4	PLXNC1	DNMT3L
SUSD2	PMCH	DNTTIP2
SWT1	PMEPA1	DOCK1
SYDE2	PMPCA	DOCK2
SYNDIG1	PNOC	DOCK4

SYNE4	PNPLA7	DOCK5
SYNGR4	PODXL	DOCK7
SYNJ2	POLR1D	DOCK8
SYT12	POLR2E	DOK2
SYT17	POLR3B	DOK6
SYT5	POLRMT	DOLPP1
SYT8	POMGNT2	DOPEY1
TACSTD2	PON1	DOPEY2
TAGLN2	POPDC3	DPAGT1
TANK	POSTN	DPEP3
TARBP2	POTEB	DPF2
TAS1R3	PPARA	DPH5
TAX1BP1	PPARGC1A	DPP10
TBC1D1	PPCDC	DPP4
TBC1D26	PPFIBP1	DPP6
TBC1D30	PPIA	DPP9
TBCA	PPIAL4A	DPT
TBKBP1	PPIB	DPY19L2P2
TBL1XR1	PPL	DPY19L2P4
TBX2	PPM1H	DPY19L4
TBX6	PPP1R12A	DPYD
TBXA2R	PPP1R12B	DPYS
TCAP	PPP1R2	DPYSL2
TCF7L1	PPP1R21	DPYSL5
TCFL5	PPP1R3B	DR1
TCIRG1	PPP1R3C	DRAM1
TDRD5	PPP2R4	DRAP1
TDRD9	PPP2R5A	DRD2
TEAD2	PPP2R5C	DRD3
TECPR1	PRDM1	DRD4
TES	PRDM2	DRD5
TEX19	PRDX6	DRG1
TFAP2C	PRELP	DRGX
TFE3	PREP	DSC2
TFF1	PREX2	DSC3
TGFA	PRF1	DSCAM
THAP8	PRICKLE1	DSCAML1
THBS1	PRIMA1	DSCC1
THNSL1	PRKAA2	DSCR3
THYN1	PRKAB2	DSCR8
TIAF1	PRKAG2	DSE
TIAM2	PRKAR2A	DSEL
TIGD1	PRKAR2B	DSG1
TIGD6	PRKCA	DSG2
TIMM13	PRKCB	DSG4
TINAGL1	PRKCD	DSPP
TINF2	PRKCE	DTD1
TIPARP	PRKCZ	DTL
TIPARP-AS1	PRKD1	DTNB
TJP1	PRKD3	DTWD2
TJP3	PRL	DTX1
TLE1	PRLR	DUOX1
TLL2	PROC	DUSP1
TLN2	PROK2	DUSP10

TLR6	PROM1	DUSP18
TMBIM6	PROS1	DUSP19
TMED4	PROZ	DUSP27
TMEM105	PRPF6	DUSP5
TMEM11	PRPS2	DUSP7
TMEM120A	PRRG1	DUSP9
TMEM140	PRRX1	DUT
TMEM143	PRSS23	DVL3
TMEM144	PRUNE	DYM
TMEM147	PSD2	DYNAP
TMEM150A	PSEN2	DYNC1I1
TMEM178A	PSMA1	DYNC1I2
TMEM184A	PSMB10	DYNC2H1
TMEM186	PSMC4	DYNC2LI1
TMEM189	PSMD13	DYNLL1
TMEM189-UBE2V1	PSMD7	DYNLRB1
TMEM205	PTBP1	DYNLRB2
TMEM215	PTBP2	DYRK1B
TMEM246	PTDSS2	DYRK2
TMEM37	PTGDS	DYSF
TMEM61	PTGES	DZANK1
TMPRSS13	PTGFR	DZIP1
TMPRSS4	PTGFRN	DZIP1L
TMSB4X	PTK2B	E2F1
TNC	PTK7	E2F3
TNFAIP1	PTMA	E2F7
TNFRSF12A	PTOV1	E2F8
TNFRSF18	PTP4A2	EARS2
TNFSF15	PTPN1	EBF1
TNNC1	PTPN14	EBF2
TNNI2	PTPN22	ECE1
TNRC18	PTPRD	ECE2
TNS2	PTPRF	ECEL1
TNS3	PTPRG	ECI2
TOMM40	PTPRM	ECT2
TOR2A	PTPRS	EDA
TP53I11	PTPRU	EDARADD
TP53I3	PVRL3	EDC3
TP53INP2	PWWP2B	EDDM3B
TPBG	PXDN	EDEM1
TPCN1	PXK	EDEM2
TPD52L1	PXYLP1	EDF1
TPD52L2	QPCT	EDN1
TPM1	QPRT	EDN3
TPM3	RAB26	EDNRA
TPPP	RAB2A	EEF1A2
TPRA1	RAB30	EEF1E1
TPRG1	RAB31	EEF2K
TRAF7	RAB43	EEF2KMT
TRAK2	RAB44	EEFSEC
TRAPPC2L	RAB6B	EEPD1
TRAPPC3	RAB9A	EFCAB11
TRIAP1	RABEP1	EFCAB2
TRIM14	RABGEF1	EFCAB6

TRIM2	RABGGTB	EFCAB7
TRIM38	RAD51B	EFCC1
TRIM47	RAD51D	EFHB
TRIP10	RADIL	EFHC1
TRMT12	RAI1	EFNA1
TRMT1L	RAI2	EFNA5
TRMT2A	RALGPS2	EFNB1
TRMT6	RALY	EFNB2
TRPC4AP	RAMP2	EFR3A
TRPM7	RAP1A	EFR3B
TSC2	RAP1GAP	EFTUD1
TSNAXIP1	RAP1GAP2	EGF
TSPAN14	RAP2B	EGFLAM
TSR3	RAPGEF4	EGFR
TSSC4	RAPGEFL1	EGLN1
TST	RARA	EGLN3
TTC3	RARB	EHBP1
TTC33	RASA3	EHBP1L1
TTC39B	RASEF	EHD4
TTC39C	RASGEF1B	EHF
TTC4	RASGRF1	EID2B
TUBA1A	RASSF3	EIF1
TUBA8	RASSF8	EIF1AX
TUBB2B	RB1	EIF1AY
TULP2	RB1CC1	EIF2AK1
TXNIP	RBBP8	EIF2AK3
TXNRD1	RBM25	EIF2B5
TYMP	RBM4	EIF3D
UBE2D1	RBMS1	EIF3H
UBE2F	RBMS3	EIF4E
UBE2F-SCLY	RBP2	EIF4E1B
UBE2I	RBPJ	ELAC1
UBE2O	RBPMS	ELAVL3
UBE2R2	RCAN1	ELAVL4
UBE3D	RCAN2	ELF2
UBP1	RCHY1	ELF3
UBQLN4	RCN1	ELF5
UCA1	RDH5	ELK1
UCKL1	RECK	ELL2
UCKL1-AS1	REEP5	ELMO1
UGT2B15	RERE	ELMO2
UGT2B17	RERG	ELMOD2
ULK4	RET	ELN
UNC13D	RFC1	ELOVL3
UNC93A	RFC5	ELOVL4
UNK	RFTN1	ELOVL7
UPF1	RFX3	ELP3
UPK3B	RGL1	ELP4
UQCRBP1	RGMA	ELP5
URB1	RGPD5	ELSPBP1
URB1-AS1	RGPD6	EMB
USP2	RGS3	EMC2
USP31	RGS6	EMC7
USP36	RGS7	EMCN

USP40	RHBDD1	EMID1
USP42	RHBDL2	EML1
USP5	RHOB	EML4
USPL1	RHOBTB1	EML5
UTP3	RHOH	EML6
VCAN	RMND5B	EN2
VEPH1	RNASE11	ENG
VIPR1	RND1	ENHO
VNN2	RNF144A	ENO2
VPS11	RNF144B	ENOX1
VPS13D	RNF182	ENOX2
VPS33A	RNF207	ENPEP
VPS35	RNF5	ENPP2
VPS37C	RNF7	ENPP3
VPS37D	RNLS	ENPP4
VPS9D1	RNMT	ENPP7
VWA2	RNPEP	ENTHD1
VWA3B	ROBO2	ENTPD2
WARS	ROCK2	ENTPD3
WDR25	ROR1	ENTPD6
WDR26	ROR2	ENY2
WDR59	RORA	EPAS1
WDR62	RORC	EPB41
WDR78	RPE	EPB41L2
WDR87	RPH3AL	EPB41L4B
WDR89	RPL15	EPB41L5
WFDC2	RPL3	EPC1
WIPI1	RPRM	EPG5
WISP2	RPS16	EPHA1
WNK1	RPS23	EPHA10
WNT10A	RPS27L	EPHA2
WTIP	RPS3	EPHA3
WWC2	RPS6KA3	EPHA4
WWC2-AS2	RRAS2	EPHA5
WWP1	RRBP1	EPHA7
WWTR1-AS1	RS1	EPHB1
XPO7	RSAD2	EPHB2
XYLB	RSPO1	EPHB4
YDJC	RSPO3	EPHX1
YIPF1	RTN4	EPHX4
YPEL2	RUNDC3A	EPM2A
YRDC	RUSC2	EPN1
YY1AP1	RWDD3	EPN2
ZBBX	RXFP1	EPN3
ZBED1	RXFP2	EPRS
ZBTB37	RXRB	EPS15
ZBTB40	RYR2	EPS15L1
ZBTB41	RYR3	EPS8
ZBTB47	S1PR1	EPS8L1
ZBTB48	S1PR3	EPS8L3
ZBTB5	S1PR5	EPSTI1
ZBTB7A	SAA1	EPT1
ZBTB7B	SAA2	EQTN
ZC2HC1C	SAA3P	ERAL1

ZC3H15	SAA4	ERAP1
ZCCHC7	SALL1	ERBB2IP
ZFAND4	SALL4	ERBB4
ZFAT	SAMD4A	ERC2
ZFP28	SAR1B	ERC2-IT1
ZFYVE28	SASH1	ERCC4
ZG16B	SAXO1	ERCC5
ZGPAT	SC5D	ERCC6
ZIC2	SCAF11	ERCC8
ZKSCAN2	SCAP	EREG
ZMAT3	SCARA5	ERGIC1
ZMYND11	SCARB1	ERGIC3
ZNF133	SCARB2	ERI1
ZNF140	SCD5	ERI3
ZNF146	SCGB1A1	ERICH1
ZNF185	SCGB3A1	ERICH5
ZNF22	SCML4	ERICH6B
ZNF251	SCNN1B	ERMARD
ZNF276	SCOC	ERMP1
ZNF280D	SCUBE1	ERN1
ZNF281	SCYL1	ERO1L
ZNF3	SCYL3	ERO1LB
ZNF302	SDC3	ERP44
ZNF330	SDC4	ERRFI1
ZNF335	SDK1	ESF1
ZNF341	SDS	ESPN
ZNF346	SDSL	ESR1
ZNF366	SEC13	ESRP1
ZNF37A	SEC14L2	ESRRB
ZNF436	SEC14L4	ESRRG
ZNF436-AS1	SEC24B	ETF1
ZNF44	SEC24D	ETFA
ZNF441	SEL1L	ETNK1
ZNF443	SEL1L3	ETNK2
ZNF474	SELK	ETS1
ZNF485	SEMA3C	ETV3
ZNF491	SEMA3E	ETV6
ZNF513	SEMA3F	EVA1C
ZNF532	SEMA4A	EVI5
ZNF552	SEMA4B	EVX2
ZNF565	SEMA4G	EWSAT1
ZNF567	SEMA5A	EXO1
ZNF571	SEMA6A	EXOC1
ZNF579	SENP6	EXOC2
ZNF581	SEPHS2	EXOC3
ZNF583	SERBP1	EXOC3L2
ZNF592	SERF2	EXOC3L4
ZNF598	SERP2	EXOC4
ZNF599	SERPINA1	EXOC6
ZNF605	SERPINA11	EXOG
ZNF621	SERPINA3	EXOSC6
ZNF629	SERPINA6	EXT1
ZNF641	SERPINB1	EXT2
ZNF644	SERPINB9	EYA1

ZNF669	SERPINC1	EYA2
ZNF749	SERPIND1	EYA3
ZNF786	SERPINE1	EYS
ZNF787	SERPINE2	F11
ZNF799	SERTAD2	F11R
ZNF812	SETBP1	F13A1
ZNF823	SF3B1	F9
ZNRF1	SFRP1	FABP12
ZSCAN22	SFTPB	FABP2
ZSCAN31	SFXN1	FABP5
ZSWIM6	SFXN2	FABP7
	SGIP1	FABP9
	SGK1	FADS3
	SGK3	FAF1
	SGMS1	FAIM
	SH2D4A	FAM105A
	SH3BGRL	FAM107B
	SH3BP4	FAM110A
	SH3BP5	FAM110B
	SH3D21	FAM110C
	SH3GL1	FAM114A1
	SH3GL2	FAM117B
	SH3GLB1	FAM118A
	SH3PXD2A	FAM118B
	SHISA3	FAM120A
	SHPRH	FAM120B
	SIAH1	FAM126A
	SIGMAR1	FAM129A
	SIK1	FAM131C
	SIPA1L1	FAM134B
	SIPA1L3	FAM134C
	SIRT3	FAM135A
	SIRT7	FAM135B
	SKAP2	FAM13A
	SLC12A4	FAM13A-AS1
	SLC12A8	FAM13B
	SLC13A4	FAM13C
	SLC16A10	FAM149A
	SLC16A2	FAM150B
	SLC16A6	FAM151B
	SLC16A9	FAM155A
	SLC17A1	FAM160A2
	SLC19A2	FAM160B1
	SLC20A2	FAM163A
	SLC22A1	FAM167B
	SLC22A23	FAM169A
	SLC22A4	FAM170A
	SLC22A6	FAM171A1
	SLC24A3	FAM171B
	SLC25A12	FAM172A
	SLC25A13	FAM174A
	SLC25A15	FAM175B
	SLC25A19	FAM178A
	SLC25A26	FAM181A

SLC25A30	FAM189A1
SLC25A33	FAM189A2
SLC25A36	FAM193A
SLC25A42	FAM196B
SLC26A7	FAM19A1
SLC26A8	FAM19A2
SLC27A2	FAM19A5
SLC27A6	FAM201A
SLC28A3	FAM205A
SLC29A1	FAM208A
SLC2A1	FAM208B
SLC30A5	FAM20C
SLC31A1	FAM210B
SLC33A1	FAM213A
SLC35A5	FAM214A
SLC35C1	FAM216A
SLC35D1	FAM218A
SLC35F1	FAM219B
SLC35F3	FAM26D
SLC35G1	FAM26F
SLC38A1	FAM27L
SLC38A3	FAM3B
SLC39A7	FAM3C
SLC39A8	FAM43A
SLC40A1	FAM46C
SLC44A1	FAM47C
SLC44A3	FAM47E
SLC45A1	FAM49A
SLC6A2	FAM53A
SLC7A1	FAM53B
SLC7A10	FAM53C
SLC7A11	FAM60A
SLC7A14	FAM63A
SLC7A8	FAM64A
SLC8A1	FAM65A
SLC9A8	FAM65B
SLCO2A1	FAM66A
SLCO2B1	FAM69C
SLCO5A1	FAM71B
SLIT2	FAM71C
SLITRK4	FAM71F1
SLPI	FAM81A
SLX4IP	FAM83B
SMAD1	FAM84B
SMAD5	FAM8A1
SMAD7	FAM91A1
SMARCA2	FAM92A1
SMARCA4	FAM96B
SMARCD3	FAM9B
SMARCE1	FAM9C
SMCO4	FAN1
SMG6	FANCC
SMOC1	FANCL
SMOC2	FAR2

SMOX	FARP1
SMPD3	FARS2
SMTN	FAS
SMYD3	FASTK
SNAI3	FASTKD2
SNAPC3	FAT1
SNCAIP	FAT2
SNED1	FATE1
SNN	FAXDC2
SNX24	FBL
SNX29	FBN1
SNX3	FBN2
SOCS7	FBN3
SOD1	FBP1
SORBS3	FBXL16
SORCS2	FBXL17
SOX11	FBXL18
SOX3	FBXL4
SOX5	FBXL5
SOX7	FBXL7
SPHKAP	FBXO10
SPINK14	FBXO15
SPINK7	FBXO16
SPIRE2	FBXO21
SPN	FBXO25
SPO11	FBXO27
SPOCK1	FBXO34
SPON1	FBXO38
SPOP	FBXO44
SPP2	FBXO46
SPPL2A	FBXO6
SPRED1	FBXO9
SPRED2	FBXW11
SPRY2	FBXW2
SPRY4	FBXW8
SPSB1	FCF1
SPTLC1	FCHO2
SPTLC2	FCHSD2
SPTY2D1	FCN3
SREBF1	FCRL5
SRGAP1	FCRLA
SRGAP2	FEM1C
SRGN	FEN1
SRP9	FER
SRSF1	FER1L4
SRSF2	FERD3L
SS18	FERMT2
SSBP1	FERMT3
SSFA2	FEZF2
SSH1	FFAR2
SSR3	FFAR4
ST3GAL1	FGA
ST3GAL3	FGD1
ST3GAL5	FGD2

ST6GALNAC6	FGD4
STAC2	FGF1
STAG1	FGF12
STAMBPL1	FGF14
STARD13	FGF17
STAT2	FGF18
STAT3	FGF2
STAT5B	FGF6
STC1	FGF7
STC2	FGF9
STEAP3	FGFR1
STK24	FGFR2
STK3	FGFR3
STK35	FGGY
STK39	FGL2
STMND1	FHAD1
STUB1	FHDC1
STXBP6	FHIT
SUCLG2	FHOD3
SULF2	FIBCD1
SULT1A1	FIBP
SVIL	FICD
SYBU	FIG4
SYNDIG1L	FIGN
SYNE1	FILIP1L
SYNJ2	FIP1L1
SYNPO	FJX1
SYT1	FKBP11
SYT12	FKBP1A
SYT6	FKBP5
SYTL2	FKBP9
SYTL4	FKTN
SYTL5	FLCN
TAB2	FLG2
TACC1	FLI1
TACC2	FLII
TACR1	FLJ41733
TAGAP	FLJ44796
TAMM41	FLJ45079
TANC1	FLJ46838
TARS	FLNB
TAS2R1	FLNC
TAT	FLOT2
TBC1D22A	FLT3LG
TBC1D4	FMN1
TBCE	FMN2
TBL1X	FMNL1
TBX3	FN1
TCF12	FNBP1L
TCF21	FNBP4
TCF23	FNDC3B
TCF4	FNIP2
TCF7L2	FOXB1
TCOF1	FOXB2

TDRD1	FOXD1
TEAD1	FOXE1
TEAD2	FOXF1
TEC	FOXF2
TECTA	FOXG1
TEF	FOXJ2
TEFM	FOXJ3
TENM2	FOXK1
TENM3	FOXK2
TENM4	FOXL1
TERT	FOXL2
TET2	FOXN1
TF	FOXN2
TFAM	FOXN3
TFAP2C	FOXN4
TFF1	FOXO3
TFF3	FOXP1
TFPI2	FOXP2
TG	FOXP3
TGFA	FOXP4
TGFB2	FOXQ1
TGFBI	FRAS1
TGFBR3	FRAT1
TGFBRAP1	FREM3
TGM6	FRK
THADA	FRMD3
THAP1	FRMD4A
THBD	FRMD4B
THBS2	FRMD5
THBS4	FRMD6
THEMIS	FRMPD1
THOC2	FRMPD2
THRB	FRMPD4
THSD4	FRRS1
TIA1	FRS2
TIAM2	FRY
TIMP3	FSCB
TJP1	FSHR
TLE1	FSIP1
TLE3	FSTL1
TLE4	FSTL4
TLL1	FSTL5
TLN2	FTH1
TM4SF1	FTH1P3
TM4SF4	FTMT
TM4SF5	FTSJ1
TM6SF2	FURIN
TM7SF2	FUT10
TM9SF2	FUT11
TMCC3	FUT8
TMCO4	FUT9
TMED10	FUZ
TMEM104	FXYD2
TMEM106B	FXYD3

TMEM121	FYCO1
TMEM133	FYN
TMEM144	FZD1
TMEM14C	FZD10
TMEM174	FZD3
TMEM185A	FZD4
TMEM229A	FZD5
TMEM247	FZD6
TMEM255B	FZD8
TMEM260	G2E3
TMEM45B	G3BP2
TMEM50B	G6PC2
TMEM64	GAB1
TMEM86A	GAB2
TMEM87A	GABBR2
TMOD1	GABRA1
TMPRSS11E	GABRA3
TMPRSS2	GABRA6
TMTC2	GABRB1
TNFAIP2	GABRB2
TNFAIP8L3	GABRB3
TNFRSF11B	GABRD
TNFRSF12A	GABRG1
TNFSF10	GABRG3
TNFSF15	GAD2
TNIP3	GADD45B
TNK2	GADD45G
TNKS2	GAL3ST3
TNPO2	GALC
TNR	GALE
TNRC18	GALM
TNS1	GALNT13
TNS3	GALNT14
TOB1	GALNT15
TOM1L2	GALNT18
TOMM40L	GALNT2
TOP1	GALNT3
TOP2B	GALNT6
TP53	GALNT9
TP53I11	GALNTL5
TP63	GALNTL6
TPD52L1	GALR1
TPM3	GAN
TPRG1	GANC
TPRN	GAP43
TPST1	GAPT
TRABD2B	GAREM
TRAF3IP2	GAS2
TRAM2	GAS7
TREH	GATA3
TRERF1	GATA4
TRIM11	GATA6
TRIM32	GATB
TRIM52	GATC

TRIM55	GBA
TRIM72	GBA2
TRIP10	GBE1
TRIP4	GBF1
TRIQK	GBGT1
TRPC7	GBP6
TRPS1	GBX2
TSC22D3	GC
TSGA13	GCC1
TSHZ2	GCFC2
TSPAN3	GCG
TSPYL1	GCLC
TTC14	GDA
TTC27	GDF9
TTF2	GDI2
TTLL2	GFM1
TTLL5	GFOD1
TTPA	GFRA1
TTR	GFRA2
TUBA1A	GGA2
TUBA1C	GGCT
TUBB8	GGN
TUFT1	GH1
TUSC1	GH2
TWIST2	GHR
TWISTNB	GHRHR
TWSG1	GIMAP2
TXLNB	GIP
TYMP	GIPC2
TYW1	GJB4
UBB	GJC1
UBE2R2	GK2
UBE2V1	GKN2
UBE4B	GLB1
UBL3	GLDC
UBR5	GLDN
UBTD2	GLI1
UBXN2A	GLI2
UGP2	GLI3
UGT1A10	GLIPR1
ULK4	GLIPR1L2
UMPS	GLIPR2
UNC13D	GLIS1
UOX	GLIS2
UROC1	GLIS3-AS1
USP1	GLRA2
USP10	GLRA3
USP24	GLRX
USP36	GLRX2
USP4	GLRX3
USP7	GLT1D1
UST	GLT8D2
UTRN	GLTP
UTS2	GLUD1

UVRAG	GLUL
VAMP5	GLYATL2
VASN	GLYCTK
VCL	GM2A
VCPIP1	GMDS
VEGFA	GMEB1
VEPH1	GMFB
VKORC1	GMNN
VLDLR	GMPPA
VPREB3	GMPR
VPS13B	GNA11
VPS37B	GNA12
VPS37C	GNA13
VTCN1	GNAI1
VWA2	GNAI2
VWF	GNAI3
WBP1L	GNAL
WBP5	GNAO1
WBSCR17	GNAS
WDR26	GNAT3
WDR81	GNB1
WDR83	GNB1L
WEE1	GNB4
WIPI1	GNE
WISP2	GNG7
WNT10B	GNL1
WNT9B	GNLY
WSCD2	GNPDA2
WT1	GNPNAT1
WWP1	GOLGA2P5
WWP2	GOLGA4
XBP1	GOLGA6D
XDH	GOLGA8A
XPNPEP1	GOLPH3
XPR1	GOLT1B
XRCC5	GOPC
YBX3	GORASP2
YPEL2	GOSR2
YTHDC1	GP5
ZAK	GP9
ZBP1	GPAM
ZBTB20	GPATCH2
ZBTB37	GPC5
ZBTB40	GPC6
ZBTB7C	GPD1L
ZC2HC1C	GPD2
ZC3H12C	GPHN
ZC3H4	GPLD1
ZC3H7B	GPM6A
ZC3HC1	GPNMB
ZCCHC10	GPR119
ZCCHC6	GPR137B
ZCCHC8	GPR142
ZDHHC1	GPR149

ZDHHC16	GPR15
ZEB2	GPR152
ZFAND3	GPR155
ZFHX3	GPR158
ZFP36L1	GPR17
ZFPM1	GPR25
ZHX3	GPR37
ZKSCAN2	GPR4
ZMAT4	GPR65
ZMYND8	GPR83
ZNF106	GPR87
ZNF146	GPRASP2
ZNF217	GPRC5D
ZNF281	GPRC6A
ZNF341	GPRIN1
ZNF358	GPRIN3
ZNF365	GPSM2
ZNF366	GPX2
ZNF385A	GPX7
ZNF423	GRAMD2
ZNF496	GRAMD3
ZNF503	GRAMD4
ZNF507	GRB10
ZNF525	GREB1
ZNF529	GREB1L
ZNF536	GRHL1
ZNF552	GRHL2
ZNF566	GRHPR
ZNF587	GRIA1
ZNF600	GRIA4
ZNF608	GRID1
ZNF611	GRID2
ZNF644	GRIK1
ZNF704	GRIK2
ZNF804A	GRIK4
ZNF827	GRIK5
ZNHIT6	GRIN2A
ZNRF1	GRIN3A
ZNRF2	GRIN3B
ZP1	GRK5
ZSWIM2	GRM1
	GRM3
	GRM4
	GRM7
	GRM8
	GRXCR1
	GSC
	GSC2
	GSDMC
	GSE1
	GSG1
	GSG1L
	GSG2
	GSK3B

GSPT1
GSR
GSTA3
GSTA4
GSTM2
GSTO2
GSTTP2
GSX1
GSX2
GTDC1
GTF2A1
GTF2E2
GTF2H3
GTF2I
GTF3C1
GTSE1-AS1
GTSF1
GUCA1A
GUCY1A3
GUCY2C
GUCY2D
GULP1
GUSB
GXYLT1
GYG1
GYPB
GYS2
H2AFJ
H2AFY
H2AFY2
H2AFZ
H6PD
HAAO
HABP2
HABP4
HACD1
HACD2
HACD4
HADH
HAMP
HAND2
HAO1
HAO2
HAPLN2
HAPLN3
HAS1
HAT1
HAUS2
HAUS8
HBS1L
HCAR2
HCLS1
HCN1
HCN4

HCP5
HCRTR1
HDAC4
HDAC5
HDAC9
HDGFL1
HDHD1
HEATR3
HEBP2
HELZ
HEPACAM2
HERC2
HERC2P2
HERC3
HERC4
HERPUD1
HERPUD2
HES2
HEXA
HEY2
HEYL
HFM1
HGD
HGF
HHIPL1
HHIPL2
HHLA2
HIBADH
HIBCH
HIF1A
HIF3A
HINFP
HIP1
HIP1R
HIPK2
HIST1H2AA
HIST1H2BD
HIST1H2BJ
HIST1H2BL
HIST1H3B
HIST1H3G
HIST1H4B
HIST1H4C
HIST1H4H
HIST1H4I
HIST2H2BF
HIST2H4A
HIVEP3
HK2
HK3
HLA-DMB
HLA-F
HLA-G
HLCS

HLF
HLX
HM13
HMCN1
HMG20B
HMGA2
HMGCLL1
HMGN1
HMGN2P46
HMGN4
HMGXB4
HMOX1
HMX2
HN1
HNF4G
HNMT
HNRNPAB
HNRNPH1
HNRNPK
HNRNPLL
HNRNPR
HNRNPU
HNRNPU-AS1
HOMER1
HOMER2
HOMER3
HOOK3
HOPX
HORMAD2
HOXA1
HOXA10
HOXA11
HOXA13
HOXA4
HOXA5
HOXA7
HOXB3
HOXC13
HOXC9
HOXD1
HOXD11
HOXD13
HOXD8
HPCA
HPCAL1
HPCAL4
HPD
HPGD
HPGDS
HPSE2
HPYR1
HRASLS
HRASLS2
HRH2

HRH3
HRH4
HRNR
HRSP12
HS1BP3
HS2ST1
HS3ST1
HS3ST3B1
HS3ST4
HS3ST5
HS6ST2
HS6ST3
HSCB
HSD11B1
HSD17B13
HSD17B3
HSD17B6
HSD3B1
HSD3B2
HSF1
HSF2
HSP90AB2P
HSPA12B
HSPA14
HSPA2
HSPA4
HSPA7
HSPA9
HSPB1
HSPB3
HSPBAP1
HSPD1
HTR1A
HTR1D
HTR1E
HTR2C
HTR3B
HTR4
HTR5A
HTR6
HTR7
HTRA1
HTRA4
HULC
HUWE1
HYDIN
IARS
IBTK
ICAM4
ICOSLG
ID2
ID2B
ID4
IDH3A

IDI2-AS1
IDO1
IFFO1
IFI27
IFIH1
IFIT1
IFNA2
IFNA5
IFNA8
IFNAR1
IFNB1
IFNG
IFNK
IFNL3
IFNLR1
IFNW1
IFT27
IFT43
IFT57
IFT80
IFT81
IGBP1
IGDCC4
IGF1
IGF1R
IGF2
IGF2BP3
IGF2R
IGFBP1
IGFBP2
IGFBP3
IGFBP5
IGFBP7
IGFL1
IGLL3P
IGSF11
IGSF8
IGSF9
IGSF9B
IKBKB
IKBKE
IKZF2
IL10RB
IL12B
IL12RB1
IL13RA1
IL15RA
IL17RA
IL17RE
IL1R1
IL1R2
IL1RAP
IL1RAPL1
IL1RAPL2

IL1RL2
IL20RA
IL21
IL27
IL2RA
IL31RA
IL37
IL6R
IL7
ILDR1
ILKAP
IMMP2L
IMPG1
IMPG2
INA
INADL
INAFM1
INCENP
INF2
ING1
INHBB
INHBC
INO80
INO80C
INPP1
INPP5A
INPP5B
INPPL1
INSC
INSIG2
INSL6
INSR
INTS4P2
INTS7
INVS
IP6K2
IP6K3
IPO4
IPO7
IPW
IQCE
IQCG
IQCH
IQGAP1
IQGAP2
IQGAP3
IQSEC1
IQUB
IRAK1BP1
IRF1
IRF2
IRF2BP2
IRF4
IRS2

IRX1
IRX2
IRX3
IRX4
IRX5
IRX6
ISCA1
ISG15
ISG20
ISL1
ISL2
ISLR
ISYNA1
ITCH
ITFG1
ITGA2
ITGA6
ITGAE
ITGAV
ITGB1
ITGB3
ITGB3BP
ITGB4
ITGB8
ITGBL1
ITIH2
ITIH5
ITIH6
ITLN1
ITM2C
ITPK1
ITPKB
ITPKC
ITPR2
ITPRIPL1
ITSN2
IWS1
JADE1
JADE2
JAG1
JAKMIP2
JARID2
JAZF1
JMJD7-PLA2G4B
JMY
KALRN
KANK1
KANSL1
KAT2B
KAT6B
KATNAL2
KBTBD11
KBTBD12
KBTBD8

KC6
KCMF1
KCNA2
KCNA4
KCNAB1
KCNB2
KCNC1
KCNC2
KCND3
KCNE4
KCNG1
KCNG3
KCNH2
KCNH5
KCNH6
KCNH8
KCNIP1
KCNIP4
KCNJ2
KCNJ3
KCNJ5
KCNJ6
KCNK1
KCNK12
KCNK18
KCNK5
KCNK9
KCNMB2
KCNN2
KCNQ1
KCNS2
KCNT1
KCNT2
KCNU1
KCNV2
KCTD1
KCTD12
KCTD15
KCTD21
KCTD3
KCTD4
KCTD9
KDELR1
KDELR2
KDM1A
KDM2A
KDM3B
KDM4A
KDM4B
KDM4C
KDM5A
KDM5B
KDM6A
KDR

KERA
KHDC3L
KHDRBS2
KIAA0020
KIAA0040
KIAA0101
KIAA0232
KIAA0319L
KIAA0355
KIAA0513
KIAA0556
KIAA0586
KIAA0753
KIAA1024
KIAA1147
KIAA1161
KIAA1217
KIAA1324
KIAA1407
KIAA1429
KIAA1456
KIAA1468
KIAA1522
KIAA1524
KIAA1551
KIAA1614
KIAA1683
KIAA1841
KIDINS220
KIF13A
KIF13B
KIF1BP
KIF20A
KIF21B
KIF23
KIF24
KIF25
KIF2A
KIF2B
KIF5B
KIF6
KIF7
KIF9
KIFAP3
KIFC1
KIR2DL1
KIR2DS4
KIRREL3
KITLG
KIZ
KLC1
KLF12
KLF13
KLF15

KLF2
KLF3
KLF6
KLF7
KLF9
KLHDC7A
KLHDC8A
KLHDC8B
KLHL10
KLHL11
KLHL13
KLHL15
KLHL2
KLHL20
KLHL3
KLHL31
KLHL34
KLHL35
KLHL6
KLHL8
KLHL9
KLK15
KLK2
KLK3
KLK8
KLK9
KLKB1
KLKP1
KLRF1
KLRG2
KMT2A
KNDC1
KPNA6
KRBOX4
KREMEN1
KRT10
KRT18
KRT33B
KRT37
KRT4
KRT5
KRT6B
KRT75
KRT80
KRT81
KRT86
KRTAP1-5
KRTAP10-10
KRTAP10-5
KRTAP11-1
KRTAP15-1
KRTAP19-8
KRTAP20-2
KRTAP21-2

KRTAP22-2
KRTAP5-6
KSR1
KSR2
KTN1
KTN1-AS1
KU-MEL-3
KXD1
KY
KYNU
L2HGDH
L3HYPDH
LACE1
LACTB2
LAIR2
LAMA1
LAMA2
LAMA3
LAMA4
LAMA5
LAMB4
LAMC1
LAMC3
LANCL2
LAPTM4B
LARGE
LARS
LARS2
LAS1L
LASP1
LBP
LBX1
LCE1C
LCE1D
LCE2A
LCE2B
LCE3B
LCE3C
LCK
LCLAT1
LCMT1
LCN1
LCN2
LCP1
LCT
LCTL
LDB2
LDB3
LDHA
LDHAL6B
LDHB
LDLRAD2
LDLRAD3
LDLRAD4

LDLRAP1
LEF1
LEKR1
LELP1
LEMD1
LEMD2
LEMD3
LENEP
LEO1
LEP
LEPR
LEPROTL1
LETM1
LETM2
LGALS3
LGALS3BP
LGI1
LGR4
LGR6
LHCGR
LHFP
LHFPL2
LHFPL3
LHFPL4
LHPP
LHX5
LHX8
LHX9
LIFR
LIG1
LIG4
LILRB1
LIM2
LIMD1
LIN28A
LIN28B
LIN37
LIN7A
LINC00052
LINC00113
LINC00158
LINC00160
LINC00167
LINC00189
LINC00222
LINC00244
LINC00310
LINC00320
LINC00323
LINC00467
LINC00469
LINC00471
LINC00474
LINC00476

LINC00479
LINC00518
LINC00521
LINC00575
LINC00588
LINC00589
LINC01465
LINC01551
LINC01553
LINC01555
LINC01559
LINGO1
LINGO2
LIPC
LIPE
LIPH
LIX1
LIX1L
LLPH
LMBR1
LMNB1
LMNTD1
LMNTD2
LMO2
LMO3
LMO4
LMO7
LMOD3
LMX1A
LMX1B
LNPEP
LOC100128164
LOC100128239
LOC100131496
LOC100133920
LOC100310782
LOC145474
LOC145837
LOC256880
LOC284788
LOC284798
LOC285627
LOC285692
LOC285696
LOC339568
LOC340357
LOC400499
LOC400794
LOC400940
LOC401463
LOC407835
LOC440173
LOC441204
LOC441242

LOC441956
LOC644669
LOC645166
LOC729603
LOC81691
LOC93432
LOH12CR1
LONP1
LONP2
LONRF1
LONRF2
LONRF3
LOXL1
LOXL2
LOXL4
LPA
LPAR3
LPGAT1
LPIN2
LPIN3
LPO
LPP
LPPR1
LPPR5
LRBA
LRFN3
LRGUK
LRIF1
LRIG1
LRIG3
LRP12
LRP1B
LRP2
LRP5
LRP5L
LRP8
LRR1
LRRC1
LRRC16A
LRRC16B
LRRC17
LRRC18
LRRC28
LRRC3
LRRC36
LRRC37A
LRRC37B
LRRC3B
LRRC41
LRRC4C
LRRC7
LRRC75A
LRRC8D
LRRFIP1

LRRFIP2
LRRIQ3
LRRIQ4
LRRN3
LRRN4
LRRTM1
LRRTM4
LRTM1
LSM12
LSM3
LSM4
LSM5
LSM7
LSR
LTK
LTN1
LUC7L
LUC7L3
LURAP1L
LUZP2
LUZP4
LY86
LY9
LYAR
LYPLAL1
LYRM4
LYSMD2
LYZL4
LZIC
LZTR1
MAATS1
MAB21L2
MACC1
MACF1
MACROD1
MACROD2
MAD1L1
MAD2L1
MAEA
MAF
MAFA
MAFB
MAGEB18
MAGED4
MAGEE1
MAGEL2
MAGI1
MAGI2
MAGI3
MAGOHB
MAL2
MALAT1
MALL
MALSU1

MALT1
MAML2
MAML3
MAMLD1
MAN1A1
MAN1A2
MAN1C1
MAN2A1
MANBAL
MAOA
MAOB
MAP1A
MAP1B
MAP1LC3B2
MAP1LC3C
MAP2K1
MAP2K4
MAP2K5
MAP2K6
MAP2K7
MAP3K10
MAP3K11
MAP3K4
MAP3K5
MAP4
MAP4K4
MAP4K5
MAP6
MAP7
MAP7D1
MAPK11
MAPK15
MAPK4
MAPK6
MAPK8IP2
MAPKAP1
MAPRE1
MAPRE2
MARC1
MARCH10
MARCKS
MARCO
MARK1
MARK4
MARS2
MARVELD3
MASP1
MAST4
MATN2
MATN3
MATN4
MAX
MB21D2
MBD2

MBD3
MBD5
MBL1P
MBL2
MBNL3
MBOAT1
MBOAT2
MBP
MC5R
MCC
MCCC2
MCF2L
MCF2L2
MCFD2
MCL1
MCM10
MCM3AP
MCM4
MCM6
MCMDC2
MCOLN1
MCOLN2
MCPH1
MCTP1
MCTP2
MDFIC
MDGA1
MDM1
MDM2
MDM4
ME2
MEA1
MECOM
MECP2
MED12L
MED13L
MED14
MED15
MED17
MED18
MED23
MED25
MED27
MED8
MED9
MEDAG
MEF2C
MEF2D
MEG3
MEGF10
MEGF11
MEGF6
MEGF8
MEI1

MEIG1
MEIS1
MEIS2
MELK
MEMO1
MEOX1
MEP1A
MEPE
MERTK
MESDC1
MET
METAP1D
METRNL
METTL15
METTL17
METTL21A
METTL22
METTL6
METTL8
METTL9
MEX3B
MFAP3
MFHAS1
MFNG
MFSD10
MFSD2B
MGAT3
MGAT4A
MGAT4C
MGAT5B
MGC16142
MGLL
MGME1
MGMT
MGRN1
MGST1
MICAL1
MICAL2
MICU2
MID1
MID2
MIEN1
MIER1
MIER3
MIIP
MINPP1
MIPEP
MIPOL1
MIR1-2
MIR101-2
MIR103A2
MIR1203
MIR1204
MIR1205

MIR1243
MIR1246
MIR1297
MIR1324
MIR137
MIR1470
MIR147A
MIR148A
MIR1538
MIR181B1
MIR186
MIR190A
MIR199A2
MIR2052
MIR2110
MIR2278
MIR30B
MIR30D
MIR32
MIR320C1
MIR340
MIR367
MIR383
MIR423
MIR491
MIR492
MIR515-2
MIR520A
MIR548C
MIR548F1
MIR548F4
MIR548H2
MIR548H3
MIR548Q
MIR563
MIR572
MIR580
MIR613
MIR623
MIR632
MIR718
MIR885
MIR9-1
MIR99AHG
MIRLET7I
MITF
MKI67
MKL1
MKL2
MKLN1
MKNK1
MKRN1
MLC1
MLLT3

MLNR
MLXIPL
MMAA
MMAB
MMADHC
MMP16
MMP2
MMP20
MMP24
MMP28
MMP3
MMP7
MMS22L
MND1
MOB1B
MOB3B
MOB4
MOBP
MOCS3
MON2
MORC1
MORC2
MORC3
MORF4L1
MORN1
MORN2
MOS
MPC2
MPDZ
MPHOSPH10
MPHOSPH6
MPP1
MPP5
MPP6
MPP7
MPPED1
MPPED2
MPRIP
MPZL1
MRAP
MRAP2
MRAS
MRC2
MRE11A
MRGPRD
MRGPRG-AS1
MROH6
MRPL1
MRPL14
MRPL18
MRPL21
MRPL23
MRPL32
MRPL33

MRPL38
MRPL44
MRPL46
MRPL47
MRPL48
MRPS11
MRPS14
MRPS16
MRPS2
MRPS27
MRPS28
MRPS31
MRPS34
MRVI1
MS4A8
MSANTD2
MSH2
MSH3
MSH4
MSI2
MSL1
MSL3P1
MSN
MSRA
MSTN
MSX1
MSX2
MTAP
MTBP
MTCL1
MTDH
MTERF1
MTERF3
MTFP1
MTFR1
MTHFD1
MTHFD1L
MTHFD2L
MTIF3
MTL5
MTM1
MTMR11
MTMR12
MTMR14
MTMR2
MTMR3
MTMR4
MTMR9
MTPN
MTR
MTRR
MTUS1
MTUS2
MTX2

MUC12
MUSK
MUT
MVB12B
MVK
MXD3
MXRA5
MXRA7
MYB
MYBBP1A
MYBL1
MYBPC2
MYBPH
MYC
MYCBP2
MYCBPAP
MYCL
MYEF2
MYF5
MYH10
MYH15
MYH4
MYH9
MYL10
MYL12A
MYL9
MYLIP
MYLK
MYLK4
MYO10
MYO16
MYO1A
MYO1B
MYO1C
MYO1D
MYO1E
MYO3A
MYO3B
MYO5A
MYO6
MYO7A
MYO9A
MYO9B
MYOCD
MYOF
MYOM2
MYOM3
MYOT
MYPN
MYRFL
MYRIP
MYT1L
N4BP2
N6AMT2

NAA16
NAA25
NAA35
NAA40
NAALAD2
NAALADL2
NACAP1
NACC2
NADSYN1
NAGK
NAGPA
NANOG
NAPEPLD
NARF
NAT16
NAV1
NAV2
NAV3
NBAS
NBEA
NBPF1
NBPF10
NCALD
NCAM1
NCAM2
NCBP1
NCEH1
NCK2
NCKAP1
NCKAP1L
NCKIPSD
NCL
NCOA1
NCOA2
NCOA3
NCOA7
NCOR1P1
NCOR2
NDC1
NDC80
NDFIP1
NDP
NDRG1
NDRG3
NDST4
NDUFA12
NDUFA3
NDUFAF2
NDUFAF5
NDUFAF6
NDUFAF7
NDUFB2
NDUFC2
NDUFS1

NDUFS2
NEB
NEBL
NECAB1
NECAB3
NEDD4
NEDD4L
NEDD9
NEFH
NEFL
NEFM
NEGR1
NEIL2
NEK1
NEK10
NEK11
NEK2
NEK3
NEK6
NELL1
NELL2
NEO1
NET1
NETO1
NEU3
NEURL1
NEUROG3
NF1
NFAM1
NFASC
NFAT5
NFATC1
NFATC2
NFE2
NFE2L1
NFIA
NFIB
NFIC
NFIL3
NFKB2
NFKBIA
NFRKB
NFYB
NGDN
NGF
NGLY1
NHEJ1
NHLRC2
NHS
NID1
NID2
NIN
NIP7
NIPAL1

NIPAL2
NIPAL4
NIPBL
NIPSNAP3B
NIT2
NKAIN1
NKAIN2
NKIRAS1
NKX2-2
NKX2-3
NKX2-6
NKX2-8
NKX3-1
NKX6-1
NLGN4X
NLGN4Y
NLRC3
NLRC5
NLRP13
NLRP2
NMBR
NMD3
NME3
NME4
NME6
NME7
NMRK2
NMT1
NMT2
NNMT
NOBOX
NOD1
NOD2
NOL10
NOL11
NOL4
NOL4L
NOLC1
NOM1
NOP2
NOP58
NOS1AP
NOS2
NOSIP
NOTCH3
NOVA1
NOVA2
NOX3
NOX5
NOXO1
NOXRED1
NPAP1
NPAS2
NPAS3

NPAS4
NPAT
NPC1
NPEPL1
NPHP1
NPHP4
NPNT
NPPC
NPTX1
NPVF
NPY
NPY1R
NPY2R
NR0B1
NR1D1
NR1H4
NR1I2
NR2F1
NR2F2
NR3C1
NR3C2
NR4A1
NR5A2
NR6A1
NRADDP
NRCAM
NRF1
NRG1
NRG2
NRG3
NRIP1
NRP1
NRP2
NRSN1
NRTN
NRXN1
NRXN2
NRXN3
NSDHL
NSG1
NSMAF
NSUN3
NSUN5P2
NT5E
NTF3
NTHL1
NTM
NTN1
NTNG1
NTNG2
NTRK2
NTRK3
NUAK1
NUAK2

NUBP1
NUBPL
NUDCD3
NUDT10
NUDT12
NUDT7
NUDT9
NUFIP1
NUFIP2
NUGGC
NUP107
NUP155
NUP210
NUP35
NUP50
NUP54
NUP62
NUP93
NUPL1
NUPL2
NUTF2
NUTM2F
NWD1
NXF3
NXPE1
NXPH1
NXPH2
NXPH3
NYAP2
NYX
OAF
OAS1
OAS2
OBSCN
OBSL1
ODF1
ODF2L
ODF3L1
OGDH
OGFOD2
OGFR
OGG1
OGN
OLA1
OLFM1
OLFM3
OLFML1
OLFML3
OLIG3
OLR1
OMP
ONECUT1
ONECUT2
OPALIN

OPCML
OPHN1
OPLAH
OPN1SW
OPN4
OPN5
OPRD1
OPRK1
OR10A7
OR10G2
OR10H2
OR10J1
OR10K1
OR10K2
OR10S1
OR10T2
OR1E1
OR1J1
OR1J2
OR1K1
OR1L3
OR1L4
OR2A14
OR2A20P
OR2A25
OR2A5
OR2A7
OR2B11
OR2F1
OR2S2
OR2T11
OR4A15
OR4A47
OR4D1
OR4D9
OR4E2
OR4K2
OR4M1
OR4N5
OR4Q3
OR4X2
OR51D1
OR51E2
OR52I1
OR52K2
OR52M1
OR56A3
OR5B21
OR5H6
OR5K1
OR5M11
OR5V1
OR6K2
OR6W1P

OR7E37P
OR8B12
OR8B8
OR8H1
OR8S1
OR9Q1
OR9Q2
ORAI3
ORC1
ORC4
ORC5
ORMDL1
OSBPL1A
OSBPL3
OSBPL5
OSBPL8
OSBPL9
OSR2
OSTN
OTOF
OTOL1
OTOR
OTUB1
OTUD6B
OTUD7A
OTUD7B
OTULIN
OTX2-AS1
OVCA2
OVCH1
OXCT1
OXER1
OXSM
OXT
P2RY1
P2RY10
P2RY14
P2RY6
P4HA1
P4HA3
PABPC1
PABPC3
PABPC4
PACRG
PACRGL
PACS1
PACS2
PACSIN2
PADI2
PADI6
PAEP
PAFAH2
PAG1
PAH

PAICS
PAIP2
PAIP2B
PAK1
PAK7
PALD1
PALLD
PALM
PALM2-AKAP2
PAM
PAMR1
PANK1
PAPD4
PAPD5
PAPD7
PAPOLA
PAPPA
PAPPA2
PAPSS1
PAPSS2
PAQR5
PAQR8
PAQR9
PARD3
PARD3B
PARD6B
PARG
PARK2
PARM1
PARN
PARP11
PARP14
PARP15
PARP4
PARP6
PARP8
PARS2
PART1
PARVA
PASD1
PASK
PATL1
PAX1
PAX2
PAX5
PAX6
PAX7
PAX9
PAXBP1
PAXIP1
PBK
PBOV1
PBX1
PBXIP1

PC
PCBD2
PCBP1
PCBP3
PCDH1
PCDH10
PCDH11X
PCDH15
PCDH17
PCDH18
PCDH20
PCDH7
PCDH9
PCDHGA3
PCED1B
PCF11
PCGEM1
PCGF2
PCGF3
PCK1
PCMTD1
PCMTD2
PCNXL2
PCSK1
PCSK2
PCSK5
PCSK6
PCTP
PDC
PDCD1
PDCD6IP
PDCD7
PDCL3
PDE10A
PDE11A
PDE12
PDE2A
PDE4B
PDE4D
PDE4DIP
PDE6A
PDE6H
PDE7A
PDE8B
PDE9A
PDGFA
PDGFB
PDGFRA
PDHB
PDHX
PDIA3
PDIA5
PDIK1L
PDK1

PDK2
PDK4
PDLIM3
PDLIM5
PDPN
PDS5B
PDSS2
PDYN
PDZD2
PDZD4
PDZD8
PDZRN3
PEBP4
PECAM1
PEG3
PELI2
PELO
PENK
PER1
PER2
PER4
PEX1
PEX10
PEX2
PEX26
PEX3
PEX5L
PF4
PF4V1
PFDN1
PFKFB3
PFKFB4
PFKM
PGAP1
PGBD3
PGC
PGK1
PGK2
PGLYRP2
PGM1
PGM2
PGM2L1
PGPEP1
PGPEP1L
PGRMC1
PHACTR1
PHACTR2
PHACTR3
PHACTR4
PHC2
PHF12
PHF13
PHF20L1
PHF21A

PHF21B
PHIP
PHKB
PHLDA3
PHLDB1
PHLDB2
PHLPP1
PHOX2B
PHYH
PHYHIPL
PI15
PIAS1
PIAS3
PICALM
PICK1
PIEZO2
PIGB
PIGC
PIGG
PIGK
PIGL
PIGP
PIH1D2
PIK3AP1
PIK3C2A
PIK3C2B
PIK3C2G
PIK3CA
PIK3CD
PIK3CG
PIK3IP1
PIK3R1
PIK3R2
PIK3R3
PIK3R4
PIK3R5
PIM3
PINX1
PIP
PIP5K1C
PIP5KL1
PIR
PISD
PITPNB
PITPNC1
PITPNM2
PITPNM3
PITRM1
PITX1
PITX3
PIWIL1
PJA1
PJA2
PKD1L1

PKD1L2
PKD2
PKD2L2
PKDCC
PKHD1
PKIA
PKIB
PKN1
PKNOX1
PKNOX2
PKP2
PLA2G12B
PLA2G1B
PLA2G4C
PLA2G4F
PLA2G5
PLA2G6
PLAA
PLAG1
PLAT
PLB1
PLCB4
PLCE1
PLCG1
PLCG2
PLCXD2
PLCXD3
PLD1
PLD4
PLEC
PLEKHA1
PLEKHA5
PLEKHA7
PLEKHA8
PLEKHG3
PLEKHH1
PLEKHM3
PLEKHN1
PLET1
PLGRKT
PLIN1
PLIN2
PLOD2
PLS1
PLSCR4
PLTP
PLXDC2
PLXNA2
PLXNA4
PLXNC1
PMAIP1
PMCH
PMEPA1
PML

PMP22
PMPCA
PMS1
PMS2
PMVK
PNLIP
PNLIPRP1
PNN
PNRC1
POC1A
POC5
PODXL
POFUT1
POLA1
POLA2
POLB
POLD3
POLE2
POLE3
POLE4
POLG
POLQ
POLR2A
POLR2M
POLR3E
POLR3GL
POM121L12
POMC
POMGNT2
PON3
POP4
POP7
POTED
POTEE
POU1F1
POU2F1
POU2F2
POU3F1
POU3F2
POU3F4
POU4F3
POU5F1B
POU5F2
POU6F1
POU6F2
PPAP2A
PPAP2B
PPAPDC1A
PPAPDC1B
PPAPDC2
PPARA
PPARG
PPARGC1A
PPAT

PPBPP2
PPCDC
PPEF1
PPFIA1
PPFIBP1
PPFIBP2
PPIAL4F
PPID
PPIE
PPIL2
PPIL4
PPIP5K2
PPM1A
PPM1B
PPM1H
PPM1J
PPM1K
PPM1L
PPP1CB
PPP1CC
PPP1R12A
PPP1R14C
PPP1R16B
PPP1R2
PPP1R21
PPP1R2P3
PPP1R32
PPP1R36
PPP1R3B
PPP1R3G
PPP1R9A
PPP1R9B
PPP2R1A
PPP2R2B
PPP2R2C
PPP2R5C
PPP2R5E
PPP3CA
PPP4R1
PPP4R1L
PPP4R4
PPP6C
PPP6R1
PPP6R2
PPP6R3
PPRC1
PPT1
PPTC7
PQLC2
PQLC2L
PRAC1
PRAMEF20
PRB1
PRDM1

PRDM11
PRDM12
PRDM13
PRDM14
PRDM15
PRDM16
PRDM7
PRDM9
PRDX4
PRDX5
PRDX6
PRELID2
PREP
PREX1
PREX2
PRG2
PRG3
PRG4
PRH1
PRICKLE1
PRIM1
PRIMA1
PRKAA2
PRKACB
PRKACG
PRKAG1
PRKAG2
PRKAR1B
PRKAR2B
PRKCA
PRKCB
PRKCE
PRKCH
PRKCI
PRKCSH
PRKCZ
PRKD1
PRKD3
PRKDC
PRKG1
PRKG2
PRKRIP1
PRLH
PRLR
PRMT2
PRMT3
PRMT6
PRODH2
PROSC
PROSER2
PROX1
PRPF3
PRPF38B
PRPF4B

PRPF8
PRPS1
PRPS1L1
PRPS2
PRPSAP1
PRR14L
PRR15L
PRR20E
PRR35
PRR5
PRRX1
PRRX2
PRSS1
PRSS50
PRSS55
PRTFDC1
PRTG
PRUNE2
PSAT1
PSCA
PSD3
PSEN2
PSG8
PSKH2
PSMA1
PSMA4
PSMA5
PSMA6
PSMB2
PSMC6
PSMD1
PSMD12
PSMD14
PSMD6
PSMD7
PSMD9
PSMF1
PSMG1
PSMG4
PSPH
PSTPIP1
PTBP2
PTCD2
PTCD3
PTCH1
PTDSS2
PTER
PTGER2
PTGER3
PTGER4
PTGES
PTGFRN
PTGIS
PTGS1

PTH
PTHLH
PTK2
PTK7
PTN
PTOV1
PTP4A1
PTPDC1
PTPN1
PTPN11
PTPN12
PTPN13
PTPN14
PTPN20
PTPN21
PTPN22
PTPN3
PTPN4
PTPRC
PTPRD
PTPRE
PTPRF
PTPRG
PTPRJ
PTPRK
PTPRM
PTPRN2
PTPRR
PTPRT
PTS
PTTG2
PTX3
PUM1
PUM2
PUS1
PVRL1
PVT1
PWP1
PWWP2A
PXDC1
PXDN
PXK
PXN
PYDC2
PYGB
PYGO1
QDPR
QKI
QRICH1
QRICH2
R3HCC1L
R3HDM1
RAB11B
RAB11FIP3

RAB14
RAB17
RAB18
RAB1A
RAB1B
RAB21
RAB25
RAB27A
RAB27B
RAB30
RAB31
RAB33A
RAB35
RAB3B
RAB3C
RAB4A
RAB6A
RAB6B
RAB8A
RAB8B
RAB9BP1
RABEP1
RABGAP1L
RABGGTA
RABGGTB
RABIF
RABL3
RABL6
RAC1
RAC3
RAD18
RAD21
RAD51B
RAD51C
RAD51D
RAD54B
RAD9A
RAE1
RAF1
RAG2
RAI1
RAI14
RAI2
RALA
RALBP1
RALGAPA1
RALGAPA2
RALGAPB
RALGPS2
RALY
RALYL
RAMP1
RAN
RANBP1

RANBP10
RANBP17
RANBP3L
RANBP9
RANGAP1
RAP1A
RAP1B
RAP1GAP
RAP2A
RAP2B
RAPGEF1
RAPGEF4
RAPGEF5
RARB
RARRES1
RARRES2
RARRES3
RARS
RASA1
RASAL2
RASAL3
RASD1
RASGEF1B
RASGEF1C
RASGRF1
RASGRF2
RASGRP1
RASGRP3
RASL12
RASSF1
RASSF3
RASSF5
RASSF6
RASSF8
RASSF9
RB1CC1
RBBP8
RBBP8NL
RBFOX1
RBL1
RBM12B
RBM12B-AS1
RBM19
RBM20
RBM25
RBM28
RBM33
RBM4
RBM45
RBM47
RBM6
RBMS1
RBMS3
RBX1

RC3H2
RCAN1
RCBTB2
RCCD1
RCL1
RCN2
RCN3
RCOR1
RD3
RDH11
RDH14
RDM1
RDX
RECK
RECQL4
REG1A
REG3A
RELB
RELN
REM1
REP15
REPIN1
RERE
RERG
RERGL
RET
RETNLB
REV1
REXO1L2P
REXO2
REXO4
RFC3
RFESD
RFFL
RFPL4A
RFPL4B
RFT1
RFTN1
RFWD2
RFX2
RFX3
RFX6
RFX7
RFX8
RGAG1
RGL4
RGMA
RGMB
RGN
RGP1
RGPD5
RGS1
RGS12
RGS13

RGS16
RGS18
RGS2
RGS20
RGS22
RGS3
RGS5
RGS6
RGS7
RGS7BP
RGS9BP
RHBDD1
RHBDL1
RHCG
RHOBTB1
RHOBTB2
RHOF
RHOJ
RHOU
RHPN2
RIBC2
RIC8B
RICTOR
RIMKLB
RIMS1
RIMS2
RIMS4
RIN2
RIOK2
RIPK4
RIPPLY3
RIT2
RLF
RLTPR
RMDN2
RMST
RNASE1
RND3
RNF111
RNF138
RNF14
RNF144A
RNF145
RNF150
RNF165
RNF168
RNF17
RNF183
RNF185
RNF2
RNF213
RNF217-AS1
RNF220
RNF222

RNF31
RNF32
RNF34
RNF38
RNF4
RNF41
RNF5P1
RNFT2
RNGTT
RNLS
RNPEP
RNU11
ROBO1
ROBO2
ROCK1
ROCK2
ROMO1
ROR1
ROR2
RORA
RORC
ROS1
RP1
RP1L1
RPA1
RPA3
RPA4
RPAP3
RPE
RPF1
RPH3A
RPH3AL
RPL10A
RPL13A
RPL13AP6
RPL21
RPL21P44
RPL22
RPL22L1
RPL23A
RPL23P8
RPL27A
RPL36AL
RPL37
RPL37A
RPL6
RPL7
RPLP1
RPN1
RPN2
RPP21
RPP25
RPP25L
RPRD1A

RPRD1B
RPRM
RPS13
RPS29
RPS3
RPS6KA1
RPS6KA2
RPS6KA3
RPS6KA4
RPS6KA5
RPS6KA6
RPS6KB2
RPS6KC1
RPS7
RPSAP52
RPUSD2
RRAGB
RRAGC
RRAGD
RRBP1
RREB1
RRH
RRNAD1
RSAD2
RSBN1L
RSPH14
RSPO2
RSPO3
RSU1
RTCA
RTN1
RTN2
RTN4
RTN4IP1
RTP1
RTP3
RTP4
RUNDC1
RUNDC3B
RUNX2
RUNX3
RUSC2
RUVBL1
RWDD3
RXFP1
RXFP2
RXRA
RYBP
RYR1
RYR2
RYR3
S100A11
S100A12
S1PR2

S1PR5
SAG
SALL3
SAMD11
SAMD12
SAMD13
SAMD4A
SAMD4B
SAMD8
SAMHD1
SAMSN1
SAP130
SAR1B
SARDH
SARNP
SARS2
SASH1
SASH3
SATB2
SBF2
SBK1
SBK2
SBNO1
SBNO2
SC5D
SCAF4
SCAF8
SCAI
SCAMP2
SCAMP3
SCAMP4
SCAP
SCAPER
SCARA5
SCARB1
SCARF2
SCARNA2
SCARNA27
SCARNA3
SCG2
SCHIP1
SCIN
SCLT1
SCMH1
SCML1
SCN10A
SCN1B
SCN2A
SCN2B
SCN5A
SCN7A
SCN8A
SCNN1A
SCNN1B

SCP2
SCPEP1
SCRN1
SCUBE2
SDC1
SDC2
SDC4P
SDCCAG8
SDF2
SDHA
SDHAF4
SDK1
SDK2
SDPR
SDR39U1
SDS
SEC11C
SEC14L4
SEC14L5
SEC16B
SEC22C
SEC24B
SEC24D
SEC61G
SEL1L
SEL1L3
SELO
SELT
SEMA3A
SEMA3D
SEMA6A
SEMA6D
SENP1
SENP7
SEPN1
SEPP1
SEPT3
SEPT5
SEPT6
SERBP1
SERGEF
SERHL
SERINC3
SERINC5
SERP2
SERPINA1
SERPINA10
SERPINA12
SERPINA13P
SERPINB1
SERPINB2
SERPINB4
SERPINB9
SERPINC1

SERPINH1
SERPINI1
SERPINI2
SERTAD3
SESN3
SESTD1
SETBP1
SETD1A
SETD3
SETD5
SETD7
SETX
SF3A1
SF3B5
SFMBT1
SFMBT2
SFPQ
SFR1
SFRP1
SFRP5
SFTPB
SFXN2
SGCD
SGCZ
SGIP1
SGK1
SGK2
SGK3
SGOL2
SGPP1
SGPP2
SGSH
SGSM1
SH2D4A
SH2D4B
SH2D7
SH3BGR
SH3BGRL
SH3BGRL3
SH3BP2
SH3BP4
SH3D19
SH3D21
SH3GL1
SH3PXD2A
SH3PXD2B
SH3RF1
SH3RF2
SH3TC1
SH3TC2
SHANK2-AS3
SHC3
SHCBP1L
SHE

SHF
SHFM1
SHISA2
SHISA3
SHISA9
SHKBP1
SHMT2
SHPK
SHROOM2
SHROOM3
SIDT1
SIGIRR
SIGLEC1
SIGLEC15
SIGLEC6
SIGLEC8
SIK2
SIK3
SIM1
SIN3A
SIN3B
SIPA1L3
SIRPA
SIX3
SIX4
SIX6
SKAP1
SKI
SKIL
SKINTL
SKOR1
SKP2
SLAIN1
SLAIN2
SLBP
SLC10A1
SLC10A2
SLC10A6
SLC10A7
SLC12A8
SLC13A3
SLC13A4
SLC13A5
SLC14A1
SLC14A2
SLC15A1
SLC16A1
SLC16A14
SLC16A5
SLC16A7
SLC17A2
SLC17A4
SLC17A6
SLC18A1

SLC18A3
SLC19A1
SLC19A3
SLC1A1
SLC1A2
SLC1A3
SLC20A1
SLC20A2
SLC22A1
SLC22A10
SLC22A15
SLC22A2
SLC22A23
SLC22A25
SLC22A3
SLC22A5
SLC22A8
SLC23A2
SLC24A5
SLC25A10
SLC25A13
SLC25A15
SLC25A17
SLC25A18
SLC25A20
SLC25A21
SLC25A26
SLC25A3
SLC25A30
SLC25A32
SLC25A34
SLC25A35
SLC25A36
SLC25A37
SLC25A4
SLC25A45
SLC25A46
SLC25A47
SLC25A6
SLC26A1
SLC26A3
SLC26A5
SLC26A7
SLC27A3
SLC29A4
SLC2A1
SLC2A10
SLC2A12
SLC2A13
SLC2A7
SLC2A9
SLC30A1
SLC30A10
SLC30A3

SLC30A6
SLC30A8
SLC31A2
SLC32A1
SLC33A1
SLC35B4
SLC35C1
SLC35C2
SLC35F1
SLC35F2
SLC35F4
SLC36A1
SLC36A3
SLC37A1
SLC37A2
SLC38A1
SLC38A2
SLC38A4
SLC38A9
SLC39A10
SLC39A11
SLC39A6
SLC39A8
SLC41A1
SLC41A2
SLC43A1
SLC44A1
SLC44A2
SLC44A3
SLC46A1
SLC46A2
SLC4A1
SLC4A10
SLC4A4
SLC4A7
SLC51A
SLC5A11
SLC5A12
SLC5A4
SLC5A7
SLC5A8
SLC5A9
SLC6A1
SLC6A11
SLC6A2
SLC6A5
SLC6A6
SLC6A9
SLC7A1
SLC7A11
SLC7A14
SLC7A6
SLC7A9
SLC8A1

SLC9A2
SLC9A3R1
SLC9A5
SLC9A6
SLC9A7
SLC9A8
SLC9A9
SLC9C1
SLC9C2
SLCO1A2
SLCO1C1
SLCO2A1
SLCO3A1
SLCO4A1
SLCO5A1
SLCO6A1
SLFN12
SLFNL1
SLIT1
SLIT2
SLIT3
SLITRK1
SLITRK3
SLITRK5
SLITRK6
SLK
SLN
SLX4IP
SMAD3
SMAD4
SMAD5
SMAD6
SMAP1
SMARCA1
SMARCA2
SMARCA4
SMARCC1
SMARCD3
SMC2
SMC3
SMC4
SMC6
SMCO1
SMCO2
SMDT1
SMG5
SMG6
SMG7
SMIM14
SMIM15
SMIM20
SMIM3
SMOC1
SMOC2

SMPD1
SMPDL3A
SMPDL3B
SMS
SMTNL2
SMURF1
SMYD3
SNAI1
SNAI2
SNAP23
SNAP29
SNAP47
SNAPC3
SNAR-I
SNCA
SND1
SNHG11
SNORA14A
SNORA16B
SNORA21
SNORA33
SNORA38B
SNORA70C
SNORA70F
SNORA71D
SNORA72
SNORA80A
SNORA80B
SNORD109B
SNORD115-26
SNORD115-28
SNORD115-37
SNORD115-44
SNORD116-9
SNORD12
SNORD15A
SNORD1C
SNORD50B
SNORD87
SNORD94
SNRK
SNRNP27
SNRNP40
SNRPA
SNRPB
SNRPF
SNRPG
SNTB1
SNTG1
SNTG2
SNTN
SNX1
SNX12
SNX13

SNX14
SNX20
SNX21
SNX25
SNX30
SNX31
SNX4
SNX5
SOCS1
SOCS3
SOCS6
SOD1
SOGA1
SOGA3
SON
SORBS1
SORBS2
SORCS1
SORCS2
SORL1
SOX10
SOX11
SOX13
SOX17
SOX2
SOX2-OT
SOX21
SOX5
SOX6
SOX8
SOX9
SP100
SP110
SP3
SP4
SP8
SPAG1
SPAG16
SPAG4
SPAG6
SPARC
SPARCL1
SPAST
SPATA13
SPATA16
SPATA17
SPATA19
SPATA2
SPATA20
SPATA21
SPATA25
SPATA2L
SPATA5
SPATA6

SPATS2L
SPCS3
SPDYC
SPDYE5
SPECC1
SPEF2
SPESP1
SPG11
SPG20
SPG21
SPG7
SPHAR
SPIC
SPIDR
SPIN1
SPIN3
SPINK5
SPIRE1
SPOCK1
SPON1
SPPL2A
SPPL2B
SPPL3
SPR
SPRED1
SPRED2
SPRED3
SPRN
SPRR1A
SPRR2B
SPRR2C
SPRR2F
SPRR2G
SPRR3
SPRR4
SPRY1
SPRY2
SPRY4
SPRYD3
SPRYD7
SPTA1
SPTB
SPTBN1
SPTBN2
SPTSSA
SQRDL
SRCAP
SRD5A1
SRD5A2
SREBF2
SREK1IP1
SRFBP1
SRGAP3
SRP54

SRP68
SRP9
SRPK1
SRPK2
SRPX
SRRM1
SRRM2
SRRM4
SRSF9
SRXN1
SS18
SSBP1
SSBP2
SSBP4
SSH1
SSH2
SSR4
SSR4P1
SST
SSTR1
SSTR4
SSX2IP
ST14
ST18
ST3GAL1
ST3GAL3
ST3GAL5
ST5
ST6GAL1
ST6GAL2
ST6GALNAC1
ST6GALNAC3
ST6GALNAC5
ST7
ST7-AS2
ST7L
ST8SIA2
ST8SIA5
STAB2
STAG1
STAG3L4
STAM2
STAMBP
STAP1
STARD10
STARD13
STARD3NL
STARD4
STARD5
STARD6
STAT1
STAT4
STATH
STAU2

STC2
STEAP3
STEAP4
STIL
STIM1
STIM2
STIP1
STK24
STK26
STK31
STK32B
STK33
STK4
STKLD1
STMN2
STMN4
STOM
STON2
STOX1
STOX2
STPG1
STPG2
STRA6
STRA8
STRBP
STRC
STRIP1
STRN
STRN3
STS
STT3A
STT3B
STX10
STX18
STX4
STX6
STX7
STX8
STXBP4
STXBP5L
STXBP6
SUCLG2
SUDS3
SUFU
SUGCT
SUGT1P3
SULF1
SULT1C2
SULT1C4
SULT1E1
SULT2B1
SUMF1
SUMO1
SUMO1P1

SUPT16H
SUPT3H
SUPT5H
SURF4
SUSD4
SUSD5
SUV420H1
SV2A
SV2B
SV2C
SVEP1
SVIL
SVIP
SVOP
SWAP70
SWT1
SYBU
SYCP1
SYK
SYN2
SYN3
SYNDIG1L
SYNE1
SYNE3
SYNGAP1
SYNJ1
SYNRG
SYPL1
SYT1
SYT10
SYT12
SYT13
SYT14
SYT2
SYT4
SYT7
SYTL2
T
TAC3
TAC4
TACC2
TACC3
TACR1
TADA2A
TAF12
TAF1A
TAF1B
TAF4
TAF6
TAF8
TAGLN
TAGLN3
TANC1
TAOK1

TAP1
TAPT1
TARBP1
TARS
TAS2R14
TAS2R16
TAS2R42
TASP1
TAX1BP1
TBC1D1
TBC1D12
TBC1D13
TBC1D15
TBC1D16
TBC1D22A
TBC1D22B
TBC1D24
TBC1D25
TBC1D31
TBC1D32
TBC1D4
TBC1D5
TBC1D7
TBC1D8
TBC1D9
TBCC
TBCE
TBCK
TBKBP1
TBL1XR1
TBL1Y
TBRG1
TBX1
TBX15
TBX19
TBX2
TBX21
TBX3
TBX5
TBXAS1
TCAM1P
TCEB1
TCERG1
TCERG1L
TCF12
TCF15
TCF20
TCF3
TCF7L2
TCFL5
TCHHL1
TCHP
TCIRG1
TCL6

TCN1
TCP1
TCP10L
TCP11L2
TCTEX1D2
TCTN2
TCTN3
TDP1
TDRD1
TDRD10
TDRD6
TDRP
TEAD3
TECRL
TECTA
TECTB
TEKT4
TENM1
TERC
TERT
TET1
TEX101
TEX14
TEX15
TEX2
TEX26
TEX261
TEX30
TEX36
TF
TFAP2A
TFAP2C
TFAP2D
TFAP4
TFCP2L1
TFEB
TFEC
TFPI
TFRC
TG
TGFBR3
TGFBRAP1
TGM2
TGOLN2
THADA
THAP9
THBD
THBS1
THBS2
THOC5
THPO
THRA
THRAP3
THRB

THSD4
THSD7A
THUMPD2
TIAM1
TIAM2
TIFA
TIGD6
TIMM17B
TIMM21
TIPARP
TIPIN
TJP1
TJP3
TK2
TKT
TKTL2
TLE1
TLE3
TLE4
TLE6
TLK1
TLL1
TLL2
TLN1
TLN2
TLR10
TLR3
TLR4
TLR5
TLR8
TM4SF18
TM4SF19
TM4SF20
TM6SF1
TM7SF2
TM7SF3
TM9SF2
TM9SF3
TMC1
TMC2
TMC3
TMC5
TMC6
TMC7
TMCC2
TMCC3
TMCO1
TMCO4
TMCO5A
TMED10P1
TMED4
TMED8
TMEFF2
TMEM102

TMEM105
TMEM108
TMEM109
TMEM11
TMEM110
TMEM114
TMEM117
TMEM121
TMEM126A
TMEM132B
TMEM132C
TMEM132D
TMEM132E
TMEM134
TMEM135
TMEM138
TMEM140
TMEM144
TMEM151A
TMEM154
TMEM160
TMEM161B
TMEM163
TMEM164
TMEM17
TMEM170B
TMEM189
TMEM194A
TMEM198
TMEM2
TMEM200A
TMEM200B
TMEM205
TMEM206
TMEM207
TMEM216
TMEM220
TMEM229B
TMEM252
TMEM255A
TMEM257
TMEM259
TMEM26
TMEM260
TMEM263
TMEM27
TMEM33
TMEM35
TMEM37
TMEM38B
TMEM39A
TMEM42
TMEM43
TMEM44

TMEM45A
TMEM57
TMEM59L
TMEM60
TMEM62
TMEM63C
TMEM64
TMEM67
TMEM69
TMEM71
TMEM72
TMEM8A
TMEM9
TMEM98
TMEM99
TMF1
TMPO
TMPRSS11D
TMPRSS2
TMPRSS5
TMSB4XP8
TMSB4Y
TMTC1
TMTC2
TMTC3
TMX3
TMX4
TNFAIP2
TNFAIP3
TNFAIP6
TNFAIP8
TNFAIP8L3
TNFRSF10B
TNFRSF11A
TNFRSF11B
TNFRSF19
TNFRSF1A
TNFRSF21
TNFRSF8
TNFSF10
TNFSF11
TNFSF13B
TNFSF18
TNIK
TNK1
TNK2
TNKS2
TNN
TNNI3K
TNPO2
TNRC6A
TNRC6B
TNS1
TNS3

TOB1
TOM1L1
TOMM20
TOMM70A
TOP1
TOP1P1
TOP1P2
TOP2B
TOP3B
TOPAZ1
TOPBP1
TOPORS
TOX
TP53BP2
TP53I11
TP53INP1
TP53RK
TP53TG1
TP63
TP73
TPCN1
TPD52
TPH1
TPH2
TPK1
TPO
TPP2
TPRG1
TPRG1L
TPRKB
TPST1
TPT1
TPTE2P1
TPX2
TRAF3IP2
TRAF7
TRAFD1
TRAM1L1
TRAM2
TRAP1
TRAPPC11
TRAPPC12
TRAPPC3
TRAPPC9
TRDMT1
TREH
TREM1
TREML4
TRERF1
TRHDE
TRIAP1
TRIB1
TRIM13
TRIM2

TRIM24
TRIM25
TRIM31
TRIM32
TRIM33
TRIM35
TRIM36
TRIM48
TRIM52
TRIM53AP
TRIM58
TRIM7
TRIM71
TRIO
TRIP10
TRIP11
TRIP12
TRIQK
TRMT10A
TRMT11
TRMT12
TRMU
TRNAU1AP
TRPA1
TRPC1
TRPC3
TRPC4AP
TRPC5
TRPC6
TRPM1
TRPM7
TRPM8
TRPV3
TRPV5
TRRAP
TSC2
TSC22D1
TSC22D3
TSEN15
TSGA10IP
TSHB
TSHZ1
TSHZ2
TSKU
TSNARE1
TSNAXIP1
TSPAN14
TSPAN15
TSPAN32
TSPAN33
TSPAN6
TSPAN7
TSPAN8
TSPAN9

TSPEAR
TSPO
TSR1
TSSC1
TSSC4
TSSK6
TTBK1
TTC1
TTC13
TTC17
TTC21A
TTC21B
TTC22
TTC26
TTC27
TTC28
TTC29
TTC3
TTC30A
TTC38
TTC39B
TTC5
TTC7A
TTC8
TTI1
TTK
TTLL11
TTLL4
TTLL5
TTLL7
TTN
TTTY3B
TTYH3
TUBA3E
TUBB2B
TUBB7P
TUBD1
TUBGCP3
TULP3
TUSC1
TUSC3
TUSC5
TUT1
TWF2
TXK
TXLNGY
TXNDC16
TXNDC17
TXNDC5
TXNIP
TXNL1
TXNRD1
TXNRD3NB
TYRP1

TYSND1
TYW1
U2AF2
U2SURP
UACA
UAP1
UBAC1
UBAC2
UBALD1
UBAP1
UBAP2
UBASH3A
UBASH3B
UBE2E2
UBE2E3
UBE2F
UBE2G1
UBE2L3
UBE2L6
UBE2M
UBE2MP1
UBE2Q2
UBE2R2
UBE2W
UBE3B
UBE3C
UBE4A
UBIAD1
UBL3
UBL7
UBLCP1
UBN1
UBR3
UBR4
UBXN2A
UBXN2B
UCHL1
UCHL3
UCK2
UCP1
UFM1
UGGT1
UGP2
UGT1A7
UGT1A8
UGT2A1
UGT2A3
UGT2B10
UGT2B11
UGT2B17
UGT2B28
UGT2B7
UGT3A2
UGT8

UHRF2
ULBP2
ULBP3
ULK3
ULK4P1
UNC13B
UNC45B
UNC5D
UNC79
UNC80
UNC93B1
UNG
UPF1
UPF2
UPP1
UPP2
UQCC1
UQCRC1
UQCRC2
UQCRQ
URB2
URI1
USF2
USH2A
USMG5
USP10
USP12
USP13
USP20
USP24
USP25
USP26
USP3
USP31
USP32
USP37
USP39
USP40
USP43
USP50
USP54
USP7
USP8
USP9X
USP9Y
UST
UTP14C
UTRN
UTS2
UVRAG
VAC14
VAMP3
VANGL1
VAPA

VASH1
VAV2
VAV3
VCAN
VCL
VCPIP1
VDAC1
VDAC2
VEGFA
VEGFC
VEPH1
VEZF1
VEZT
VGLL3
VIPR1
VIPR2
VIT
VLDLR
VN1R2
VN1R4
VNN2
VPS13A
VPS13C
VPS13D
VPS33A
VPS33B
VPS37C
VPS39
VPS41
VPS53
VPS8
VRTN
VSIG4
VSTM2L
VSTM5
VTCN1
VTI1A
VWA2
VWA3A
VWA5A
VWA8
VWC2
VWF
WAC
WAPAL
WARS2
WAS
WASH2P
WASL
WBP4
WBSCR17
WBSCR27
WDFY2
WDFY3-AS2

WDPCP
WDR1
WDR11
WDR13
WDR19
WDR26
WDR4
WDR41
WDR43
WDR47
WDR48
WDR53
WDR5B
WDR64
WDR70
WDR72
WDR75
WDR82
WDR86
WDR88
WDR92
WDSUB1
WEE1
WFDC10B
WFDC13
WFDC2
WFDC8
WFIKKN2
WFS1
WHAMMP2
WIBG
WIF1
WIPF1
WISP3
WIZ
WLS
WNK1
WNK2
WNT10B
WNT11
WNT2
WNT3
WNT3A
WNT5A
WNT5B
WNT7B
WNT9B
WSB1
WT1
WTAP
WWC1
WWC3
WWOX
WWP2

WWTR1
XAB2
XAF1
XGY2
XKR4
XKR5
XKR6
XKR8
XKR9
XKRX
XPNPEP1
XPO6
XPO7
XPOT
XRCC1
XRCC6
XRCC6BP1
XRN2
XXYLT1
YAP1
YDJC
YIF1B
YIPF5
YRDC
YTHDC1
YTHDC2
YTHDF2
YWHAB
YY1
ZAK
ZBED5
ZBP1
ZBTB1
ZBTB10
ZBTB12
ZBTB16
ZBTB2
ZBTB20
ZBTB24
ZBTB26
ZBTB32
ZBTB34
ZBTB43
ZBTB44
ZBTB45
ZBTB46
ZBTB7A
ZBTB8A
ZBTB8B
ZBTB9
ZC2HC1C
ZC3H10
ZC3H12C
ZC3H15

ZC3H7A
ZC3H7B
ZC3HAV1
ZC4H2
ZCCHC16
ZCCHC2
ZCCHC5
ZCCHC7
ZCWPW2
ZDHHC1
ZDHHC11
ZDHHC14
ZDHHC16
ZDHHC2
ZDHHC21
ZDHHC23
ZDHHC6
ZDHHC7
ZEB2
ZFAND2A
ZFAND3
ZFAND6
ZFHX2
ZFHX4
ZFP30
ZFP36L1
ZFP36L2
ZFP41
ZFP64
ZFP91
ZFPM1
ZFPM2
ZFYVE16
ZFYVE26
ZFYVE27
ZFYVE28
ZFYVE9
ZHX3
ZIC1
ZIC2
ZIC3
ZIC4
ZIC5
ZIM2
ZIM3
ZKSCAN1
ZMAT3
ZMAT4
ZMIZ1
ZMIZ2
ZMYM4
ZMYM5
ZMYND8
ZNF107

ZNF114
ZNF131
ZNF137P
ZNF143
ZNF148
ZNF160
ZNF17
ZNF192P1
ZNF200
ZNF202
ZNF217
ZNF219
ZNF22
ZNF221
ZNF222
ZNF229
ZNF23
ZNF230
ZNF234
ZNF250
ZNF252P-AS1
ZNF253
ZNF266
ZNF271P
ZNF273
ZNF274
ZNF277
ZNF280A
ZNF280C
ZNF286A
ZNF319
ZNF32
ZNF322
ZNF322P1
ZNF326
ZNF329
ZNF337
ZNF341
ZNF362
ZNF365
ZNF37BP
ZNF383
ZNF385B
ZNF395
ZNF396
ZNF397
ZNF407
ZNF423
ZNF436
ZNF438
ZNF451
ZNF462
ZNF480
ZNF483

ZNF486
ZNF496
ZNF503-AS2
ZNF517
ZNF518A
ZNF521
ZNF546
ZNF549
ZNF551
ZNF557
ZNF563
ZNF565
ZNF566
ZNF579
ZNF580
ZNF582
ZNF585A
ZNF589
ZNF592
ZNF595
ZNF596
ZNF609
ZNF610
ZNF618
ZNF621
ZNF630
ZNF639
ZNF641
ZNF645
ZNF652
ZNF660
ZNF677
ZNF678
ZNF683
ZNF684
ZNF695
ZNF697
ZNF703
ZNF71
ZNF740
ZNF746
ZNF767P
ZNF768
ZNF77
ZNF777
ZNF780B
ZNF781
ZNF784
ZNF787
ZNF792
ZNF804A
ZNF804B
ZNF805
ZNF816

ZNF831
ZNF835
ZNF93
ZNHIT2
ZNRF2
ZNRF4
ZPBP2
ZPLD1
ZSCAN12
ZSCAN18
ZSCAN2
ZSCAN20
ZSCAN22
ZSCAN23
ZSCAN31
ZWINT
ZXDA
ZZZ3
